# Supplementary material for: Using an on-site modular training approach to amplify prep service delivery in public health facilities in Kenya
Source: PLOS Glob Public Health. 2022 Mar 10;2(3):e0000092. doi: 10.1371/journal.pgph.0000092 (PMC10021257; doi:10.1371/journal.pgph.0000092)
Supplement: S1 Text — (PDF) [file pgph.0000092.s001.pdf]

Pre-Exposure Prophylaxis

NASCOP

April 2018

Module 1

Outline

- Background
- Update on ART guidelines
- Pre Vs Post Exposure Prophylaxis
- PrEP Efficacy
- PrEP for discordant couples
- Overview of Health service providers toolkit

Background

- In 2015, 71,034 Kenyans aged above 15 years and 6,613 aged 0-14 years got infected with HIV
- A third of all these new infections were among young women aged 15-24.
- Sixteen children acquired HIV through mother-to- child transmission daily in 2015

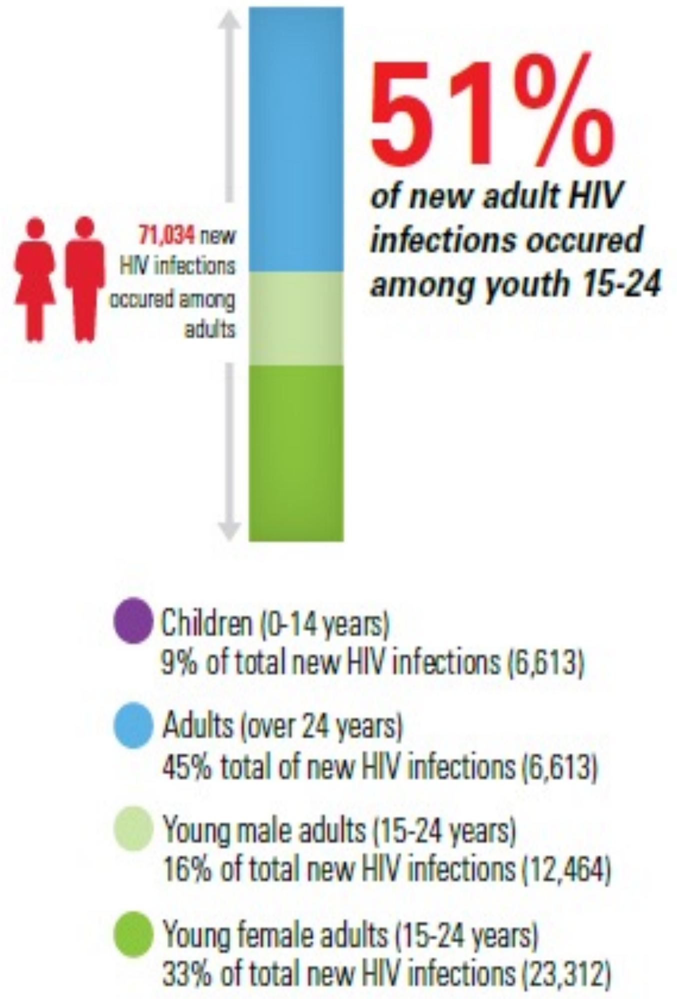

HIV Treatment

Antiretroviral drugs (ARVs) are effective in managing HIV disease

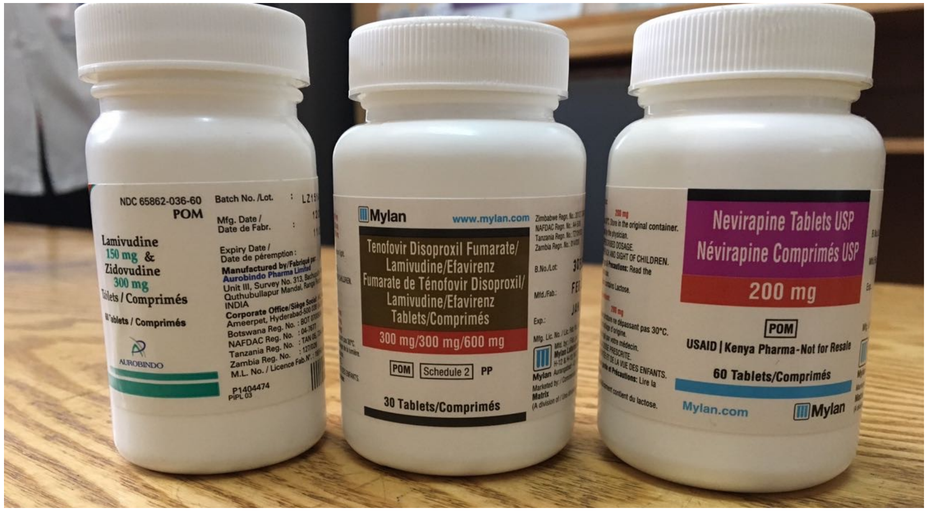

HIV Treatment

The goal of antiretroviral therapy (ART) is to suppress viral replication with the aim of reducing viral load to undetectable levels.

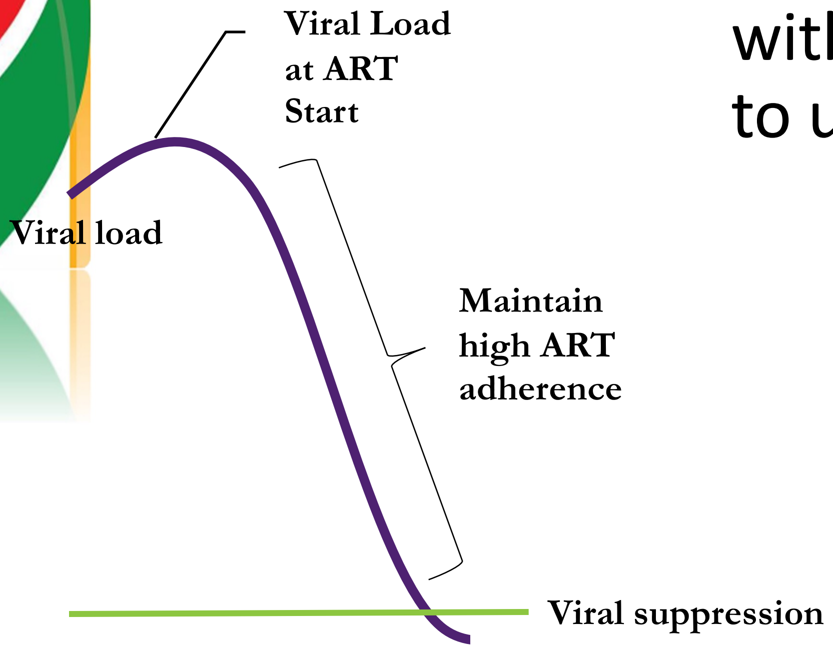

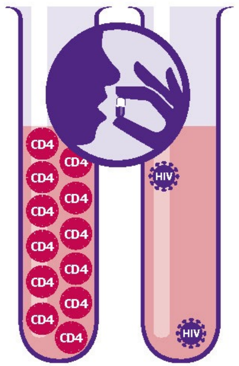

Source: NAM AIDSMap

Benefits of Undetectable Viral Load

- Prevents damage to immune system
- Restores and maintains healthy living
- Reduces risk of HIV transmission

Treatment as Prevention (TasP)

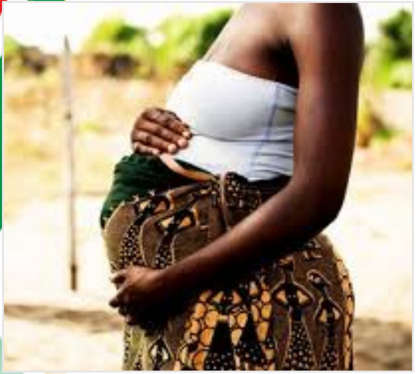

Source: Reuters

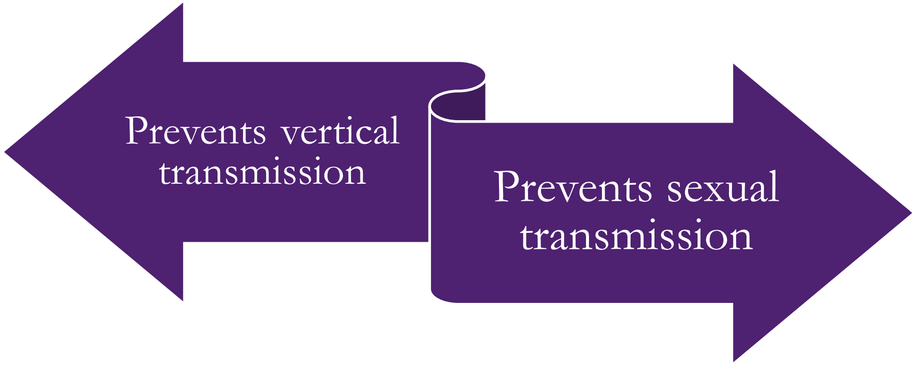

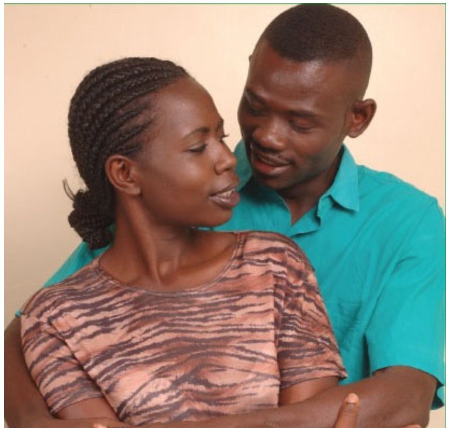

Source: PSI Sudan

Who is eligible for ART?

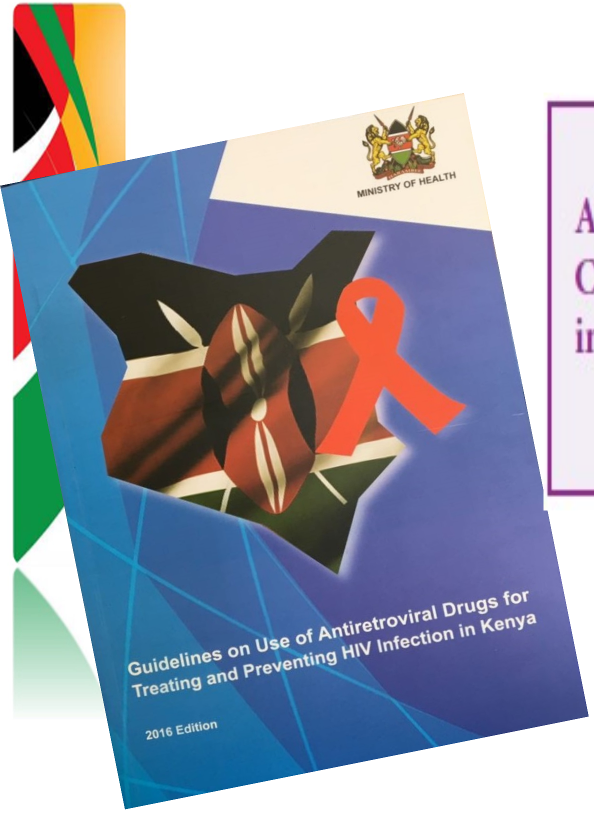

All individuals with confirmed HIV infection are eligible for ART, irrespective of CD4 cell levels, WHO clinical stage, age, pregnancy or breastfeeding status, co-infection status, risk group, or any other criteria.

ART should be initiated as soon as the patient is ready to start, preferably within two weeks of confirmation of HIV status.

Pre-Exposure Prophylaxis (PrEP)

PrEP is a way for people who don't have HIV but are at high risk to prevent HIV infection by taking a pill every day

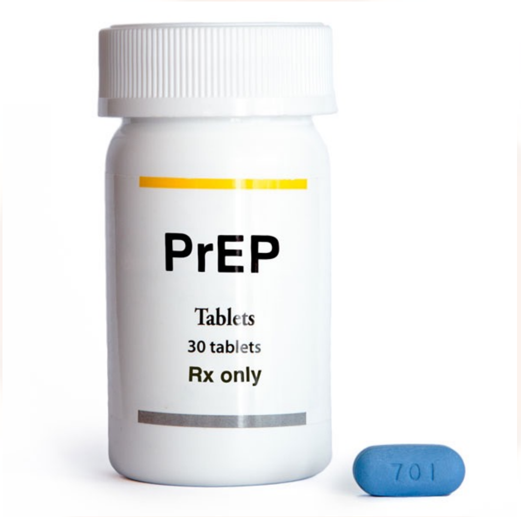

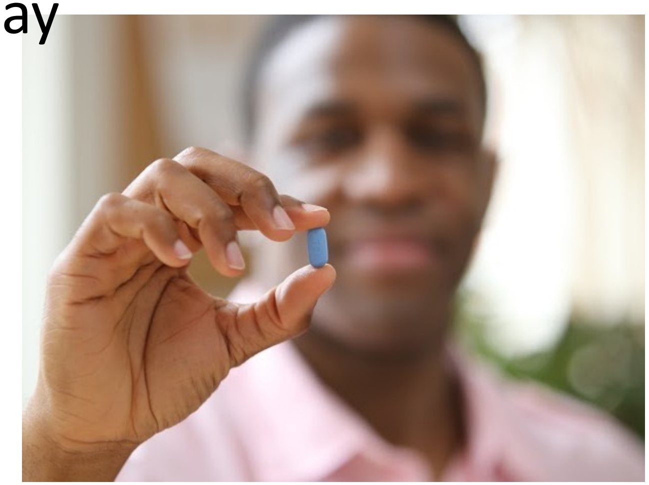

PEP vs PrEP

- Post-Exposure Prophylaxis (PEP) means taking ARVs **<u>after</u>** being potentially exposed to HIV to prevent becoming infected

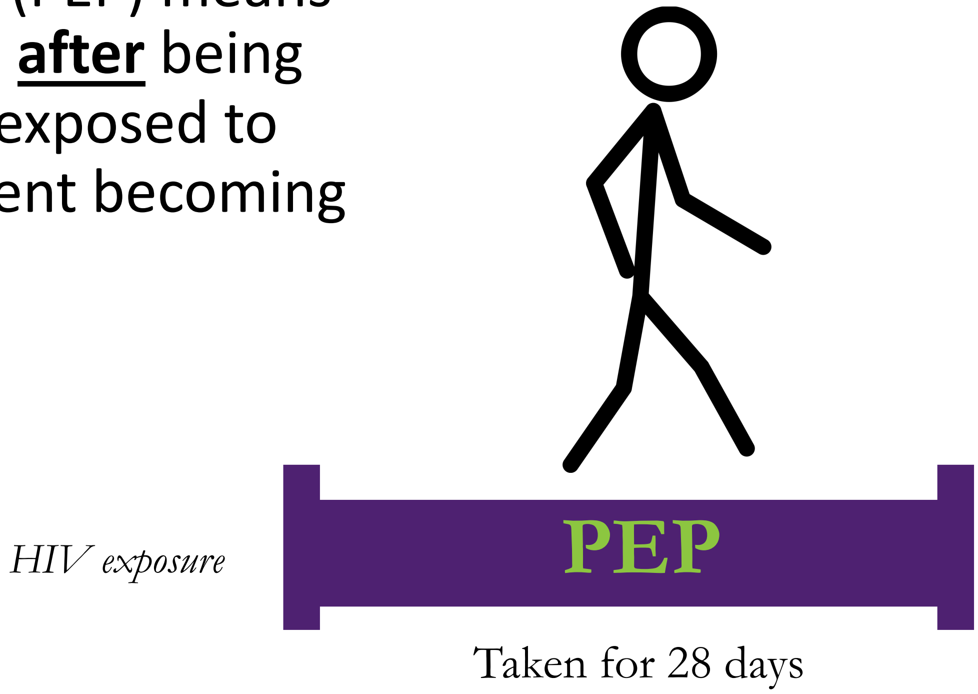

PEP vs PrEP

- PrEP is taken daily during 'seasons of risk'
- Takes 7 doses before protection begins and must continue daily during seasons of risk
- PrEP can be stopped when the risk is no longer there

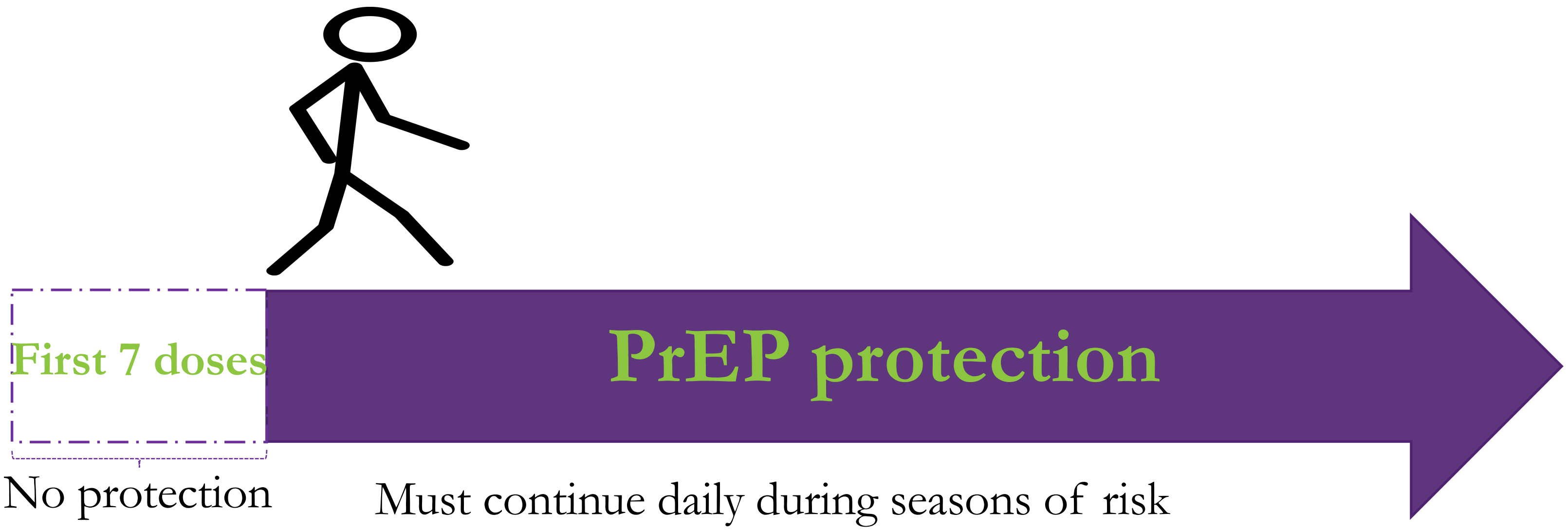

Recommended ARVs for PrEP

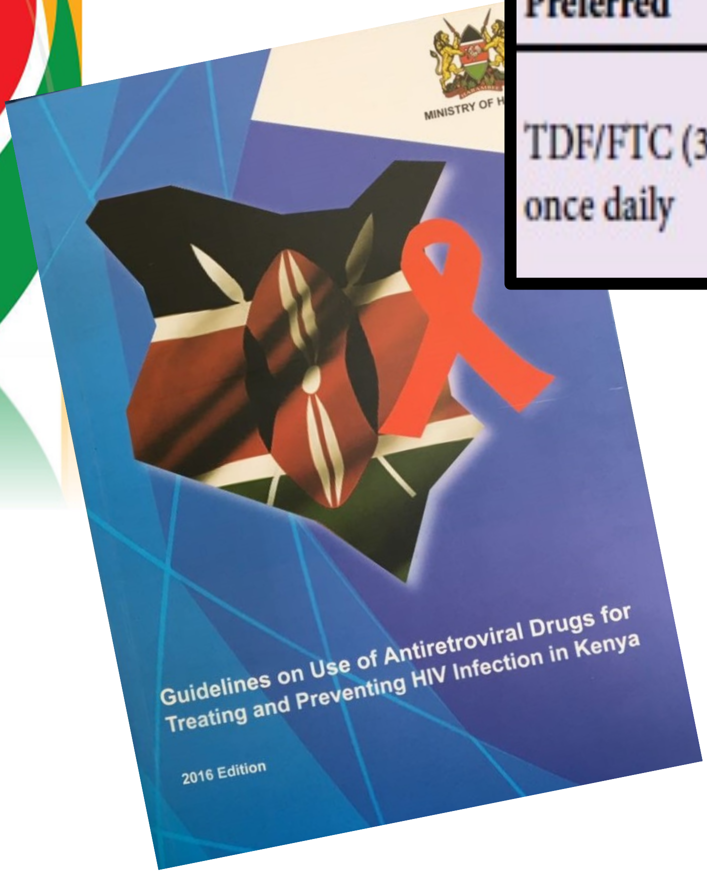

Preferred	Alternative
TDF/FTC (300 mg/200 mg) as FDC once daily	TDF 300 mg once daily
	TDF/3TC 300 mg/300 mg as FDC once daily

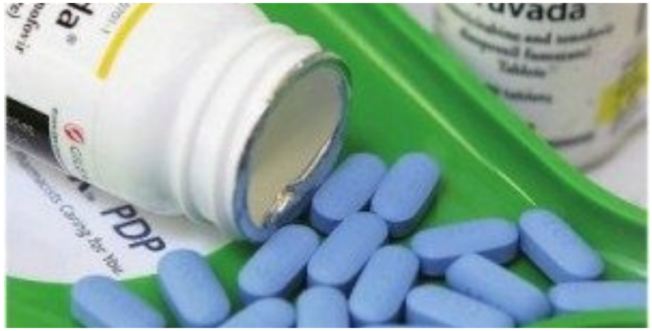

PrEP is Safe in Pregnancy

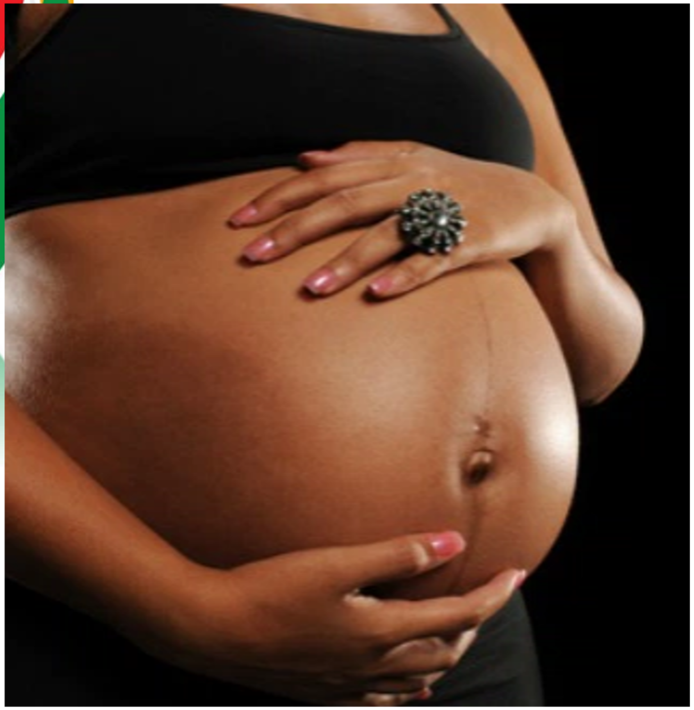

Source: http://www.shutterstock.com/

- PrEP can be used by women and men who want to have a baby
- PrEP can be used by women who
  - Are pregnant
  - Are breastfeeding

PrEP has Few Side Effects

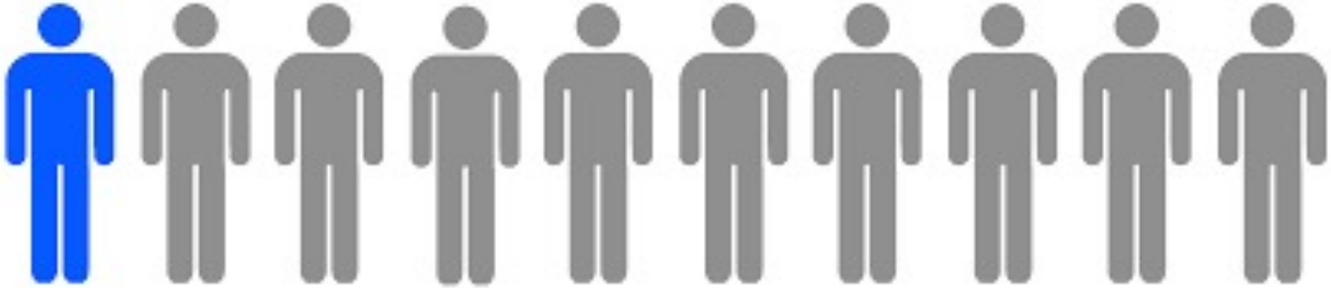

Gastrointestinal symptoms → Subside in 1-2 weeks

Low Risk of Resistance

Can only occur if there is HIV infection

Rare with PrEP use

Does not occur with PrEP adherence

Benefits of PrEP

Decreased anxiety

Increased communication, disclosure, & trust

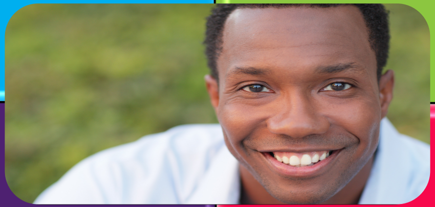

Increased self-efficacy

Increased sexual pleasure & intimacy

Benefits of PrEP for Serodiscordant Couples

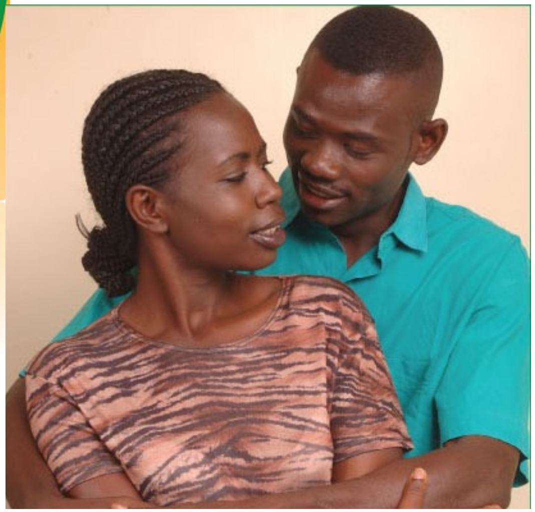

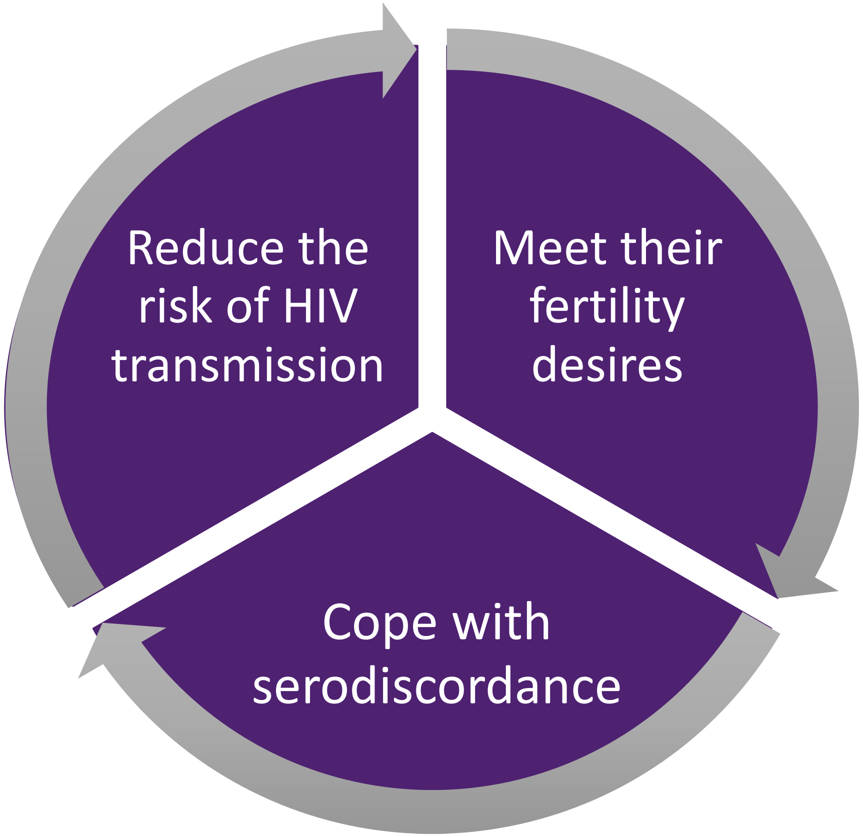

Evidence of PrEP efficacy

- Overall, with strict daily adherence studies have shown PrEP is over 90 percent effective at preventing HIV infection.
- With poor adherence effectiveness is not assured.

PrEP as a Bridge to ART

- HIV uninfected partners in serodiscordant partnerships are at high risk of HIV infection
- PrEP for the HIV uninfected partner reduces risk of becoming infected
- Early ART for the HIV infected partner reduces the risk of transmission

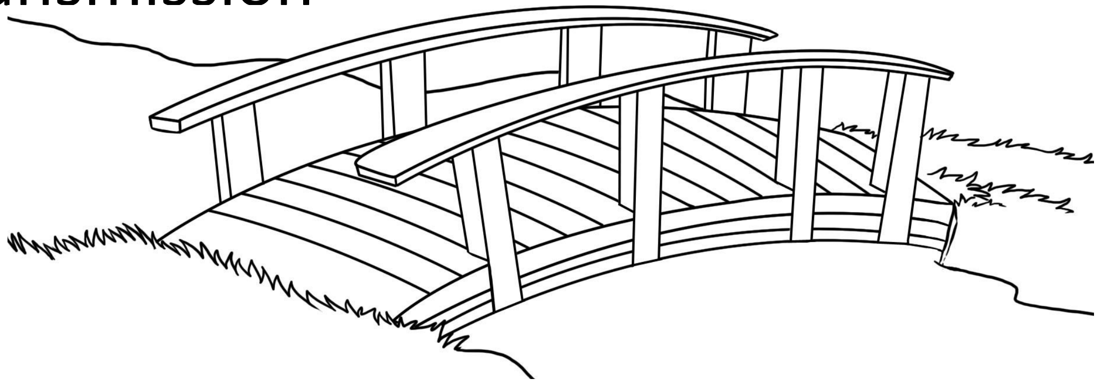

Source: DragoArt

PrEP as a bridge to ART for discordant couples

- For couples initiate ART at enrollment, PrEP will be offered through 6 months, then stopped:

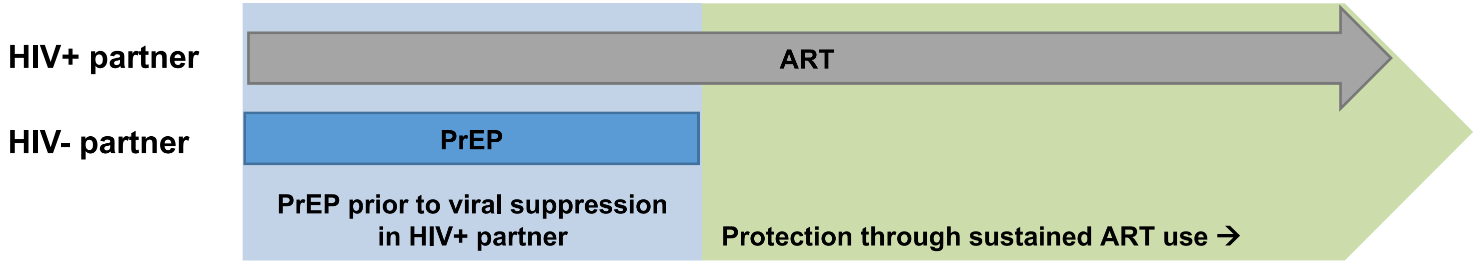

- For couples in which the infected partner delays or declines ART, PrEP will be continued until 6 months after ART initiation:

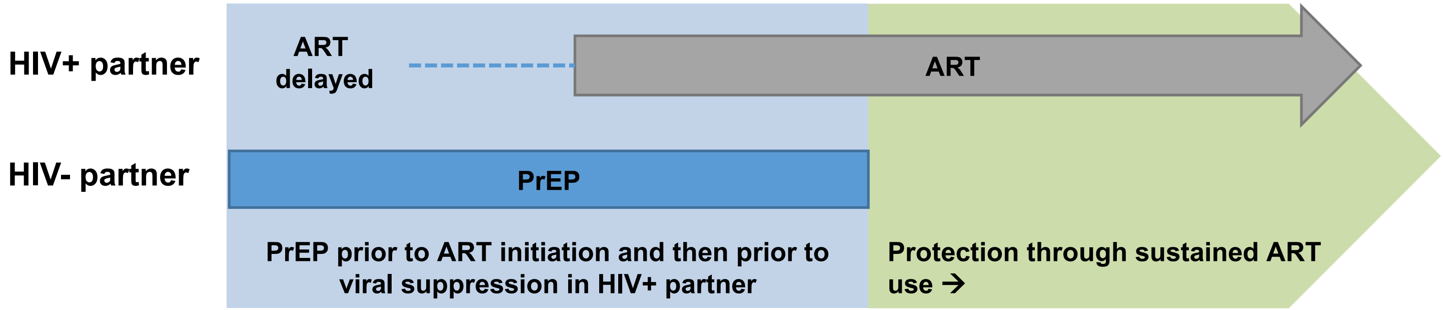

-)

PrEP Eligibility

- HIV-negative individuals at high risk of getting HIV infection, including:
  - Sexual partner of unknown status and at risk for HIV
  - Individuals engaging in transactional sex
  - Individuals with recent history of STIs
  - Clients with recurrent use of PEP
  - Habit of having sex under influence of drugs/alcohol
  - Inconsistent or no condom use
  - Injection drug use

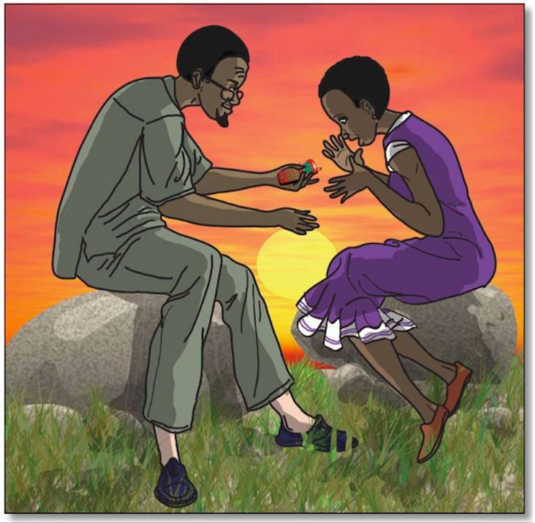

Source: "Addressing Cross-Generational Sex." Population Reference Bureau.

PrEP Eligibility among Serodiscordant Couples

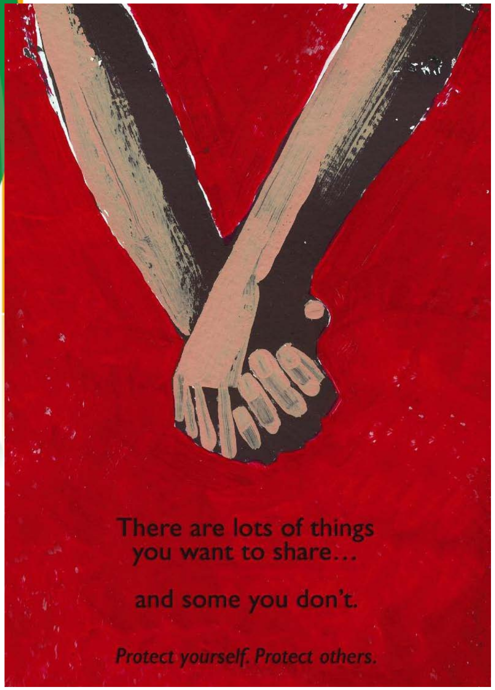

Source: Act Against AIDS

The HIV uninfected partner is eligible for PrEP if:

- The HIV *infected* partner:
  - Is not on ART
  - Is on ART < 6 months
  - Is suspected to have poor adherence to ART
  - Has a detectable viral load
- Couple is trying to get pregnant

PrEP Discontinuation

- PrEP should be discontinued if any of the below criteria are met:
  - HIV infection (seroconversion)
  - Viral suppression of the HIV infected partner OR HIV infected partner on ART for > 6 months with high adherence
  - Change in HIV risk status (low risk)
  - Client requests to stop
  - Creatinine clearance < 50 ml/min
  - Sustained non-adherence

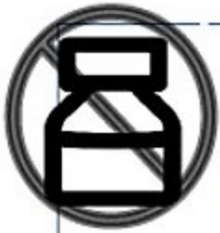

PrEP discontinuation. PrEP should be used when someone has a risk of getting HIV. When this risk is no longer there, PrEP can be stopped.

Safety Monitoring

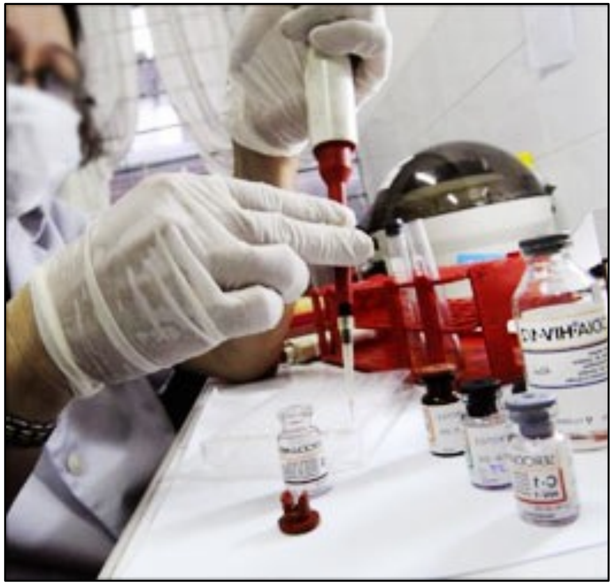

Source: MedIndia

Safety monitoring laboratory tests and procedures include:

- Hepatitis B testing
- Hepatitis C testing
- Annual serum creatinine and creatinine clearance (CrCl)

PrEP Use and Adherence

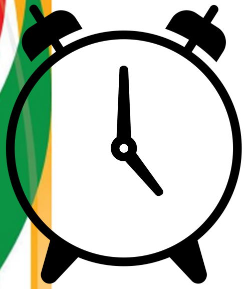

Daily use. PrEP must be taken every day to prevent HIV infection.

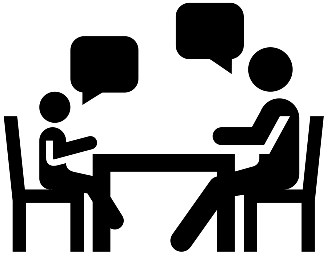

Adherence. Counselors can talk about the best strategies to help people take their daily pills. They help people find ways to take their pills during difficult times such as travel, in the face of stigma, or when there are new partners.

Seroconversion

Conduct HIV testing and counseling every 3 months

If the HIV test turns positive,

- Discontinue PrEP
- Counsel and immediately li
  HIV care and treatment

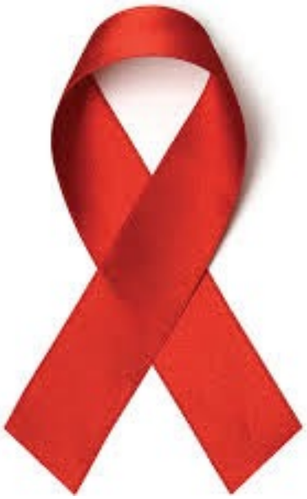

NASCOP

HARAMBEE

Module 2

Pre Exposure Prophylaxis for the prevention of HIV Infection- A Toolkit for Health Service Providers

Refer to your copy of the Toolkit through out this presentation

Outline of the Toolkit

1. Overview of recommendations for Pre-Exposure Prophylaxis to Prevent HIV Infection
2. Risk assessment and indications for Pre-Exposure Prophylaxis
3. Initiating Pre-exposure Prophylaxis
4. Follow-up and Monitoring of pre-exposure prophylaxis
5. Appendices

NASCOP

Overview of recommendations for PrEP

- Summary of the recommendations for PrEP (table 1.1)
- Schema for managing PrEP for HIV prevention (fig 1.1)
- Combination HIV Prevention
  - Entry point for PrEP (figure 1.2) (page 8)
  - PrEP should never be provided in isolation
  - Summary of steps for combination prevention for clients accessing PrEP services (fig 1.3)

Risk assessment and indications for PrEP

- Indications for PrEP
  - Assessing for “substantial ongoing” risk of HIV infection
  - General screening questions
  - Screening questions for people in discordant relationships
- Managing suspected acute HIV infection

Risk assessment and indications for PrEP

- Managing high risk exposure within the last 72 hours
  - Provide PEP for 28 days
  - Obtain rapid HIV test at 28 days, if negative transition to PrEP immediately (if the client has substantial on going risk of HIV infection)
- Screening for PrEP (figure 2.1)

Initiating Pre-Exposure Prophylaxis

- PrEP should only be initiated after
  - Figure 3.1 (schema for initiating PrEP)

- Eligibility for PrEP
  - Substantial on going risk of HIV infection
  - No suspicion of AHI
  - Documented HIV negative test
  - No contraindications to PrEP medications
  - Willingness to use PrEP as prescribed

Initiating Pre-Exposure Prophylaxis

- Initial assessment (Table 3.1)
- Managing Clinical and laboratory results (Table 3.2)
- Initial adherence preparation and counselling (Table 3.3)

Initiating Pre-Exposure Prophylaxis

- Pre-initiation education check list (table 3.4)
- Pre-initiation assessment check list (table 3.5)
  - Confirm that STI screening and treatment has been done prior to prescribing PrEP
- Preferred TDF 300mg/FTC 200 mg once daily (Table 3.6)
  - Alternative 1 TDF 300 mg once daily
  - Alternative 2 TDF 300/3TC 300 mg once daily

Initiating Pre-Exposure Prophylaxis

- Prescribing Pre-exposure prophylaxis
- First prescription should be for 30 days to allow for follow-up visit to assess
  - Adherence
  - Tolerability
  - Adverse effects
- Subsequently a 3-month prescription can be given.
- However drug refills are done monthly.

Follow-up and Monitoring of PrEP

- Asses adherence and provide ongoing adherence counselling and support
- Monitor for and manage side effects
- Exclude HIV infection
- Provide other prevention services including risk reduction counselling, condoms, STI screening etc
- Review indication for PrEP

Follow-up and Monitoring of PrEP

- Follow up after initiating Prep (Figure 4.1)
- HIV testing and managing suspected HIV infection during PrEP
  - Routine HIV testing during PrEP
  - Managing suspected acute sero-conversion illness
  - Managing confirmed HIV infection during PrEP
- Improving adherence to PrEP (table 4.1)

Follow-up and Monitoring of PrEP

- Assessing for medication side effects
  - Minor side effects (diarrhoea, nausea, decreased appetite)
  - Elevated creatinine
    - Where available serum creatinine should be estimated at baseline and annually thereafter
    - If the creatinine clearance is <50ml/min, discontinue PrEP immediately and counsel on other HIV preventive measures

Follow-up and Monitoring of PrEP

- Discontinuing PrEP
  - Can be discontinued at least 28 days from the last high risk exposure to HIV
- Restarting PrEP
- PrEP in special circumstances
  - Chronic HBV infection
  - Pregnancy and breastfeeding
  - PrEP use in discordance

NASCOP

HARAMBEE

Module 3

Clinic Case Management of PrEP

Case 1

Risk Assessment and Indications for PrEP

Tish, a 22 years old female, is requesting for PrEP after taking a HIV test (which is negative). She feels uncomfortable engaging in further conversation. She is the first born in her family and she takes care of her 3 siblings with the last born being HIV positive. She does causal domestic chores and lives in an informal settlement near the city.

Tish discloses that her parents died of HIV when she was 19 years. She is the first born in her family and she takes care of her 3 siblings with the last born being HIV positive.

She does causal domestic chores and lives in an informal settlement near the city. She further discloses that she has several ‘carefully chosen’ sexual partners and seldom uses condoms. She occasionally takes alcohol.

She says that she gets itchy/painful blisters on and off on her labia and pubic area. These break after a few days and heal after a week or so. During the flares, she applies ‘oil’ to soothe the pain.

- Does Tish have substantial ongoing risk of HIV infection?

If yes, what are they?

- Question 2: What are the indications for PrEP

- Question 3: Will Tish benefit from PrEP? Does she meet the criteria for PrEP as per the national guidelines?

- Question 4: What are the initiation clinical and laboratory assessments before providing PrEP?

- On physical examination, the only positive finding is ulceration in the external genitalia. Laboratory test results are all normal.

- Question 5: How will you manage Tish?

- Question 6: What drugs will you initiate Tish on for oral PrEP?

- The provider reschedules her next visit in 30 days. She returns to the clinic after 28 days.

- Question 7: What are the key issues for review during her current and subsequent appointment?

NASCOP

HARAMBEE

Case 2

Initiating PrEP in a HIV Discordant Couple

Stephen, a 42-year-old teacher, was diagnosed HIV positive 1 week ago. He has come back today to discuss baseline test results that were ordered during the last visit and for ART initiation.

He has also come with his wife, 38-year-old Maria, and their only child, 2-year-old Zeus, for family and partner testing. Both Maria and Zeus test HIV negative.

Stephen and Maria enquire about HIV prevention options available for them.

Question 1: What is your advice for this couple?

Question 2: What are the particular indications for PrEP in a serodiscordant couple?

The couple agrees that Stephen should start ART while Maria starts oral PrEP.

There is no significant finding in your clinical evaluation; and the laboratory results are pending.

Maria is willing to take and adhere to oral PrEP.

Maria takes PrEP for 6 months, while Stephen is adherent to ART and achieves full viral suppression.

Question 3: What do you do for Maria now?

One and a half years later, Stephen and Maria return to your clinic with results of laboratory tests done a week prior. Stephen’s viral load result was 5953 copies/ml and Maria has remained HIV negative.

Stephen’s adherence is unsatisfactory and Maria has not used PrEP for the last one year.

They would like to have another child and they would like your advice.

Question 4: What would you advise this couple?

Case 3

PrEP in Special Circumstances

- Millicent is a 22 year old hairdresser who visits your facility because her LMP was about 6 weeks ago and she suspects she is pregnant. She has several sexual partners who give her money to support with her shopping and rent. Her sexual partners prefer not to use condoms. She was treated for a sexually transmitted infection 3 months ago. She tests negative for HIV and positive for pregnancy. She asks for advice on HIV prevention.

- Question 1: Is Millicent at risk of HIV? Why?

- Question 2: Does Millicent qualify for PrEP?

- Millicent comes to your clinic 15 months after initiating oral PrEP. She has a 5-month old baby and has settled on only one sexual partner who has agreed ti use condoms. They have tested for HIV together and they are both HIV negative. She is considering stopping PrEP.

- Question 3: What is your advice for Millicent?

NASCOP

HARAMBEE

Case 4

A male with multiple male sexual partners

Clinic Scenario

Tame is a 48-year-old senior level regional (Eastern and Southern Africa) marketing executive in a multinational company in the city. He comes to the OPD complaining of painful inter-gluteal eruption. He is also vaguely ill with a sore throat and muscle pain.

From his search on the internet and information in a private ‘chat room’, he thinks the eruption is due to herpes. He asks you to to prescribe antivirals.

He is not comfortable with further discussions, especially ‘on the record’. Though he has a generous medical insurance cover, he prefers to pay for this consultation and medications ‘out of pocket’.

After counselling the provider finds out that Tame is a sexually active man who has sex with men. He has multiple sexual encounters with acquaintances, especially when he travels. He seldom uses condoms. He occasionally smokes marijuana and cocaine. He was treated for syphilis 5 years ago.

He weighs 76 kg and his height is 174 cm. His physical examination is notable for an elevated BP of 160/98 mmHg and inter-gluteal blisters, pustules and ulcers. He is not circumcised.

Tame's HIV test is negative.

Question 1: What are Tame's risks for HIV infection?

Question 2: Is Tame eligible for PrEP?

Question 3: Discuss a management plan for Tame

Tame’s HIV test after 4 weeks is negative; you decide to start him on PrEP

Question 4: How do you ascertain that Tame is ready to effectively use PrEP?

You start Tame on PrEP. He returns for his 6-month scheduled visit. His physical examination is normal with a weight of 74kg, and you perform urinalysis because of his NCD (hypertension).

You receive his laboratory test results, he has proteinuria (+) and his serum creatinine level is 150 micromol/L .

Question 5: Discuss how you will modify Tame's management in view of the new findings

Tame returns for his 9-month scheduled visit and his creatinine clearance has stabilized.

However, you establish that he has been using injectable narcotics for the last 2 months which he started during a southern African country visit. It is becoming increasingly difficult to get his shots forcing him to visit ‘dangerous places’ for his supply. He’s also drinking alcohol more than usual. His boss at work has warned him over erratic behavior and unmet deadlines. He has not taken most of his pills in the last month.

His HIV rapid test result is positive.

Question 6: What should be your next step for managing Tame?

NASCOP

HARAMBEE

Module 4

Commodities Management for PrEP Services in Kenya

Objectives of the session

By the end of this session, participants should be able to;

- Describe the ARVs Commodity & Information flow
- Briefly outline ART LMIS reporting tools
- Describe how to estimate drug quantities to order
- Outline dispensing & various dispensing points for oral PrEP medicines
- Describe pharmacovigilance for PrEP medicines

ART Commodity & Information flow (1)

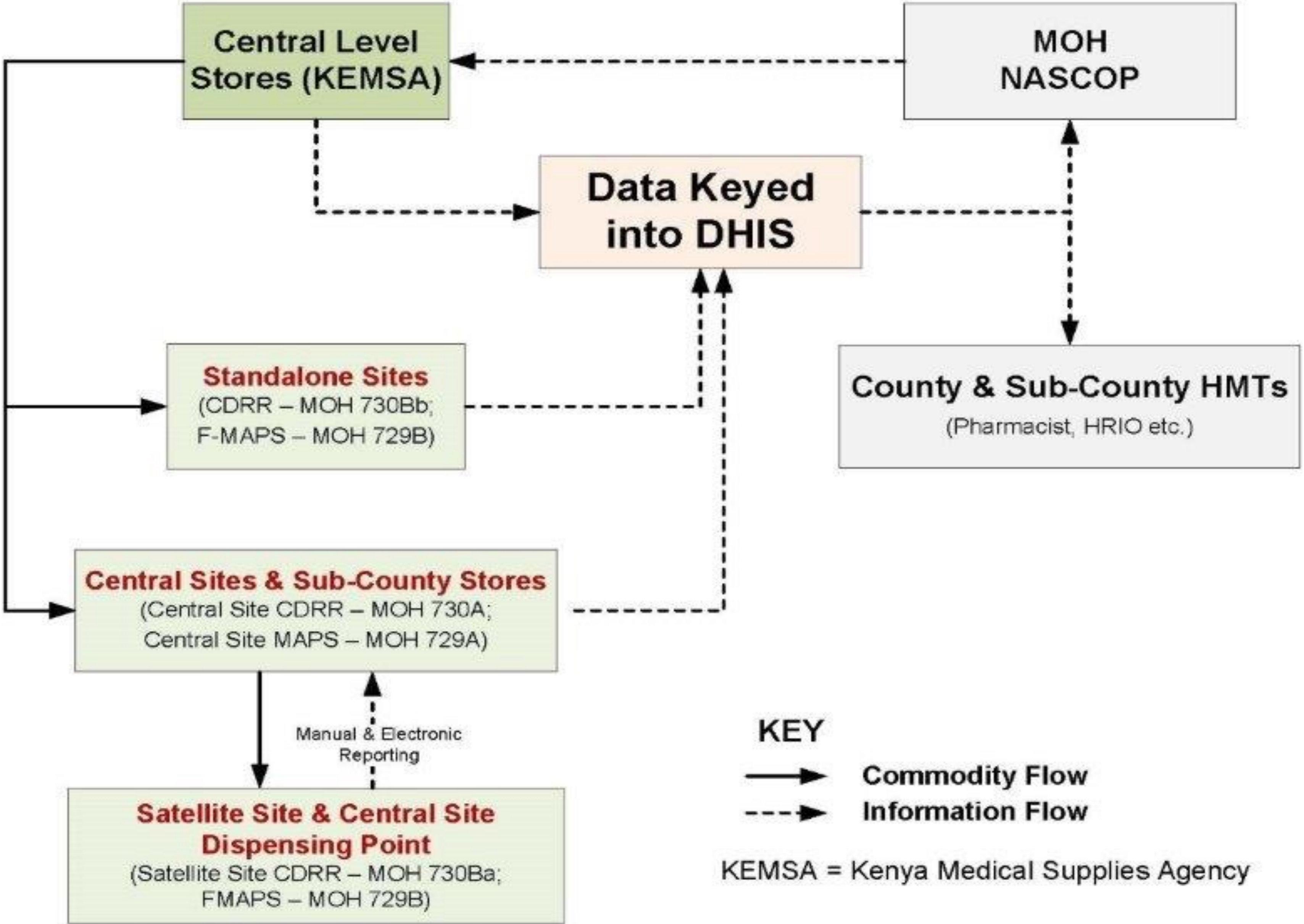

NASCOP

ART Commodity & Information flow (2)

- Sites should have capacity to accurately capture & report data (training/mentorship)
- Revised ART LMIS (2017) tools should be in place-(DAR, CDRR and MAPS)
- Electronic dispensing tools e.g. ADT and web ADT should be updated to capture current formulations & regimen codes
- Central sites to supply linked satellite sites (including DICES, research sites)
- Reporting timelines-by 5th & 10th of every month for satellites and ART ordering sites respectively

Revised ART LMIS tools

These tools include;

- Daily Activity Register for ARV & OI medicines - (MoH 367A)
- Facility CDRR for ARV & OI medicines - (MoH 730Ba for Satellites, MoH730Bb for Standalone sites)
- Central site / Sub-county store DRUGS for ARV & OI medicines - (MoH730A)
- Facility Monthly ARV Patient Summary (F-MAPS) - (MoH729B)
- Central site / Sub-county store Monthly ARV Patient Summary (D-MAPS, MoH729A)
  - ** CDRR = Consumption Data Report and Request*

NASCOP

Highlights of revised ART LMIS tools 2017 (1)….CDRR

Commodity Name	Unit of Issue / Pack size
Tenofovir/Lamivudine/Efavirenz (TDF/3TC/EFV) FDC (300/300/400mg) FDC Tablets	30s
Tenofovir/Lamivudine (TDF/3TC) FDC (300/300mg) Tablets	30s
Tenofovir/Emtricitabine (TDF/FTC) FDC (300/200mg) Tablets	30s

Highlights of revised ART LMIS tools 2017 (2)…MAPS

Regimen Code	ARV or OI Treatment Regimen	Number of Current Active Patients/Clients on this regimen at the end of this Reporting period
PRP1A	TDF + FTC (PrEP)	
PRP1B	TDF + 3TC (PrEP)	
PRP1C	TDF (PrEP)	

Determining quantity of PrEP ARVs to order

- Initial order
  - Estimate number of eligible clients
  - Multiply this number by 3 (or 2 for satellite sites), to estimate initial total number of packs to order
- Subsequent orders after initial supply
  - Pull system, using CDRR and MAPS
- Sites to adhere to reporting timelines for uninterrupted supplies

Dispensing PrEP

Ensure that;

- an effective form of medicine is delivered to the right patient in the prescribed dosage and quantity

- with clear instructions and in a package that maintains the potency of the medicine

Prescription Characteristics

A good ART prescription should have:

- Date
- Name of Patient
- Patient File/CCC No.
- Age of patient
- Weight of patient
- Height/BSA of patient (for some medicines e.g. AZT in children)
- Medicine, dose, frequency and duration
- Prescriber’s name and signature
- Address (name and telephone no.) of the facility

Pharmacovigilance for PrEP-Reporting

- Reporting of suspected ADRs-use the yellow form
- Reporting of suspected poor quality medicines-use Pink form
  - Electronic reporting via PPB online services is recommended
- Hypersensitivity reactions to PrEP, if any-use patient Alert Card (White Card).

NASCOP

HARAMBEE

Case 1

Dispensing Oral PrEP

Halima, a female sex worker, has received information about PrEP from her friend who is a peer educator.

She considers herself at risk and decides to find more about PrEP services at her nearest facility. On arrival, she is informed about the combination prevention package that includes regular use of condoms and also use of oral PrEP as an additional prevention method.

She agrees to a screening procedure and is considered eligible for oral PrEP. She is immediately initiated on a one-month prescription of TDF/FTC issues to her

Question: Describe 10 components of medication use counselling that should ne considered by the health care worker tasked with dispensing TDF/FTC to Halima at the facility’s pharmacy.

You have 10 minutes for this exercise.

Case 2
Completing ART LMIS tools

NASCOP

Question 1:

- Fill in the daily activity register for ARV and OI drugs provided, appropriately capturing all the above activities in the month of May 2017

Question 2

- Using the above information, fill in the facility Consumption Data Report and Request (F-CDRR) as at 31st May 2017, indicating any quantities the facility may need from the central site.

Question 3:

- Fill in the facility Monthly ARV patient summary (F-MAPS) as at 31st May 2017

NASCOP

HARAMBEE

Module 5

PrEP Data Collection and Reporting Tools

November 2017

Presentation Outline

- Learning Objectives
- Introduction to PrEP
- Primary data sources for PrEP
- Reporting PrEP

Primary Data Sources for PrEP

- PrEP Clinical Encounter form
- PrEP Register
- PrEP Daily Activity Register
- PrEP Summary tool/ MoH731b

PrEP Clinical Encounter Record

Introduction of PrEP Clinical Encounter record

- **Purpose:** Captures all the details of the PrEP client in the initial visit and subsequent follow up
- Facilities with EMR will use the electronic encounter record
- It has 3 broad sections:
    - Client Baseline information
    - Clinical follow up section
    - Monthly refill section

Introduction of PrEP Clinical Encounter record

Client Baseline information

- Client profile
- Entry point and Transfer status
- Baseline assessment
    - Behavioral risk assessment
    - Medical assessment and fertility intentions
- PrEP initiation
- Next appointment date

Clinical follow up section

- Medical assessment and fertility intentions
- Behavioral risks assessment
- Follow up laboratory investigations
- PrEP dispensed during the visit
- Date for next appointment

NASCOP

Introduction of PrEP Clinical Encounter record

Monthly refill section

Date of Refill	Behavior risk assessment	Adherence counselling	Continue/Discontinue PrEP	Next appointment date	Remarks
Month 2					
4					
5					
7					
8					
10					

Overview of the Clinical Encounter record

Who fills: Filled by the clinician offering PrEP services to the client

When filled: Completed in the course of service delivery

Location: One designated PrEP room

PrEP register

Introduction to PrEP register

- The PrEP Register should be filled for every client on PrEP and follow up
- It captures clients information: bio data, reasons for PrEP eligibility, STI screening , adherence and PrEP status

Overview of the PrEP Register

Purpose : It is a longitudinal register that captures PrEP clients' information

Location: Placed in the PrEP room

When completed: In the course of PrEP service delivery

Who: service provider, Health Records officer or the data clerk who is assigned the responsibility of updating PrEP records at the facility

PrEP Daily Activity Register

Overview of the PrEP Daily Activity Register

Purpose : It is a summary of reportable data elements that demand immediate collection upon provision of PrEP service

It will act as the source document for the PrEP summary tool

Location: Placed in the PrEP room

When completed: Immediately after PrEP service is provided

Who: service provider, Health Records officer or the data clerk who is assigned the responsibility of updating PrEP records at the facility

Key highlights in PrEP DAR

- To include all clients seeking PrEP services
  - New clients on PrEP
  - clients visiting for prescribed follow up visits (PrEP Register)
  - visiting for the monthly refills (Monthly refill section of the encounter record)
- Tick (√) as appropriate for these sections of the register:
  - key populations
  - started on PrEP
  - restarting PrEP
  - diagnosed with STI
  - discontinued PrEP
  - eligible for PrEP
  - refills on PrEP
  - retested HIV positive while on PrEP

PrEP Summary Reporting Tool

Overview of the PrEP summary tool

Purpose: Collects monthly summaries of PrEP reportable data elements – will populate the MOH 731plus

Location: Each facility will have one summary tool which aggregates data at the HRIO office

When completed: At the end of the reporting month

Who: Completed by the service provider ,HRIO or data clerk as per facilities procedures

Reportable PrEP Indicators

- The PrEP data elements have been disaggregated by Sex and Age
- The age disaggregation include: 15-19,20-24, 25-29 and 30+ years
- PrEP reportable indicators include:
  - Number Eligible for PrEP
  - Number initiated (New) on PrEP
  - Number continuing (Refills) on PrEP
  - Number Restarting (Restart) PrEP
  - Number currently on PrEP (New + Refill+ Restart)
  - Number tested HIV positive while on PrEP
  - Number diagnosed with STI
  - Number discontinued PrEP

NASCOP

Data use

- Can be used at Health Facility level, county and national-level analysis:
    - Number of clients who seroconvert; the number with STIs and those who discontinue using PrEP
    - HIV risk patterns for different population types within the counties
    - Plan interventions to improve PrEP uptake
- Ordering PrEP commodities (PrEP drugs and Lab DBS bundles)
- Linkage for PrEP clients who sero-convert to CCC for Care and Treatment

Case 1

PrEP Clinical Encounter Form and Register

- Question 1: Using the information provided in the case scenario, complete the PrEP Clinical Encounter Form

- Question 2: Transfer the information you have completed to the PrEP longitudinal register

Case 2

PrEP Monthly Summary Tool

- Question 1: Using the prepopulated register provided, complete the PrEP monthly summary tool
