# Supplementary material for: Using an on-site modular training approach to amplify prep service delivery in public health facilities in Kenya
Source: PLOS Glob Public Health. 2022 Mar 10;2(3):e0000092. doi: 10.1371/journal.pgph.0000092 (PMC10021257; doi:10.1371/journal.pgph.0000092)
Supplement: S2 Text — (PDF) [file pgph.0000092.s002.pdf]

Ministry of Health

Healthcare Worker Orientation Package on the Pre-exposure Prophylaxis

Participant's Manual

November 2017

Table of Contents

Introduction 3
i. Goal of the Orientation Package 3
ii. Learning Objectives 3
iii. Target Audience 3
iv. Training Resource Materials 4
v. Methodology 4
Unit 1: Clinical Case Management of PrEP 5
Unit 2: Commodity Management for Oral PrEP 17
Unit 3: Monitoring and Evaluation of PrEP Services 22

Introduction

i. Goal of the Orientation Package

To sensitize healthcare workers on the Pre-exposure Prophylaxis Toolkit so they can plan, implement, and evaluate differentiated HIV care in Kenya.

ii. Learning Objectives

By the end of the training, participants should be able to:

- Define pre-exposure prophylaxis (PrEP) for the prevention of HIV infection
- Outline the evidence for PrEP as part of a package of combination HIV prevention
- Perform assessment for risk of HIV infection and understand the indications and contraindications for PrEP
- Prepare, initiate, monitor and discontinue PrEP
- Understand data and reporting for PrEP service delivery and quality improvement

iii. Target Audience

This package is targeted towards healthcare workers, health facility leadership, and County and Sub-County Health Management Teams.

iv. Training Resource Materials

The training package for HCWs includes:

- PowerPoint slides to provide an overview of Pre-exposure Prophylaxis
- Participant's Workbook, containing:
  - Cases and questions
  - Role plays

This training package should be used in conjunction with the following related resource material:

- Pre-exposure Prophylaxis Toolkit
- Guidelines on Use of Antiretroviral Drugs for Treating and Preventing HIV Infection in Kenya (2016)

All these materials should be available to use during the training, including additional copies of the reporting tools to use during the practical exercises.

v. Methodology

This orientation package is case-based (with just a brief introductory PowerPoint presentation) and thus intended to be interactive through facility-based case discussions, case studies, role-plays, and practical exercises. Participants should receive the Participant's Workbook, which contains cases and questions. Facilitators will lead case discussions from the orientation package. The material is intended to take two days to cover completely. The material can also be delivered through several facility-based Continuous Medical Education sessions.

Unit 1: Clinical Case Management of PrEP

Unit Objectives

By the end of the unit participants should be able to:

- Define pre-exposure prophylaxis (PrEP) for the prevention of HIV infection
- Outline the evidence for PrEP as part of a package of combination HIV prevention
- Perform assessment for risk of HIV infection and understand the indications and contraindications for PrEP
- Prepare, initiate, monitor and discontinue PrEP

Case 1: Risk Assessment and indications for PrEP (Role Play)

Tish, a 22-year-old female, is requesting for PrEP after taking a HIV test (which is negative). She is the first born in her family and she takes care of her 3 siblings with the last born being HIV positive. She does causal domestic chores and lives in an informal settlement near the city. She feels uncomfortable engaging in further conversation.

Role Play 1: Start a counselling session. What approach would you use to help the client provide additional information necessary for you to prescribe PrEP?

Question 1: Does Tish have substantial ongoing risk of HIV infection?

Refer to ART Guidelines (2016) Table 11.2 Risk behavior assessment and the PreP Toolkit

Question 2: What are the indications for PrEP?

Refer to ART Guidelines (2016) Page 129 Indications and criteria for PrEP; PrEP Toolkit – Indications for PrEP

Question 3: Will Tish benefit from PrEP? Does she meet the criteria for PrEP as per the national guidelines?

Refer to ART Guidelines (2016) Page 130 Indications and criteria for PrEP; PrEP Toolkit

The provider decides that Tish will benefit from PrEP.

Question 4: Describe the initiation clinical and laboratory assessment before providing PrEP.

Refer to ART Guidelines (2016) Page 131; and PrEP; Toolkit Tables 3.1 and 3.2

On physical examination, the only positive finding is ulceration in the external genitalia. Laboratory tests results are all normal.

Question 5: How will you manage Tish?

Refer to PrEP Toolkit Table 3.2: Managing Clinical and Laboratory Results on Initial and Follow-up Assessment on management of positive STI screen

Role Play 2: Ask another pair to start a counselling session to prepare Tish for PrEP.

Refer to ART Guidelines (2016) Page 133; Toolkit Table 3.3

Question 6: What drugs will you initiate for Tish for oral PrEP?

Refer to ART Guidelines (2016) Table 11.1Page; Toolkit Table 3.6.

The provider reschedules her next visit in 30 days. She returns to the clinic after 28 days.

Question 7: What are the key issues for review during her current and subsequent appointment?

Refer to ART Guideline (2016) Tables 11.3 and 11.4; Toolkit Section 4: Follow-up and Monitoring of Pre-Exposure Prophylaxis and Figure 1.1

ase 2: Initiating PrEP in a Discordant Couple

tephen, a 42-year-old teacher, was diagnosed HIV positive 1 week ago. He has come bacl oday to discuss baseline test results that were ordered during the last visit and for ART nitiation. He has also come with his wife, 38-year-old Maria, and their only child, 2-year-olc eus, for family and partner testing. Both Maria and Zeus test HIV negative. Stephen anc Iaria enquire about HIV prevention options available for them.

uestion 1: What is your advice for this couple?

Refer to the ART Guidelines (2016) Section 11: Oral pre-exposure prophylaxis

he couple agrees that Stephen should start ART while Maria starts oral PrEP. **Question**

: What are the particular indications for PrEP in a sero-discordant couple? *Refer to the*

RT Guidelines (2016) Table 11.3: Summary of initial and follow-up assessment

There is no significant finding in your clinical evaluation; and the laboratory results are pending. Maria is willing to take and adhere to oral PrEP. Maria takes PrEP for 6 months, while Stephen is adherent to ART and achieves full viral suppression.

Question 3: What do you do for Maria now?

Refer to the ART Guidelines (2016) and Toolkit pg 17

One and a half years later, Stephen and Maria return to your clinic with results of laboratory tests done a week prior. Stephen's viral load result was 5,953 copies/ml and Maria has remained HIV negative. Stephen's adherence is unsatisfactory and Maria has not used PrEP for the last one year. They would like to have another child and they are seeking your advice.

Question 4: What would you advise this couple?

Refer to the ART Guidelines (2016) Table 4.8: Pre-conception counselling messages and services for PLHIV

Case 3: PrEP in special circumstances

Millicent is a 22-year-old hairdresser who visits your facility because her LMP was about 6 weeks ago and she suspects that she is pregnant. She has several sexual partners who give her money to support with her shopping and rent. Her sexual partners prefer not to use condoms. She was treated for a sexually transmitted infection 3 months ago. She tests negative for HIV and positive for pregnancy. She asks for advice on HIV prevention.

Question 1: Is Millicent at risk of HIV? Why?

Question 2: Does Millicent qualify for PrEP?

Refer to Toolkit pg 17: Pregnancy/breastfeeding

Millicent comes to your clinic 15 months after initiating oral PrEP. She has a 5-month-old baby, and has settled on only one sexual partner who has agreed to use condoms. They have tested for HIV together and they are both HIV negative. She is considering stopping PrEP.

Question 3: What is your advice for Millicent?

Refer to the ART Guidelines (2016) Section 11.6: Criteria for discontinuing PrEP

Case 4: A male with multiple male sexual partners

Tame is a 48-year-old senior level regional (Eastern and Sothern Africa) marketing executive in a multinational company in the city. He comes to the OPD complaining of painful inter-gluteal eruption. He is also vaguely ill with a sore throat and muscle pain. From his search on the internet and information in a private 'chat room', he thinks the eruption is due to herpes. He asks you to prescribe antivirals. He is not comfortable with further discussions, especially 'on record'. Though he has a generous medical insurance cover, he prefers to pay for this consultation and medications 'out-of-pocket'.

After counselling the provider finds out that Tame is a sexually active man who has sex with men. He has multiple sexual encounters with acquaintances, especially when he travels. He seldom uses condoms. He occasionally smokes marijuana and cocaine. He was treated for syphilis 5 years ago. He weighs 76 kg and his height is 174 cm. His physical examination is notable for an elevated BP of 160/98mmHg and inter-gluteal blisters, pustules and ulcers. He is not circumcised. Tame's HIV test is negative.

Question 1: What are Tames Risks for HIV infection?

Question 2: Is Tame eligible for PrEP?

Refer to ART Guideline (2016) Page 132 Section 11.5 contraindication to oral PrEP; Toolkit table 2.1

Question 3: Discuss a management plan for Tame.

Tame's HIV test after 4 weeks is negative, you decide to start him on PrEP.

Question 4: How do you ascertain that Tame is ready to effectively use PrEP?

Refer to ART Guideline (2016) Tables 11.5 and 11.6; Toolkit tables 3.4 and 3.5: Pre-initiation education and Pre-initiation assessment checklist

You start Tame on PrEP. He returns for his scheduled 6-month visit. His physical examination is normal with a weight of 74kg, and you perform urinalysis because of his non-communicable disease (Hypertension). You receive his laboratory test results, he has proteinuria (+) and his serum creatinine level is 150 µmol/L

Question 5: Discuss how you will modify Tame's management in view of the new findings.

Tame returns for his 9 month scheduled visit and his creatinine clearance has stabilized. However, you establish that he has been using injectable narcotics for the last 2 months which he started during a visit to a Southern African country. It is increasingly difficulty to get his shots forcing him to visit 'dangerous places' for his supply. He is also drinking more alcohol than 'usual'. His boss at work has warned him over his erratic behavior and unmet deadlines. He has not taken most of his pills in the last month. His HIV rapid test result is positive

Question 6: What should be your next step for managing Tame?

Refer to ART Guideline (2016) page 133 section 11.6; Toolkit: Discontinuing PrEP

Unit 2: Commodity Management for Oral PrEP

Unit Objectives

By the end of this module, participants will be able to:

- Describe key components of medication use counselling with regard to dispensing PrEP
- Learn how to complete the Daily Activity Register for ARVs and OI Drugs
- Prepare a Facility Consumption Data Report & Request (F-CDRR) for ARV & OI drugs
- Prepare a Facility Monthly ARV patient summary(F-MAPs)

Case 1: Dispensing Oral PrEP

Halima, a female sex worker, has received information about pre-exposure prophylaxis (PrEP) from her friend who is a peer educator. She considers herself at risk and decides to find more about PrEP services at her nearest health facility. On arrival, she is informed about the combination prevention package that includes regular use of condoms and also use of oral PrEP as an additional prevention method. She agrees to a screening procedure and is considered eligible for oral PrEP. She is immediately initiated and a one-month prescription of TDF/FTC issued to her.

Question 1

Describe 10 components of medication use counselling that should be considered by the health care worker (HCW) tasked with dispensing TDF/FTC to Halima at the facility's pharmacy. You have 10 minutes.

Expected Responses

Case 2: Completing ART LMIS Tools

JiPrep Health Centre, an ART satellite site with MFL Code 99999, is a government health facility in Makadara Sub County, Nairobi County. It is now in its fourth month of providing oral PrEP services to eligible clients. On 30th April 2017, the facility had the following stocks of drugs at the pharmacy.

No	Drug Name and strength	Pack size	Stock on Hand (packs)
1	Tenofovir/Emtricitabine FDC tabs 300/200 mg	30	10
2	Nevirapine 10mg/ml	100ml bottle	5
3	Tenofovir/Lamivudine/Efavirenz FDC 300/300/600mg	30	15
4	Zidovudine/Lamivudine FDC 300/150mg	60	6
5	Efavirenz 600mg	30	5
6	Zidovudine 10mg/ml	240 ml bottle	4

The following transactions took place in the month of May, 2017:

1. On 2nd May 2017, Mr. Otiato, Pt/no. a234, who is in discordant relationship, visited the clinic on his third appointment. The pharmaceutical technologist dispensed a one-month dose of TDF/FTC 300/200mg FDC to her.
2. On 4th May 2017, Ms. Moreu, Pt/no. a300, a client who reported to have multiple sexual partners and unable to negotiate use of condoms with them visited the facility. After screening, she was found to be eligible for oral PrEP and a one-month dose of TDF/FTC 300/200mg was dispensed to her.
3. On 6th May 2017, the pharmaceutical technologist in-charge received the following drugs from the central site: Lopinavir/ritonavir 80/20 mg/ml, 60ml bottle, 11 bottles; Abacavir/Lamivudine 120/60 mg FDC, 30s, 40 packs and TDF/FTC 300/200mg tabs 9 packs.
4. On 16th May 2017, Brian, a client who usually engages in unprotected sex with other men whose HIV status he doubts, visited the facility. Upon testing, he was found to be HIV negative and eligible for oral PrEP. A one-month dose of TDF/FTC 300/200 mg FDC was dispensed to him as oral PrEP.

5. On 29th May 2017, Shauri Moyo Health Centre borrowed four packs of TDF/FTC, five packs of TDF/3TC/EFV and two bottles of Zidovudine syrup.

Assuming that these were the only transactions that took place at the facility in the month of May 2017, complete the following exercise in the allocated groups. You have 20 minutes.

Question 1: Fill in the Daily Activity Register for ARV and OI drugs provided, appropriately capturing all the above activities in the month of May 2017.

MONTH: YEAR:

Name of this Dispensing area: ____________________

(e.g. Pharmacy, ANC, MCH, CCC, etc)

ARV FORMULATIONS

Date	Client No. (IP, OP, MCH Clinic,	Client Name (Optional)	Age [C for Child (<15 Yrs) or A for	New(N) /Revisit(R)	Dispensing Officer (Name / Initials)	ARV Regimen Code	OI and IPT Regimen Code	Abacavir (ABC) 300mg Tablets	Abacavir/Lamivudine (ABC/3TC) 120mg/60mg FDC Tablets	Efavirenz (EFV) 600mg Tablets	Lopinavir/ritonavir (LPV/r) liquid 80/20mg/ml (60ml Bottles)	Nevirapine (NVP) 200mg Tablets	Nevirapine (NVP) Susp 10mg/ml	Tenofovir/Emtricitabine (TDF/FTC) FDC (300/200mg) Tablets	Tenofovir/Lamivudine (TDF/3TC) FDC (300/300mg) Tablets	Tenofovir/Lamivudine/Dolutegravir (TDF/3TC/DTG) FDC (300/300/50mg) FDC Tablets	Tenofovir/Lamivudine/Efavirenz (TDF/3TC/EFV) FDC (300/300/400mg) FDC Tablets	Tenofovir/Lamivudine/Efavirenz (TDF/3TC/EFV) FDC (300/300/600mg) FDC Tablets	Zidovudine (AZT) 300mg Tablets	Zidovudine (AZT) liquid 10mg/ml	Zidovudine/Lamivudine (AZT/3TC) FDC (300/150mg) Tablets
Balance B/F (In Units) (A)																					
Quantity Received (In Units) (B)																					
Stock on Hand (Balance B/F plus Quantity Received) (In Units) (C = A + B)																					
Total Quantity Dispensed (In Units) (D)																					
Losses (In Units) (E)																					
Balance C/F (In Units) (F)																					

PAGE TOTALS:

Total number of Patients on Medicines for Opportunistic Infections (OIs):-

ADULTS	New	Revisit	CHILDREN	New	Revisit	Hepatitis B Patients who are HIV-ve	New	Revisit
OI1A			OI1C			HPB1A		
OI2A			OI2C			HPB1B		
OI4AN			OI4CN					
OI5A			OI5C					

Total number of Patients / Clients per ARV Regimen:-

ADULT ART REGIMENS

	New		New	Revisit		New	Revisit		New	Revisit	
AF1A		AS5B			CF4A			CS1A			CT1D
AF1B		AS6X			CF4B			CS1B			CT1E
AF1D		AT1D			CF4C			CS2A			CT1F
AF2A		AT1E			CF4D			CS2C			CT1G
AF2B		AT1F			CF5X			CS4X			CT2B

Refer to front pages of this DAR book for the Official List of the Regimen codes. Do NOT create any other Regimen codes. NB: If none of the codes applies, use the "Other" regimen codes provided, e.g. AF5X for ALL OTHER 1st line Adult regimens, PM1X for ALL OTHER PMTCT regimens for Women, etc.

Question 2: Using the above information, fill in the Facility Consumption Data Report and Request (F-CDRR) as at 31st May, 2017, indicating any quantities the facility may need from the central site.

MINISTRY OF HEALTH

FACILITY CONSUMPTION DATA REPORT and REQUEST (F-CDRR) for ANTIRETROVIRAL and OPPORTUNISTIC INFECTION MEDICINES

MoH 730Ba

Facility Name: Jiprep HC ______________________ Facility (MFL) code: ______________________

County: Nairobi ______________________ Sub-County: Makadara

Period of Reporting: May 2017 Beginning: ____________ (Day/Month/Year) Ending: ____________ (Day/Month/Year)

Drug Name	Unit pack size	Beginning Balance	Total Quantity Received this month	Total Quantity Dispensed this month	Losses & Wastage	Positive Adjustments	Negative Adjustments	End of Month Physical Stock Count (For CS Dispensing point, please exclude the Central site dispesnsing point stocks)	Commodities expiring in less than 6 months: Quantity	Commodities expiring in less than 6 months: Earliest Expiry date mm/yyyy	Days out of stock this Month	Quantity Requested for RE-SUPPLY
		A	B	C	D	E	F	G			H	I
ARVs												
Adult preparations												
Efavirenz (EFV) 600mg Tablets	30s											
Tenofovir/Emtricitabine (TDF/FTC) FDC (300/200mg) Tablets	30s											
Tenofovir/Lamivudine/Efavirenz (TDF/3TC/EFV) FDC (300/300/600mg) FDC Tablets	30s											
Zidovudine/Lamivudine (AZT/3TC) FDC (300/150mg) Tablets	60s											
Paediatric preparations												
Abacavir/Lamivudine (ABC/3TC) 120mg/60mg FDC Tablets	30s											
Lopinavir/ritonavir (LPV/r) liquid 80/20mg/ml (60ml Bottles)	60ml bottle											
Nevirapine (NVP) Susp 10mg/ml (100 ml Bottles)	100ml bottle											
Zidovudine (AZT) liquid 10mg/ml (240ml Bottles)	240ml bottle											

Comments (Explain ALL Losses and Adjustments): Note - If space insufficient, attach additonal sheet(s) as required

ART Data collection and Reporting Tools

To be requested only when your Tools are nearly finished. Indicate quantity in table below for each tool type.

Name of Data Collection or Reporting tool	DAR (MoH 367A) ARVs & OIs 50 page	DAR (MoH 367A) ARVs & OIs 300 page	F-CDRR (MoH730B)	F-MAPS (MoH729B)	D-CDRR** (MoH730A)	D-MAPS** (MoH729A)
Quantity requested						

**Applies only to a site being upgraded to Central site status

Report prepared by: ______________________ Signature: ______________________ Designation: ______________________

Name of Reporting officer

Contact Telephone: ______________________ Date: ______________________

Report approved by: ______________________ Signature: ______________________ Designation: ______________________

Name of Approving officer

Contact Telephone: ______________________ Date: ______________________

For Satellite sites and Central Site Dispensing points

MoH 730Ba ver May 2016

3. Fill in the Facility Monthly ARV patient summary (F-MAPs) as at 31st May, 2017.

MONTH: YEAR:

Name of this Dispensing area: ____________________

(e.g. Pharmacy, ANC, MCH, CCC, etc)

Date	Client No. (IP, OP, MCH Clinic.	Client Name (Optional)	Age [C for Child (<15 Yrs) or A for	New(N) /Revisit(R)	Dispensing Officer (Name / Initials)	ARV Regimen Code	OI and IPT Regimen Code	Abacavir (ABC) 300mg Tablets	Abacavir/Lamivudine (ABC/3TC) 120mg/60mg FDC Tablets	Efavirenz (EFV) 600mg Tablets	Lopinavir/ritonavir (LPV/r) liquid 80/20mg/ml (60ml Bottles)	Nevirapine (NVP) 200mg Tablets	Nevirapine (NVP) Susp 10mg/ml	Tenofovir/Emtricitabine (TDF/FTC) FDC (300/200mg) Tablets	Tenofovir/Lamivudine (TDF/3TC) FDC (300/300mg) Tablets	Tenofovir/Lamivudine/Dolutegravir (TDF/3TC/DTG) FDC (300/300/50mg) FDC Tablets	Tenofovir/Lamivudine/Efavirenz (TDF/3TC/EFV) FDC (300/300/400mg) FDC Tablets	Tenofovir/Lamivudine/Efavirenz (TDF/3TC/EFV) FDC (300/300/600mg) FDC Tablets	Zidovudine (AZT) 300mg Tablets	Zidovudine (AZT) liquid 10mg/ml	Zidovudine/Lamivudine (AZT/3TC) FDC (300/150mg) Tablets
Balance B/F (In Units) (A)																					
Quantity Received (In Units) (B)																					
Stock on Hand (Balance B/F plus Quantity Received) (In Units) (C = A + B)																					
Total Quantity Dispensed (In Units) (D)																					
Losses (In Units) (E)																					
Balance C/F (In Units) (F)																					

PAGE TOTALS:

Total number of Patients on Medicines for Opportunistic Infections (OIs):-

ADULTS	New	Revisit	CHILDREN	New	Revisit	Hepatitis B Patients who are HIV-ve	New	Revisit
OI1A			OI1C			HPB1A		
OI2A			OI2C			HPB1B		
OI4AN			OI4CN					
OI5A			OI5C					

Total number of Patients / Clients per ARV Regimen:-

ADULT ART REGIMENS

	New		New	Revisit		New	Revisit		New	Revisit	
AF1A		AS5B			CF4A			CS1A			CT1D
AF1B		AS6X			CF4B			CS1B			CT1E
AF1D		AT1D			CF4C			CS2A			CT1F
AF2A		AT1E			CF4D			CS2C			CT1G
AF2B		AT1F			CF5X			CS4X			CT2B

Refer to front pages of this DAR book for the Official List of the Regimen codes. Do NOT create any other Regimen codes. NB: If none of the codes applies, use the "Other" regimen codes provided, e.g. AF5X for ALL OTHER 1st line Adult regimens, PM1X for ALL OTHER PMTCT regimens for Women, etc.

Unit 3: Monitoring and Evaluation of PrEP Services

Unit Objectives

By the end of this unit you should be able to:

- Document PrEP service provision at the facility and community levels
- Accurately extract and report service data from the various tools onto the PrEP Summary Reporting Form

Case Scenario 1: PrEP Clinical Encounter Form and Register

Mary Makhoha Chengo, 23 years of age from Kisauni area in Mombasa presented herself at OPD in the Coast General Hospital (MFL: 11289) on 17th May, 2017 as the 10th client, with interest to start PrEP. She is a sex worker and uses condoms during sex with commercial clients but not with her 'stable' partner of unknown HIV status. She had a negative HIV test 6 months ago, and wants to avoid HIV infection as she would like to have a baby in the coming year. She is using injectable hormonal contraceptive. She was referred to HTS room for counselling and testing. Her HIV results were negative. She was counselled on HIV prevention and PrEP adherence. The counsellor referred Mary to the clinician where she was taken her weight 64kg, BP 112/90mmHg and Height 182cm, no signs or symptoms of STI neither did she have any comorbidities. Since she was willing to take PrEP the clinician initiated her on PrEP (TDF-FTC) for one month and samples for creatinine, Hepatitis B & C tests were taken. She was advised to come with her stable partner for HTS in her next visit. Mary had follow up visits as follows and was offered with services in each follow up visit as follows:

On 16thJune 2017, Mary came back for clinical checkup and refill. Her weight was 60kg, BP 118/80mmHg and had no signs or symptoms of STI. She had no adverse drug reactions. Her HIV test results came out as non-reactive. She was given her creatinine which was 85ml/min. Her Hepatitis B&C tests were also negative. Adherence assessment was done and found to be good. Before she was issued with her PrEP refill she was counseled on adherence to the drugs and she was given two months TCA. In this visit her risk assessment was done and it was still having multiple sex partners, she was having transactional sex and inconsistent use of condoms. She was issued with condoms and advised to use them consistently.

16th Aug 2017.

Her HIV test was non-reactive but she turned out to have Syphilis and was given treatment for the same. Mary was still found to be on risk of being infected with HIV because her partner is of unknown status and she engages in transactional sex. In addition, she doesn't use condoms consistently. The service provider counseled Mary on adherence after realizing her adherence was fair because of forgetting to take her pills sometimes. Mary's weight during this visit was 58Kgs. She was encouraged to use and was issued with condoms. At this visit she is still taking PrEP.

15th Nov 2017.

During this visit Mary was accompanied by her 'stable' partner and they were both tested for HIV. The test results for the couple was negative concordant. She didn't show any sign of STI. Lab investigations confirm that she had been cured of Syphilis. Her weight during this visit was 60Kg. From adherence counseling and assessment, her adherence for PrEP was good. She was issued with condoms and agreed to continue on PrEP. From her risk assessment she was now using condoms consistently but had many sexual partners and transactional sex.

Exercise 1: Using the information provided in the case scenario complete the PrEP Clinical Encounter Form

Exercise 2: Transfer the information you have completed to the PrEP Longitudinal Register

Case Scenario 2: PrEP Monthly Summary Tool

Using the prepopulated PrEP register provided, complete the PrEP monthly summary tool.

Exercise 1: Use your completed PrEP monthly summary tool to respond to the questions below:

1. What is the total number of clients assessed for HIV risk?
2. What is the total number of clients eligible for PrEP?
3. What is the number of clients newly initiated on PrEP?
4. What is the number of continuing clients on PrEP?
5. What is the number of clients restarting PrEP?
6. What is the number of clients currently on PrEP?
7. What is the number of clients tested HIV positive while on PrEP?
8. What is the number of PrEP clients diagnosed with an STI?
9. What is the number of clients who discontinued from using PrEP?

NATIONAL AIDS & STI PROGRAMME

PrEP Summary Reporting Tool

Site Name/Facility:________________________ MFL-Code: ________________________

Sub-County: ______________________ County: __________________________

Reporting Month: _________________ Year________________

1. Number Eligible for PrEP	
1.1 Males 15 - 19 Years	
1.2 Females 15 - 19 Years	
1.3 Males 20 - 24 Years	
1.4 Females 20 - 24 Years	
1.5 Males 25 - 29 Years	
1.6 Females 25 – 29 Years	
1.7 Males 30 Years and older	
1.8 Females 30 Years and older	
**Total**	

2. Number initiated (New) on PrEP	
2.1 Males 15 - 19 Years	
2.2 Females 15 - 19 Years	
2.3 Males 20 - 24 Years	
2.4 Females 20 - 24 Years	
2.5 Males 25 - 29 Years	
2.6 Females 25 - 29Years	
2.7 Males 30 Years and older	
2.8 Females 30 Years and older	
**Total**	

3. Number continuing (Refills) on PrEP	
3.1 Males 15 - 19 Years	
3.2 Females 15 - 19 Years	
3.3 Males 20 - 24 Years	
3.4 Females 20 - 24 Years	
3.5 Males 25 - 29 Years	
3.6 Females 25 - 29 Years	
3.7 Males 30 Years and older	
3.8 Females 30 Years and older	
**Total**	

4. Number Restarting PrEP	
4.1 Males 15 - 19 Years	
4.2 Females 15 - 19 Years	
4.3 Males 20 - 24 Years	
4.4 Females 20 - 24 Years	
4.5 Males 25 - 29 Years	
4.6 Females 25 - 29 Years	
4.7 Males 30 Years and older	
4.8 Females 30 Years and older	
**Total**	

5. Number currently on PrEP (New + Refill+ Restart)	
5.1 Males 15 - 19 Years	
5.2 Females 15 - 19 Years	
5.3 Males 20 - 24 Years	
5.4 Females 20 - 24 Years	
5.5 Males 25 - 29 Years	
5.6 Females 25 - 29 Years	
5.7 Males 30 Years and older	
5.8 Females 30 Years and older	
**Total**	

6. Number tested HIV positive while on PrEP	
6.1 Males 15 - 19 Years	
6.2 Females 15 - 19 Years	
6.3 Males 20 - 24 Years	
6.4 Females 20 - 24 Years	
6.5 Males 25 - 29 Years	
6.6 Females 25 - 29 Years	
6.7 Males 30 Years and older	
6.8 Females 30 Years and older	
**Total**	

7. Number diagnosed with STI	
7.1 Males 15 - 19 Years	
7.2 Females 15 - 19 Years	
7.3 Males 20 - 24 Years	
7.4 Females 20 - 24 Years	
7.5 Males 25 - 29 Years	
7.6 Females 25 - 29 Years	
7.7 Males 30 Years and older	
7.8 Females 30 Years and older	
**Total**	

8. Number discontinued PrEP	
8.1 Males 15 - 19 Years	
8.2 Females 15 - 19 Years	
8.3 Males 20 - 24 Years	
8.4 Females 20 - 24 Years	
8.5 Males 25 - 29 Years	
8.6 Females 25 - 29 Years	
8.7 Males 30 Years and older	
8.8 Females 30 Years and older	
**Total**	

File no: Version 3.0 (29 August 2017 - WORKING)

Clinical Encounter Record: Oral Pre-Exposure Prophylaxis (PrEP)

Name of facility: ____________ Delivery Point: ____________ Tier: ______ MFL code: __ __ __ __ __
County: ____________ Sub county: ____________ Ward: ____________

A. Client Profile

Unique client record number: __ __ __ __ __ / __ __ __ __/ __ __ __ __ __ Initial visit date: dd / mm / yyyy

Name: First ________ Middle ________ Last ________ Telephone no: ________

Alien/National ID/passport/Birth Cert No: ________ NHIF No: ________ County of Birth ________

Sex: ☐ Male ☐ Female Date of birth: dd / mm / yyyy Age (years): __ __ If age <19, attends school: ☐ Yes ☐ No

Marital status (*select one*): ☐ Never married ☐ Cohabiting ☐ Married monogamous ☐ Married polygamous ☐ Separated/divorced ☐ Widowed

Population Type: ☐ Gen Population ☐ Discordant couple ☐ Key Population (Specify) → ☐ MSM ☐ MSW ☐ FSW ☐ PWID

B. Entry Point & Transfer Status

Referred from (*select one*):
☐ HBTC ☐ VCT site ☐ OPD ☐ MCH ☐ TB clinic ☐ IPD ☐ CCC
☐ Peer ☐ Outreach ☐ Self-referral ☐ Community ☐ Other: ________

If transferred in:
PrEP start date: dd / mm / yyyy Regimen: ☐ TDF-FTC ☐ TDF ☐ TDF-3TC
Facility transferred from: ______ MFL code: __ __ __ __ __ County: ______

C. Baseline Assessment

Behaviour risk assessment

Mark all that apply:

☐ Sex partner(s) is HIV+ and (*mark all that apply*):
- ☐ Not on ART
- ☐ On ART <6 months
- ☐ Suspected poor adherence to ART
- ☐ Detectable HIV viral load
- ☐ Couple is trying to conceive

(If yes to any) →

☐ Sex partner(s) high risk & HIV status is unknown
☐ Has sex with >1 partner
☐ Ongoing IPV/GBV
☐ Transactional sex
☐ Recent STI (past 6 months)
☐ Recurrent use of post-exposure prophylaxis (PEP)
☐ Recurrent sex under influence of alcohol/recreational drugs
☐ Inconsistent or no condom use
☐ Injection drug use with shared needles and/or syringes

Complete section if sex partner is HIV+

HIV+ partner CCC number: __ __ __ __ ___/__ __ __ __ __
or ☐ NA (not enrolled at a CCC)
or ☐ CCC number/enrollment status unknown

HIV+ partner ART start date: dd / mm / yyyy
or ☐ not on ART at initial visit

Time known to be HIV-serodiscordant: __ __ years + __ __ months

Sex without a condom with HIV+ partner in past 30 days: ☐ Yes ☐ No

Number of living children with HIV+ partner: __ __

Medical assessment & fertility intentions

Blood pressure (mm Hg): __ __ __ / __ __ __ Temperature: __ __ °C
Weight (kg): __ __ __ Height (cm): __ __ __
Signs/symptoms of STI : ☐ Yes; Use codes provided: ________ ☐ No

Chronic illnesses & comorbidities	Treatment
Liver disease: ☐ Yes ☐ No	
Kidney disease: ☐ Yes ☐ No	
1. Other description	
2. Other description	

***Male only*:**
Circumcised: ☐ Yes ☐ No ☐ Unknown

***Female only*:**
LMP: dd / mm / yyyy
Pregnant: ☐ Yes ☐ No
If pregnant: ☐ Planned ☐ Unplanned
Breastfeeding: ☐ Yes ☐ No
On family planning: ☐ Yes ☐ No FP methods: ______
Plan to have children (*select one*):
☐ Trying to conceive ☐ Future ☐ No ☐ Don't know

Clinical notes:

D. PrEP initiation

Lab results (*Investigations should not delay PrEP initiation. To be recorded when available.)*

Test	Result	Additional steps
Hepatitis B (HBsAg)	☐ Positive ☐ Negative ☐ Not done	If negative, vaccine series initiated: ☐ Yes ☐ No Date sample collected: dd/ mm / yyyy
Hepatitis C	☐ Positive ☐ Negative ☐ Not done	Date sample collected: dd/ mm / yyyy
Serum creatinine	__ __ __ (μmol/L) or ☐ Not done	If done, CrCl (mL/min): __ __ __ *If creatinine is out of range, or CrCl<50 mL/min, refer for further assessment.*

Previous PrEP use: ☐ Yes ☐ No Condom Issued: ☐ Yes ☐ No
Willing to start PrEP: ☐ Yes ☐ No Adherence Counseling Done: ☐ Yes ☐ No
If not willing, reason (*mark all that apply*): ☐ None ☐ Side effects (ADR) ☐ Stigma ☐ Pill burden ☐ Taking pills for a long time ☐ Too many HIV tests

Signs/symptoms of acute HIV: ☐ Yes ☐ **No**
Medically ineligible to start PrEP: ☐ Yes ☐ **No**
Contraindications for TDF-FTC /TDF-3TC/TDF: ☐ Yes ☐ **No**

Eligible for PrEP →
Prescribed PrEP at initial visit: ☐ **Yes** ☐ **No**
Regimen: ☐ TDF-FTC ☐ TDF ☐ TDF-3TC
# of months: __ __
Date of initiation: dd / mm / yyyy

Next appointment date: dd / mm / yyyy **Clinician initials:**

I. **Monthly refill form**

To be filled each month when the client is coming for a refill only in the months appearing under date of refill column else fill the follow up visit

Date of Refill	Behaviour risk assessment (Yes/No)	Adherence counselling (Yes/ No)	Continue /Discontinue PrEP (indicate appropriately)	Next appointment date	Remarks
Month 2				dd/mm/yyyy	
4				dd/mm/yyyy	
5				dd/mm/yyyy	
7				dd/mm/yyyy	
8				dd/mm/yyyy	
10				dd/mm/yyyy	
11				dd/mm/yyyy	
13				dd/mm/yyyy	
14				dd/mm/yyyy	
16				dd/mm/yyyy	
17				dd/mm/yyyy	
19				dd/mm/yyyy	
20				dd/mm/yyyy	
22				dd/mm/yyyy	
23				dd/mm/yyyy	
25				dd/mm/yyyy	
26				dd/mm/yyyy	
28				dd/mm/yyyy	
29				dd/mm/yyyy	
31				dd/mm/yyyy	
32				dd/mm/yyyy	
34				dd/mm/yyyy	
35				dd/mm/yyyy	
37				dd/mm/yyyy	
38				dd/mm/yyyy	
40				dd/mm/yyyy	
41				dd/mm/yyyy	
43				dd/mm/yyyy	
44				dd/mm/yyyy	
46				dd/mm/yyyy	
47				dd/mm/yyyy	
49				dd/mm/yyyy	
50				dd/mm/yyyy	
52				dd/mm/yyyy	
53				dd/mm/yyyy	
55				dd/mm/yyyy	
56				dd/mm/yyyy	

Follow Up Visit

Unique client record number: ___ ___ ___ ___ ___/ ___ ___ ___ ___/ ___ ___ ___ ___ ___ **Name of client**: ___________________________

Visit date: dd / mm / yyyy
Visit type: ☐ scheduled ☐ unscheduled

E. Medical assessment & fertility intentions

Clinical notes	Summary of findings	
	Blood pressure	___ ___ ___ / ___ ___ ___ mm Hg
	Weight	___ ___ ___ kg Temperature___ ___ºC
	Signs/symptoms of STI(s)	☐ yes ☐ no If yes Use codes provided________
	Signs/symptoms of acute HIV	☐ yes ☐ no
	If male, circumcised since last visit	☐ yes ☐ no ☐ na (already circumcised)

Possible adverse drug reaction ☐ none

1	*Description*
	☐ mild ☐ moderate ☐ severe ☐ life threatening ☐ not graded
	Action (*mark all that apply*) ☐ stop ☐ switched regimen ☐ Other ____________
2	*Description*
	☐ mild ☐ moderate ☐ severe ☐ life threatening ☐ not graded
	Action (*mark all that apply*) ☐ stop ☐ switched regimen ☐ Other ____________

Chronic illnesses & comorbidities		Treatment
	Liver disease ☐ Yes ☐ No	
	Kidney disease ☐ Yes ☐ No	
1	*Other description*	
2	*Other description*	

Plan to have children		☐ trying to conceive ☐ future ☐ no ☐ don't know ☐ client/partner is pregnant
If female	LMP: _______________ Pregnant	☐ yes ☐ no
	Breastfeeding	☐ yes ☐ no
	On family planning	☐ none or methods(Indicate the code):_________________
	If ended pregnancy since last visit	*Outcome date* dd / mm / yyyy
		Outcome ☐ term live ☐ preterm live ☐ induced abortion ☐ *loss*
		Birth defect(s) ☐ yes ☐ no ☐ don't know

F. Behaviour risk assessment

Mark all that apply

- ☐ Sex partner(s) is HIV+ and:
  - ☐ not on ART
  - ☐ <6 months ART use
  - ☐ poor adherence to ART
  - ☐ detectable HIV viral load
  - ☐ couple is trying to conceive
- ☐ Sex partner(s) at high risk for HIV & HIV status unknown
- ☐ Has sex with >1 partner
- ☐ Ongoing IPV/GBV
- ☐ Transactional sex
- ☐ Recent STI
- ☐ Recurrent use of PEP
- ☐ Recurrent sex under influence of alcohol/recreational drugs
- ☐ Inconsistent or no condom use
- ☐ IDU with shared needles/syringes

G. Follow up laboratory investigations

HIV test	☐ Positive ☐ negative ☐ not done	*If positive, collect sample for drug resistance. Client linked to care* ☐ Yes ☐ No
Serum creatinine (as per guidelines)	___ ___ ___ μmol/L or ☐ not done	*If creatinine is out of range, or CrCl <50 mL/min, refer for further assessment*
If creatinine done, CrCl ≥50 mL/min	☐ yes ☐ no	
Other (*write in test, results & units [if applicable]*)	1	
	2	

H. PrEP

Self-assessment of adherence since last visit	☐ Good ☐ Fair ☐ Bad ☐ n/a (did not pick up PrEP at last visit)
If Fair/ bad, reason(s) (*mark all that apply*)	☐ forgot ☐ lost/out of pills ☐ separated from HIV+ partner ☐ no perceived risk ☐ side effects ☐ sick
	☐ stigma ☐ pill burden ☐ shared with others ☐ none ☐ other ______________
Adherence Counseling done	☐ yes ☐ no **Condoms issued**: ☐ yes ☐ no
PrEP status	☐ continue ☐ restart ☐ discontinue
Prescribed PrEP today	☐ yes ☐ no
If yes, regimen and duration	☐ TDF-FTC ☐ TDF ☐ TDF-3TC number of months ___ ___
If discontinued, reason(s) (*mark all that apply*)	☐ HIV test is positive ☐ low risk of HIV ☐ renal dysfunction ☐ client request ☐ not adherent to PrEP
	☐ viral suppression of HIV+ partner ☐ too many HIV tests ☐ other ______________________

N[illegible] / yyyy **Clinician initials:**

Adherence

Adherence
Good: missed 0-3 doses in past 1 month
Fair: missed 4-5 doses in past 1 month
Bad: missed 6-7 doses in past 1 month

Creatinine clearance

$$\text{GFR (adult males)} = \frac{(140 - Age) \times 1.23}{serum\ creatinine\ (in\ micromol/L)}$$

$$\text{GFR (adult females)} = \frac{(140 - Age) \times 1.23}{serum\ creatinine\ (in\ micromol/L)} \times 0.85$$

FP Methods:
C = Condoms
TL = Tubal ligation/female sterilization
FA = Fertility awareness method/periodic abstinence
D = Diaphragm/cervical cap
LAM = Lactational Amenorrhea Method
IUD = Intra uterine device
IMP = Implant
INJ = Injectable
OC = oral contraceptive pills
ECP = Emergency contraceptive pills dispensed
V = Vasectomy (partner's)

STI Diagnosis:
Genital Ulcer Disease (GUD),
Vaginitis and/or Vaginal Discharge (VG),
Cervicitis and/or Cervical Discharge (CD),
Pelvic Inflammatory Disease (PID),
Urethral Discharge (UD),
Anal Discharge (AD),
Others (O)

If yes, regimen and duration	
If discontinued, reason(s) *(mark all that apply)*	

Next appointment date: dd / mm / yyyy   Clinician initials:

<u>Adherence</u>
Good: missed 0-3 doses in past 1 month
Fair: missed 4-5 doses in past 1 month
Bad: missed 6-7 doses in past 1 month

<u>Creatinine clearance</u>

GFR (adult males) = $\frac{(140-\text{[illegible]}) \text{ [illegible] } 1.23}{\text{[illegible]} \ (\text{[illegible]}/\text{[illegible]})}$

GFR (adult females) = $\frac{(140-\text{[illegible]}) \text{ [illegible] } 1.23}{\text{[illegible]} \ (\text{[illegible]}/\text{[illegible]})} \text{ X } 0.85$

<u>FP Methods:</u>
C = Condoms
TL = Tubal ligation/female sterilization
FA = Fertility awareness method/periodic abstinence
D = Diaphragm/cervical cap
LAM = Lactational Amenorrhea Method
IUD = Intra uterine device
IMP = Implant
INJ = Injectable
OC = oral contraceptive pills
ECP = Emergency contraceptive pills dispensed
V = Vasectomy (partner's)

<u>STI Diagnosis:</u>
Genital Ulcer Disease (GUD),
Vaginitis and/or Vaginal Discharge (VG),
Cervicitis and/or Cervical Discharge (CD),
Pelvic Inflammatory Disease (PID),
Urethral Discharge (UD),
Anal Discharge (AD),
Others (O)
