# Supplementary material for: Using an on-site modular training approach to amplify prep service delivery in public health facilities in Kenya
Source: PLOS Glob Public Health. 2022 Mar 10;2(3):e0000092. doi: 10.1371/journal.pgph.0000092 (PMC10021257; doi:10.1371/journal.pgph.0000092)

MINISTRY OF HEALTH

Pre-exposure Prophylaxis for the Prevention of HIV Infection:

A Toolkit for Providers

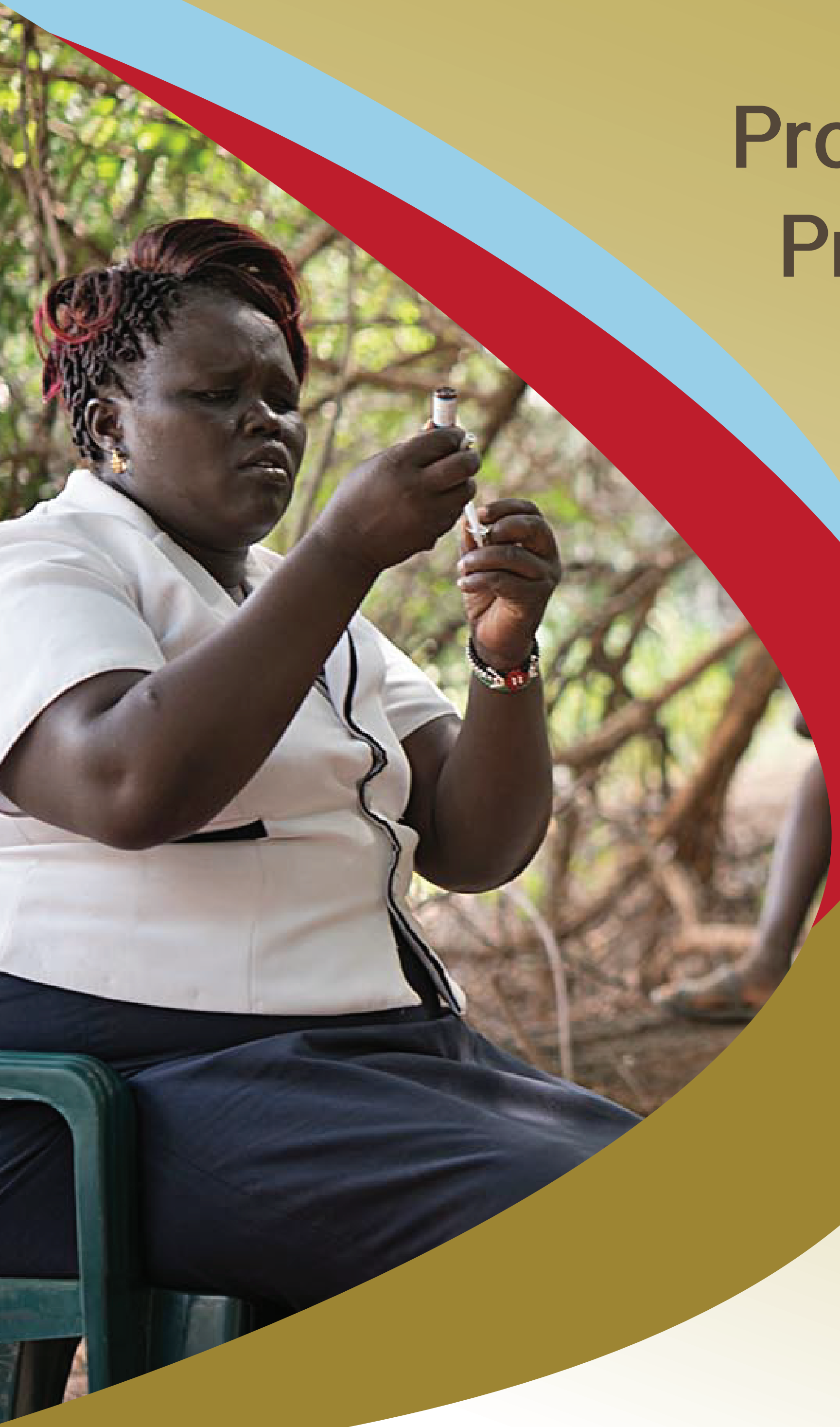

NASCOP

National AIDS and STI Control Program

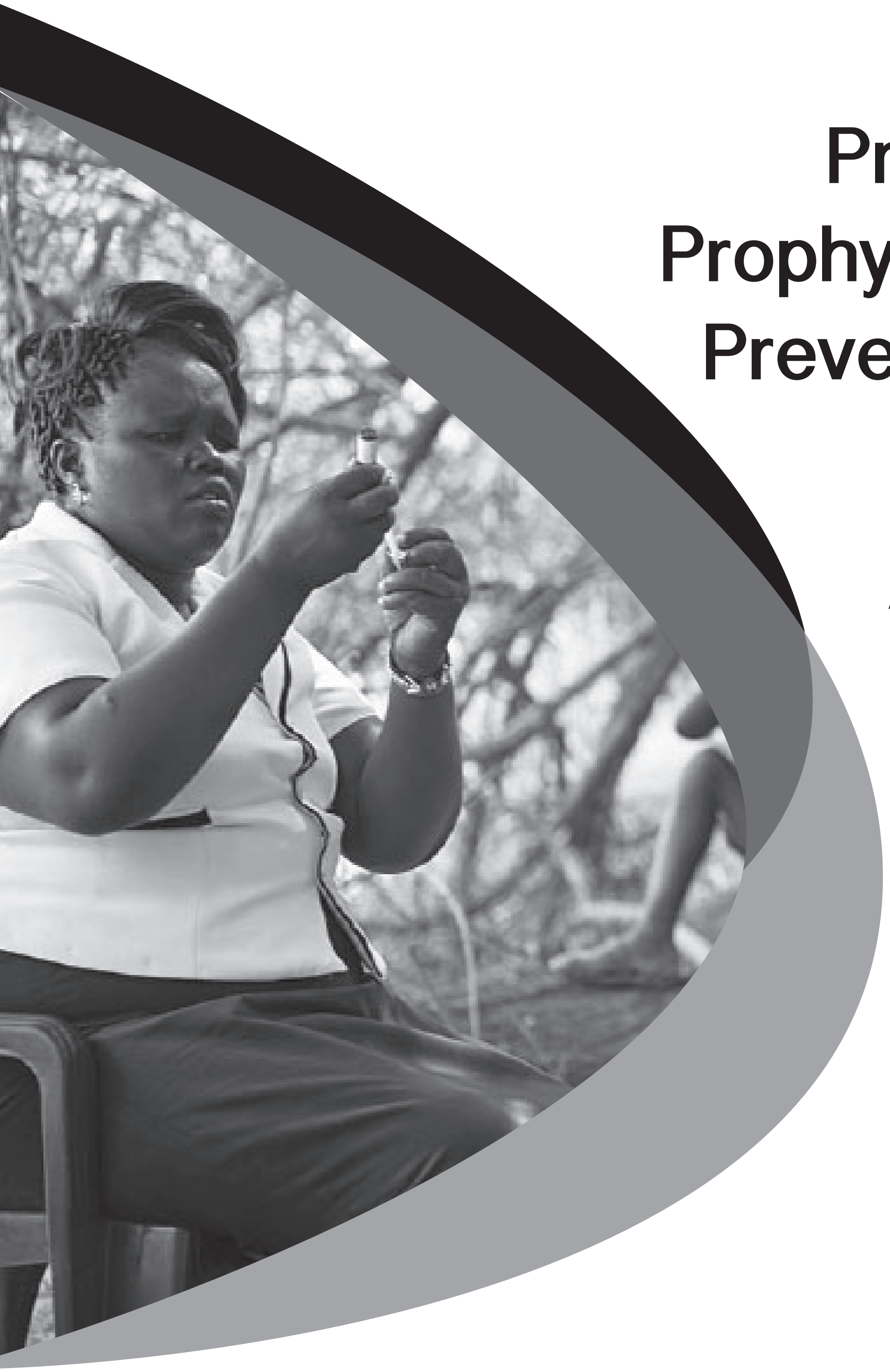

Pre-exposure Prophylaxis for the Prevention of HIV Infection:

A Toolkit for Providers

NASCOP

National AIDS and STI Control Program

© NASCOP

This toolkit is a publication of the National AIDS and STI Control Program, Ministry of Health, Kenya. You may print one copy for personal use. Bulk printing or any other of this toolkit requires written permission of NASCOP.

The purpose of this toolkit is to provide additional detailed information for healthcare workers to safely and effectively use PrEP as part of combination prevention of HIV infection. All reasonable precautions have been taken to verify the information contained in this toolkit. However, it is the responsibility of healthcare providers to cross-check and confirm the accuracy of any recommendations herein.

For clarifications contact National AIDS and STI Control Program (NASCOP) on P.O. Box 19361 00202, Nairobi Kenya, Tel: 254 775597297, Email: info@nascop.or.ke, Website: www.nascop.or.ke

Recommended citation for this toolkit is:
Ministry of Health, National AIDS & STI Control Programme (2017). Pre-exposure Prophylaxis for the Prevention of HIV Infection - A Toolkit for Health Service Providers, Nairobi, Kenya: NASCOP.

ISBN:

Foreword

Kenya has made tremendous progress in containing the HIV epidemic. For instance, the HIV prevalence in the country has dropped by nearly 50% from a peak of 10.6% in 1995-96 to approximately 5.9% in 2015. This has been made possible through the aggressive implementation of combination of evidence-informed interventions including scale-up of antiretroviral therapy. However, the decline in new infections (incidence) has remained modest with nearly 71,000 new infections occurring every year. Now, healthcare providers have expanding behavioural and biomedical interventions for HIV prevention, which if applied effectively will further reduce the number of new infections. Recent evidence has shown that daily oral antiretroviral agents, taken by HIV uninfected individuals at substantial ongoing risk of HIV infection, can significantly reduce the risk of HIV infection. On the basis of this evidence, the Ministry of Health reviewed the HIV treatment guidelines to incorporate guidance on pre-exposure prophylaxis for the prevention of HIV infection in Kenya.

'Pre-exposure Prophylaxis for the Prevention of HIV Infection - A Toolkit for Health Service Providers' was develop by NASCOP to support the implementation and scale-up of pre-exposure prophylaxis (PrEP). Good quality evidence from clinical trials and demonstration (pilot) projects has shown that PrEP, when used appropriately, is a safe and highly effective means of reducing the risk of HIV infection in HIV uninfected individuals at substantial ongoing risk of HIV infection.

To obtain the full benefits of its use, PrEP must be provided under the supervision of a trained healthcare provider, and as part of a combination of HIV prevention interventions tailored to each individual's vulnerability, risk profile and local HIV infection transmission determinants and burden. The provider will assess the client for suitability to use PrEP, exclude contra-indications to PrEP medications and offer ongoing monitoring, risk reduction and adherence support. PrEP is used only during periods increased ongoing risk of HIV infection. During follow-up, providers assess and determine whether PrEP is still necessary. The duration of PrEP use is determined by the level of risk by an individual PrEP user and the adoption and adherence to other HIV prevention interventions.

The purpose of this toolkit is to provide health service providers, agencies and institutions with succinct information and guidance to safely and effectively deliver PrEP. The toolkit contains information on indications for PrEP and guidance on assessment, starting, monitoring and discontinuing PrEP. Templates of data collection tools are provided as annexures.

It is my hope that all those concerned with health services delivery, will, with a sense of urgency, make PrEP available and accessible to all who may need it across the country. I am certain, this toolkit will contribute to increasing access to PrEP for HIV prevention in Kenya.

Dr Martin Sirengo
HEAD, NASCOP

Acknowledgements

This toolkit has been compiled through the collaborative effort of many individuals and institutions. The main source of information the toolkit was the 'Guidelines on the Use of Antiretroviral Drugs for Treating and Preventing HIV infection in Kenya, 2016 Edition'. The following institutions provided additional material and information for the toolkit: the DREAMSt, LVCT Health, Jhpiego and Partners Scale Up Project (University of Washington).

Financial and logistical support to develop and print the toolkit were provided by the United States Government through the Centers for Disease Control and Prevention-Kenya, the World Health Organization, the Clinton Health Access Initiative, LVCT Health and Jhpiego.

A comprehensive list of contributing organizations, individuals and reviewers is provided in Appendix 5.

Table of Contents

Foreword i

Acknowledgements ii

Table of Contents iii

List of Tables iv

List of Figures iv

Purpose 5

Section 1 Overview of Recommendations for Pre-Exposure Prophylaxis to Prevent HIV Infection 6

Combination HIV Prevention 8

Section 2 Risk Assessment and Indications for Pre-Exposure Prophylaxis 10

Indications for Pre-exposure Prophylaxis 10

Assessing for 'substantial ongoing' risk of HIV Infection 10

Excluding Acute HIV Infection 11

Managing high risk exposure within the last 72 hrs 11

Section 3 Initiating Pre-exposure Prophylaxis 13

Eligibility for PrEP 14

Prescribing Pre-exposure Prophylaxis 19

Section 4 Follow-up and Monitoring of Pre-Exposure Prophylaxis 20

Counselling Messages for PrEP 21

HIV Testing and Managing Suspected HIV Infection during PrEP 21

Improving adherence to PrEP 21

Assessing for medication side effects 22

Discontinuing PrEP 23

Restarting PrEP 23

PrEP in Special Circumstances 23

Section 5 Appendices 25

Appendix 1: Rapid Assement Screening Tool ..... **Error! Bookmark not defined.**

Appendix 2: Client Encounter record: Initaitaion and Follow Up 26

Appendix 3: PrEP Summary Reporting Tool 27

Appendix 4: Frequently Asked Questions about Pre-Exposure Prophylaxis 29

Appendix 5: List of Contributors and Reviewers 33

List of Tables

Table 1.1 Overview of Recommendations for Pre-exposure Prophylaxis 6
Table 2.1 Clinical Features and Symptoms of Acute HIV Infection 11
Table 3.1 Initial Assessment 14
Table 3.2 Managing Clinical and Laboratory Results on Initial and Follow-up Assessment 16
Table 3.3 Initial adherence preparation and counselling 17
Table 3.4 Pre-Initiation Education Check-list 17
Table 3.5 Pre-Initiation Assessment Check-list 19
Table 3.6 Recommended Regimen for Pre-Exposure Prophylaxis 19
Table 4.1 Adherence support during follow-up visits 22

List of Figures

Figure 1.1 Schema for Managing Pre-Exposure Prophylaxis for HIV Prevention 6
Figure 1.2 Combination Prevention of HIV Infection 8
Figure 2.1 Entry Points for PrEP and other HIV Prevention Services 12
Figure 3.1 Initiating Pre-Exposure Prophylaxis 13
Figure 4.1 Follow-up after initiating PrEP 20

Purpose

The purpose of this toolkit is to provide additional detailed information for healthcare workers to safely and effectively use PrEP as part of combination prevention of HIV infection.

Section 1:	Provides an overview of the recommendations for use of pre-exposure prophylaxis
Section 2:	Identifying clients at 'substantial ongoing risk of HIV infection' who could benefit from PrEP through behavioural risk assessment.
Section 3:	Contains information on initiating PrEP including client preparation through health education and adherence counselling; initial clinical and laboratory assessment and prescribing PrEP.
Section 4:	Contains guidance on monitoring clients on ART including clinical and laboratory monitoring, risk-reduction counselling, managing adverse events during PrEP, discontinuing and restarting PrEP, PrEP in special circumstances and combination prevention interventions
Section 5:	Contains appendices with examples of initial and follow-up clinical forms, registers and frequently asked questions

Section 1 Overview of Recommendations for Pre-Exposure Prophylaxis to Prevent HIV Infection

Table 1.1 Overview of Recommendations for Pre-exposure Prophylaxis

What is PrEP?	PrEP is a form of HIV prevention in which a HIV negative person at high risk of HIV infection takes daily oral antiretroviral agents to prevent HIV infection.
Who can take PrEP? (indications for PrEP)	PrEP is recommended for HIV negative persons at substantial ongoing risk of HIV infection such as<br>• In a discordant relationship, the sexual partner with HIV has not been on effective therapy for the preceding 6 months,<br>• Sexual partner/s of unknown HIV status and is/are at high-risk for HIV infection (has multiple sexual partners, has had STIs, engages in transactional sex, injects drugs, from high HIV burden settings)<br>• Engaging in transactional sex<br>• Recent sexually transmitted infection<br>• Recurrent use of post-exposure prophylaxis<br>• History of sex whilst under the influence of alcohol or recreational drugs as a habit<br>• Inconsistent or no condom use or unable to negotiate condom use during intercourse with persons of unknown HIV status<br>• Injection drug use where injection equipment is shared<br>• Sero-discordant couples trying to conceive<br>• Requests for PrEP and meets any of the above eligibility criteria
Contraindications to PrEP	• HIV infection (confirmed HIV positive)<br>• Renal impairment - as shown by creatinine clearance < 50 ml/min<br>• Lack of willingness to adherence to daily PrEP and associated follow-up schedule<br>• Adolescents < 35kgs or age < 15 years
Initiating PrEP	PrEP is initiated only after thorough behavioural and risk assessment (to establish level of risk and willingness to use PrEP); and clinical and laboratory evaluation (to exclude HIV infection and establish safety to use PrEP). Clients should also receive adequate adherence and ongoing risk reduction counselling.
What are the recommended PrEP medications?	*Preferred*<br>• TDF/FTC (300 mg/200 mg) as FDC once daily<br>*Alternative*<br>• TDF 300 mg once daily<br>• TDF/3TC 300 mg/300 mg as FDC once daily<br>*At initiation, prescribe PrEP for only 30 days to allow for follow-up visits to assess adherence, tolerability and commitment to continue with PrEP. Subsequently, longer prescriptions may be given; however, the medicines should be issued monthly.*
What is effective PrEP use?	PrEP should be offered as part of a comprehensive, individualized prevention plan following behavioural risk assessment and adherence counselling. Combination prevention includes:<br>• Risk reduction counselling<br>• Safer sex practices<br>• Consistent & correct condom use<br>• VMMC (where indicated)<br>• Prevention and treatment of STIs<br>• Substance abuse treatment<br>• Prevention of gender-based violence (GBV)<br>• Adherence to PrEP - efficacy of PrEP is dependent on adherence.<br>• Effective cART for HIV+ persons (Treatment as Prevention)<br>• After initiation PrEP will be effective after a minimum of 7 days of consistent use.
Follow-up	After starting PrEP, clients require regular follow-up (initially at 1 month) then every 3 months thereafter (i.e months 1, 3, 6, 9, 12, 15, 18 etc) to monitor HIV status (every 3 months), for risk reduction counselling, adherence assessment and support, side effects. Obtain creatinine annually; or earlier/more frequently if clinically indicated.
Duration	PrEP is not meant to be a lifelong intervention. It is a method of HIV prevention during periods when a person is at greatest risk of acquiring HIV.
Discontinuation of PrEP	PrEP should be discontinued in any of the following circumstances<br>● HIV positive ● change in risk (to low risk) ● renal adverse effect (CrCl < 50 ml/min) ● sustained non-adherence ● sustained viral suppression in the HIV partner of a discordant couple ● client request to discontinue.

Figure 1.1 Schema for Managing Pre-Exposure Prophylaxis for HIV Prevention

Initial Visit/First Contact
Perform a behavioural risk assessment - to determine if PrEP is indicated. If PrEP is indicated:
- Discuss effective PrEP use, clarify misinformation
- Perform initial clinical and laboratory assessment
  - Baseline HIV test, and rule out acute HIV infection
  - Screen for STIs and risk of renal disease/hepatitis B&C infection

→

If HIV negative and meets eligibility criteria for PrEP
- Offer adherence counselling
- Prescribe 30 day supply of PrEP (TDF 300 mg/FTC 200 mg OD)
- Discuss combination prevention and risk reduction

↓

30 day review
- Assess for adherence and offer adherence counselling and support
- Ask about any side effects. Find out how the client is coping with the side effects. Reassure if minor
- Assess for STIs,(and pregnancy in women)) and risk of acute HIV infection
- Perform a HIV test
- Assess for risk of kidney disease, if available, obtain serum creatinine and calculate creatinine clearance
- Discuss risk reduction and provide condoms

Prescribe 60 days of PrEP, schedule review after 60 days for monthly drug refills.

3-month review
- Assess for adherence and offer adherence counselling and support
- Ask about any side effects. Find out how the client is coping with the side effects. Reassure if minor
- Assess for STIs, (and pregnancy in women) and acute HIV infection
- Perform HIV test
- Discuss risk reduction and provide condoms

Give a 90-day PrEP prescription for monthly drug refill of PrEP. Schedule the 6-month return visit.

6-month review
- Assess for adherence and offer adherence counselling and support
- Ask about any side effects
- Assess for STIs, (and pregnancy in women) and acute HIV infection
- Perform HIV test
- Discuss risk reduction and provide condoms

Give a 90-day PrEP prescription for monthly drug refill of PrEP. Schedule the 9-month return visit.

9-month review
- Assess for adherence and offer adherence counselling and support
- Ask about any side effects
- Assess for STIs, (and pregnancy in women) and acute HIV infection
- Perform HIV test
- Discuss risk reduction and provide condoms. Discuss need for continued PrEP

Give a 90-day PrEP prescription for monthly drug refill of PrEP. Schedule the 12-month return visit.

12-month review
- Assess for adherence and offer adherence counselling and support
- Ask about any side effects
- Assess for STIs, (and pregnancy in women) and acute HIV infection
- Perform HIV test
- Discuss risk reduction and provide condoms. Discuss need for continued PrEP
- Do serum creatinine test and calculate creatinine clearance

Note: Risk and adherence assessment and support should be offered during each visit including at dispensing refill visits.

Combination HIV Prevention

PrEP should not be provided in isolation, but as part of a package of combination prevention individualized to a client's preference, characteristics, risk profile and local HIV disease burden. Figure 1.2 summarizes steps for combination prevention for clients accessing PrEP services.

Figure 1.2 Combination Prevention of HIV Infection

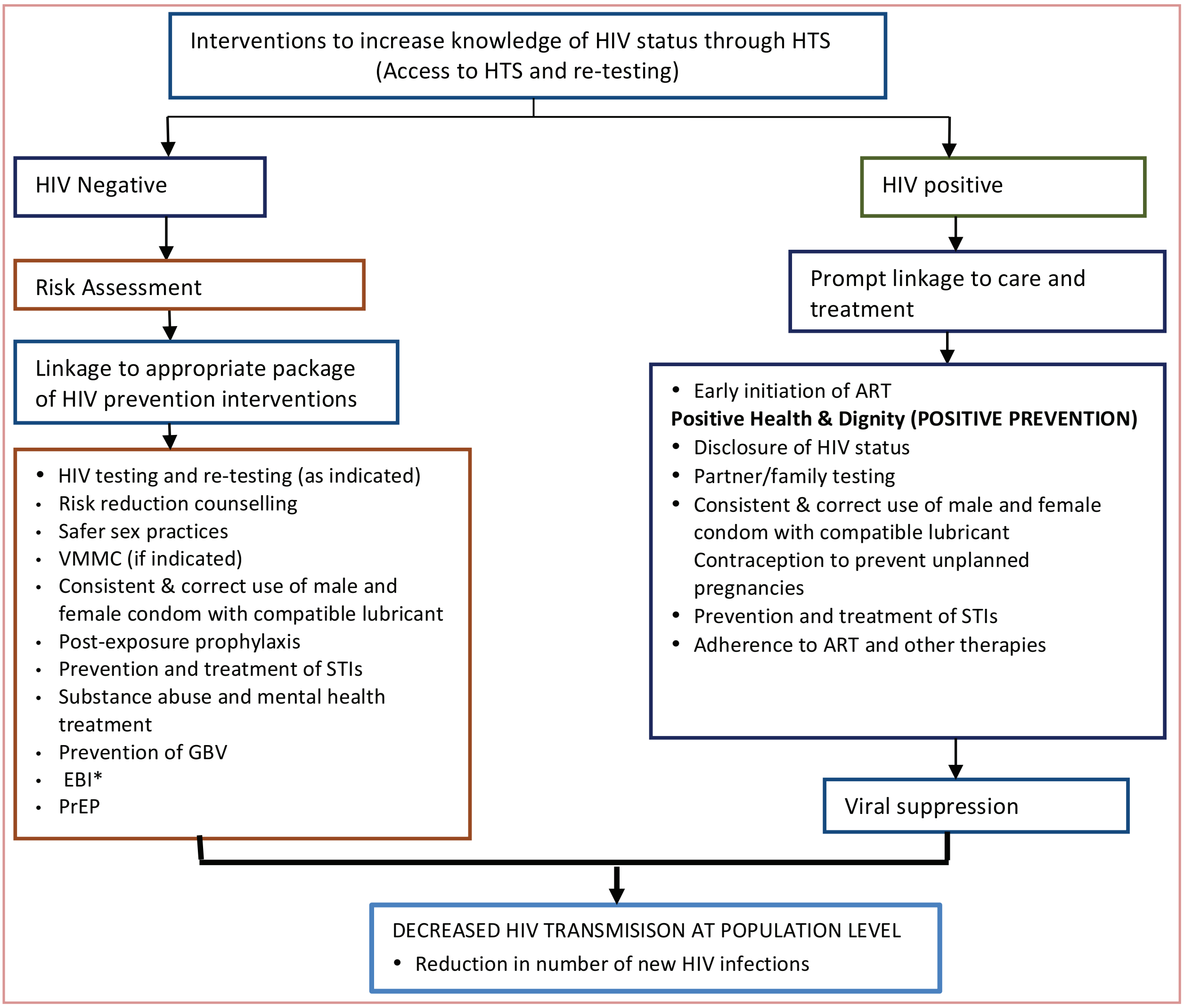

***Evidence Based Behavioural Interventions**

a. *Health Choices I & II*: This intervention targets sexually and non-sexually active youth and youth living with HIV (10 – 13 years and 14 to 17 years). The interventions involves sessions on decision making, sex communication, negotiation and refusal skills with the aim of delaying sexual debut, promoting safer sex practice, HIV and STI risk reduction and condom use.
b. *EBAN*: This intervention is for discordant couples and entails training the couples in assertive communication skills, overcoming barriers to negotiating for safer sex and emphasis on partner involvement in safer sex.
c. *Sister to sister*: is delivered by female healthcare workers and peer educators targeting women 10 to 45 years of age aimed at reducing or eliminating risk behaviour and preventing HIV and STIs through self-efficacy and condom negotiation.
d. *Shuga* : This is a multimedia intervention targeting youth (15-24 years) out of school focusing on reducing sexual concurrence, correct and consistent condom use, personal risk perception, stigma and discrimination, transactional sex, gender based violence and parent-child communication..
e. *RESPECT*: This is a 2-session intervention for key populations and individuals at high risk focusing on risk reduction counselling.
f. *START*: Targets released prisoners (males 18 to 29 years) returning to the community. Focuses on increasing awareness of risk of HIV and STIs and offers tools to reduce risk.
g. *IMAGE*: This is an intervention for young girls and women of reproductive age. This intervention rides on economic empowerment initiatives to provide HIV and GBV training and risk reduction.

Section 2 Risk Assessment and Indications for Pre-Exposure Prophylaxis

PrEP for prevention of HIV infection is **only** indicated for HIV negative individuals at **substantial ongoing** risk of HIV infection.

Indications for Pre-exposure Prophylaxis

- An individual whose sexual partner is known to be HIV positive and: not on ART, or on ART but has not achieved viral suppression (often ART for less than 6 months), or on ART but with suspected poor adherence.
- A person whose sexual partner/s are of unknown HIV status and are at high-risk for HIV infection (multiple sexual partners, history of STIs, transactional sex, injection drug use or from high HIV burden settings
- Engaging in transactional sex
- History of recent or current sexually transmitted infection
- Recurrent use of post-exposure prophylaxis
- History of sex whilst under the influence of alcohol or recreational drugs as a habit
- Inconsistent or no condom use or unable to negotiate condom use during intercourse with persons of unknown HIV status
- Injection drug use where needles and syringes are shared
- Sero-discordant couples trying to conceive

Assessing for 'substantial ongoing' risk of HIV Infection

Screening questions are used to identify (for further discussions and assessment) individuals may be offered PrEP based on personal circumstances, risk and desire for additional HIV prevention. The questions are framed to elicit people's behaviours and vulnerabilities as opposed to specific sexual practices.

Before starting the sexual behavioural assessment,

- Ensure adequate privacy
- Assure the patient of confidentiality and indicate that the issues to be discussed may be very personal and that he/she is free to answer or decline
- Explain that this is routine practice to help provide appropriate sexual and reproductive healthcare
- Stress that that findings from the conversation will be kept confidential and only used for purposes of providing better care
- Make the patient comfortable

General Screening Questions

Preamble statement: I wish to know more about your sexual life. Some of these questions may not be comfortable but are important in helping to explore your risk of HIV infection. I would request that you answer honestly and openly. All the information you provide will be kept confidential and will only be used to better meet your health needs.

In the past 6 months,

- "Have you had sex with more than one person?"
- "Have you had sex without a condom?"
- "Have you had sex with anyone whose HIV status you do not know?"
- "Are any of your partners at risk of HIV?"
- "Do you have sex with a person who has HIV?"
- "Have you received a new diagnosis of a sexually transmitted infection?"
- "Do you desire pregnancy?"
- "Have you used or wanted to use PEP or PrEP for sexual exposure to HIV?"
- Have you injected drugs that were not prescribed by healthcare provider? If yes, did you use syringes, needles or other drug preparation equipment that had already been used by another person?
- "Received money, housing, food or gifts in exchange for sex?"
- "Been forced to have sex against your will?"
- "Been physically assaulted, including assault by a sexual partner?"

Screening Questions for People in Discordant Relationships
For the HIV negative individual in a discordant relationship, the following screening questions help to establish the need for PrEP

- "Is your partner taking ART for HIV?"
- "Has your partner been on ART for more than 6 months?"
- "At least once a month, do you discuss whether your partner is taking therapy daily?"
- "If you know, when was your partner's last HIV viral load test? What was the result?"
- "Do you desire pregnancy with your partner?"
- "Do you use condoms every time you have sex?"

(Refer to the Appendix 1: Rapid Assessment Screening Tool)

Excluding Acute HIV Infection
Inquire about the presence of fever, fatigue, myalgia, rash, headache, sore throat, cervical adenopathy, arthralgia, night sweats, or diarrhoea; with high risk exposure to HIV infection within the past month.

Table 2.1 Clinical Features and Symptoms of Acute HIV Infection

**Feature**	**Frequency (%)**
Fever	75
Fatigue	68
Muscle pain	49
Skin rash	48
Headache	45
Sore throat	40
Cervical adenopathy	39
Arthralgia	30
Night sweats	28
Diarrhoea	27

Managing high risk exposure within the last 72 hrs
In HIV seronegative clients who have had a high risk exposure to HIV within the last 72 hours, provide PEP for 28 days. Obtain a rapid HIV test at 28 days, if the test result is negative, transition to PrEP immediately (if the client is eligible for PrEP)

Figure 2.1 Entry Points for PrEP and other HIV Prevention Services

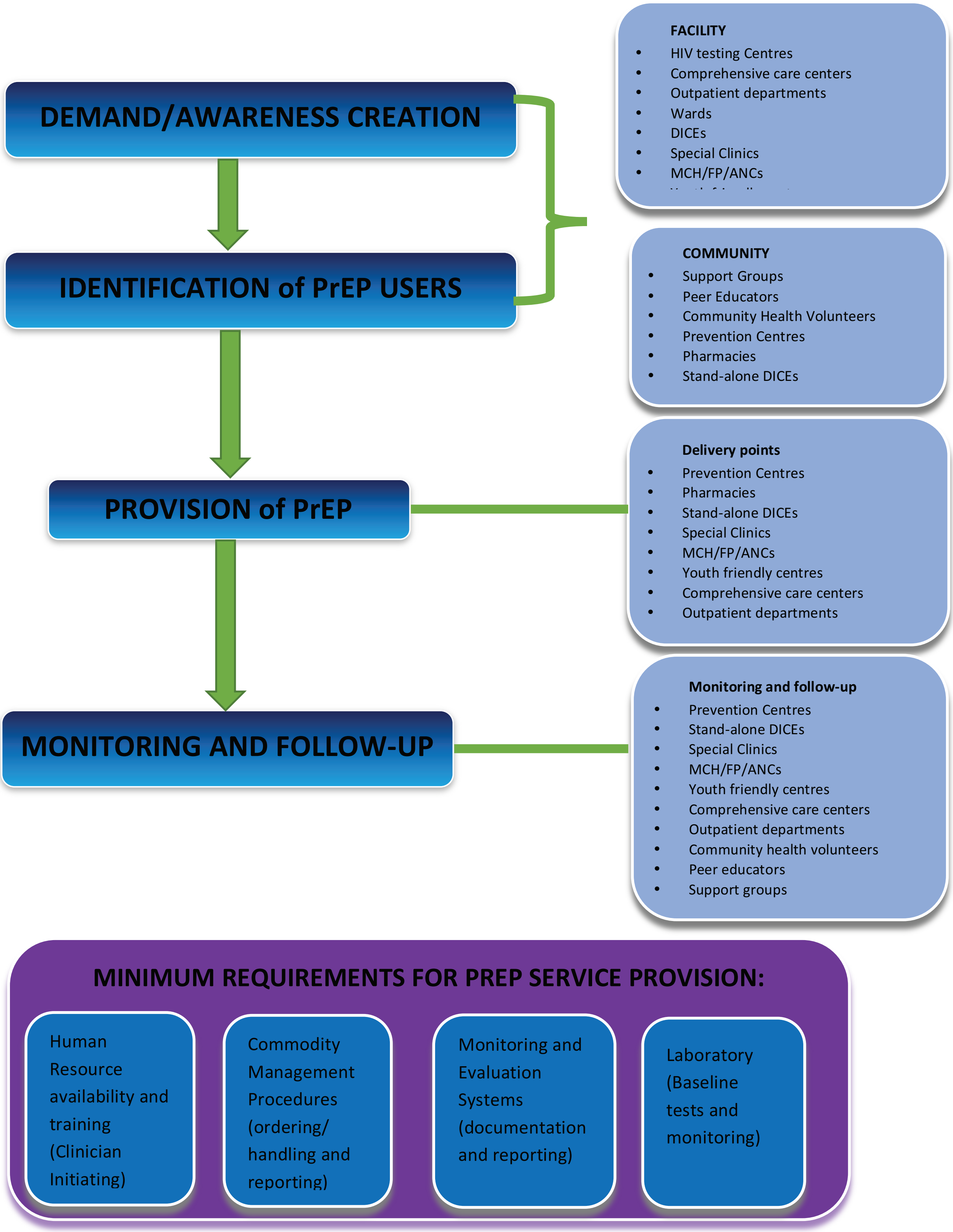

Section 3 Initiating Pre-exposure Prophylaxis

PrEP should only be initiated after a clinical and laboratory assessment and adequate preparation through health education and adherence counselling and support. Figure 3.1 provides the overview of the requisite steps before a client is started on PrEP.

Figure 3.1 Initiating Pre-Exposure Prophylaxis

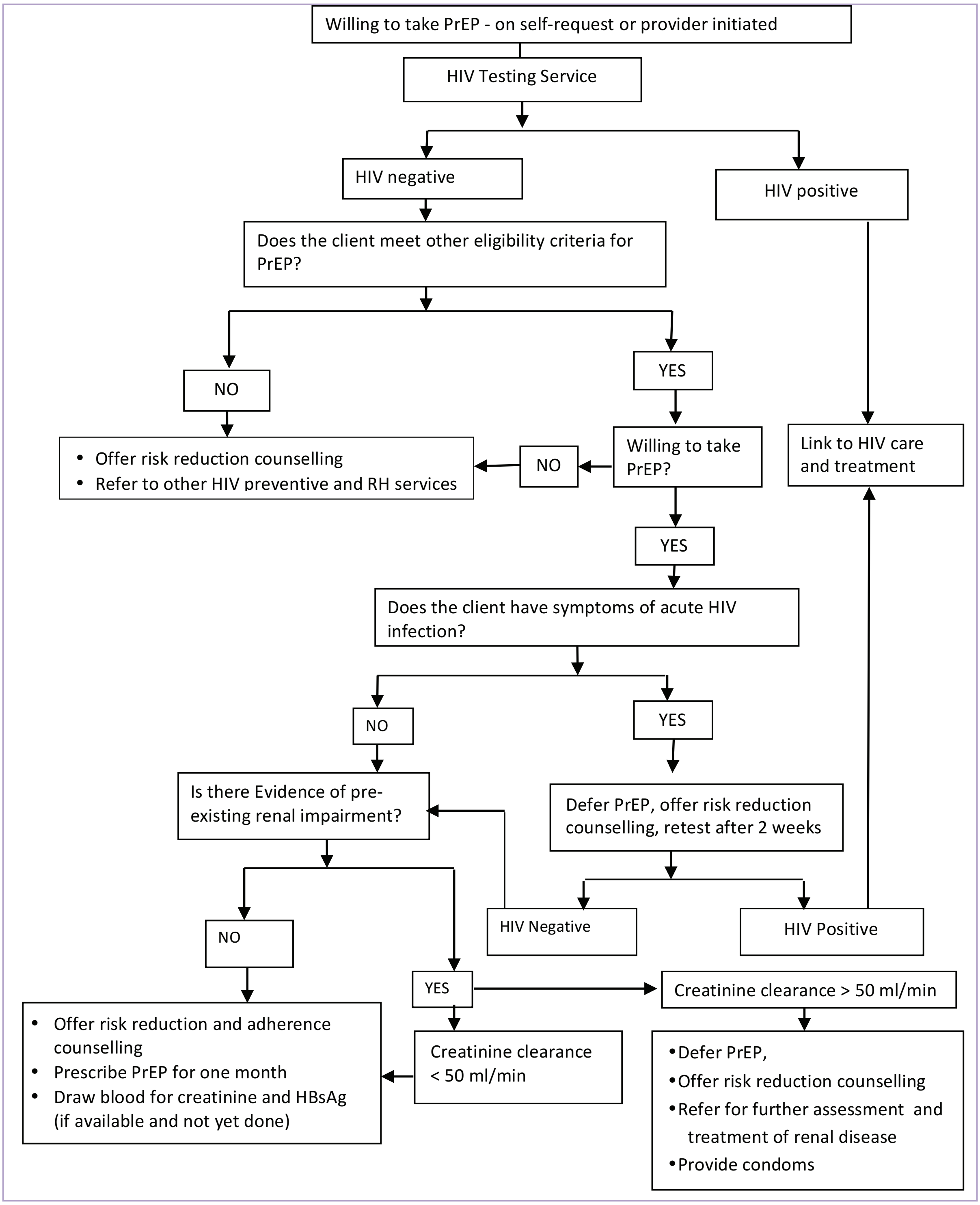

Eligibility for PrEP

Potential PrEP users must meet all of the following eligibility criteria prior to initiating PrEP

- substantial on going risk of HIV infection
- no suspicion of acute HIV infection
- documented HIV negative test
- no contraindications to PrEP medications (TDF/FTC or TDF/3TC or TDF)
- willingness to use PrEP as prescribed, including regular visits to monitor HIV status, adherence and side effects

Once a decision is made that a client requires PrEP, further assessment (listed in Table 3.1 below) should be carried out to establish safety and suitability of PrEP for the individual client.

This will be documented in the Client Encounter record (Appendix 2)

Table 3.1 Initial Assessment

Assessment/Service	Rationale
Complete medical history and examination	• • Identify medical conditions that could affect the management of PrEP<br>○ Past or current kidney disease<br>○ Risk of kidney disease (diabetes mellitus, uncontrolled hypertension, chronic NSAID use<br>○ Use of other nephrotoxic agents<br>○ Past of current liver disease<br>○ Current or past chronic hepatitis (B or C)<br>○ Acute HIV infection. If acute HIV infection is suspected, defer PrEP until HIV infection is excluded.
Establish eligibility to use PrEP	• Establish willingness to adhere to PrEP and medical follow-up including HIV retesting<br>• Screen for substantial risk of HIV infection<br>• Document HIV status - HIV testing using the national algorithm for HTS<br>• To complete a symptom checklist to exclude acute HIV infection
Baseline laboratory investigations*	*Urinalysis*<br>• Proteinuria is an early indicator of TDF toxicity. An initial urinalysis helps identify pre-existing proteinuria and risk of renal disease and therefore additional testing (creatinine) and closer monitoring after initiation of PrEP
	Serum creatinine and creatinine clearance<br>• To identify pre-existing renal dysfunction. PrEP is contraindicated if the baseline CrCl < 50 ml/min
	Hepatitis B surface antigen<br>• To identify undiagnosed current hepatitis B infection. If negative, consider vaccination against hepatitis B. [Refer to the national guidelines on hepatitis prevention and treatment]
	Hepatitis C antibody (especially in people who inject drugs, PWID).<br>• If positive, consider treatment for hepatitis C infection.
	Rapid Plasma Reagin<br>• To diagnose and treat syphilis infection.
	Pregnancy testing<br>• To guide antenatal care, contraceptive and safer conception counselling, and to assess risk of mother to child transmission. Pregnancy is not a contraindication to PrEP use.
Screening for other STIs	Assess for presence of dysuria, discharge, anorectal itching or pain, rash, or ulcers. To diagnose and treat STI (syndromic or diagnostic STI testing, depending on local guidelines).

Review vaccination history	Consider vaccination for hepatitis A, human papilloma virus, tetanus and meningitis.
Brief counselling	• Assess whether the client is at substantial risk of HIV.<br>• Discuss prevention needs and provide condoms and lubricants.<br>• Discuss desire for PrEP and willingness to take PrEP.<br>• Develop a plan for effective PrEP use, sexual and reproductive health.<br>• Assess fertility intentions and offer contraception or safer conception counselling.<br>• Assess intimate partner violence and gender-based violence.<br>• Assess substance use and mental health issues.<br>• If proceeding to offer PrEP, offer detailed initial adherence counselling (Table 3.3)

Table 3.2 Managing Clinical and Laboratory Results on Initial and Follow-up Assessment

Screening	Action
HIV-positive at initial evaluation	Do not start PrEP, counsel and link to care and treatment
HIV-positive after initiation of PrEP	Discontinue PrEP, counsel and link to care and treatment
Positive STI screen	Thorough genitourinary and anorectal examination, urine dipstix for urethritis, serological testing for syphilis, full STI evaluation if resources available. Refer to guidelines on syndromic management of STIs.
HBsAg-negative	Offer HBV vaccination
HBsAg-positive	This is not a contraindication to PrEP. However, will require monitoring of liver function and referral for management of liver disease.
Flu-like illness after initiating PrEP	Continue PrEP, test for HIV at first contact and after 28 days, and if negative, continue with usual follow-up.
Side effects of PrEP	**GIT** - nausea, vomiting, weight loss: these are often mild, self-limiting and occur during the first 1-2 months. Provide supportive counselling, offer symptomatic treatment e.g. anti-emetics like metoclopramide 10 mg 8 hourly for 3 to 5 days.
	**Renal** - transient increase in creatinine, and rarely proteinuria and Fanconi's syndrome (presenting as polyuria, bone pain and weakness). Where available, measure creatinine (and calculate estimated creatinine clearance) at initiation of PrEP, and annually thereafter or whenever indicated (symptom directed); or earlier/more frequently if at risk of renal disease. If creatinine clearance (eGFR) < 50 mL/min; do not start PrEP, refer for evaluation of underlying renal disease. If the renal function returns to normal, reassess for PrEP and initiate/continue PrEP (if still indicated) Monitor closely for recurrence of renal impairment. PrEP should not be prescribed for individuals using nephrotoxic drugs like acyclovir, aminoglycosides, retinoids, instead, discuss and provide alternative HIV prevention options.
Pregnancy or breastfeeding	Pregnancy and breastfeeding are not contraindications to use of PrEP. Pregnant or breastfeeding women whose sex partners are HIV positive or are at high risk of HIV infection may benefit from PrEP as part of combination prevention of HIV infection. PrEP is also indicated for HIV-negative in discordant partnerships who wish to conceive. PrEP in these situations can be prescribed during the pre-conception period and throughout pregnancy to reduce risk of sexual HIV infection.

Table 3.3 Initial adherence preparation and counselling

Theme	Adherence message/action
Climate Setting	Introduce yourself to the client, giving your name and role; ensure adequate privacy and reassure about confidentiality
What is PrEP?	PrEP involves HIV-negative people taking daily ARV medications to prevent themselves from becoming infected with HIV. PrEP is provided as part of combination prevention, including efforts at ongoing risk reduction
Does PrEP work?	Evidence from scientific studies involving HIV negative people at risk of HIV infection has shown that PrEP is highly effective if you take it as prescribed and in combination with other HIV prevention interventions.
How is PrEP used?	• PrEP is provided as tablets. You should take one tablet daily at the same most convenient time of day. To ensure you do not forget take PrEP each day:<br>o Make it a habit linked to an activity you do daily such as brushing teeth, taking a meal etc<br>o Disclose PrEP use to a partner or trusted person<br>o Use reminder devices like a cell phone alarm<br>o If available enrol into an SMS reminder system<br>• If you forget to take a tablet, take it as soon as you remember; however, do not exceed 2 tablets in a day. PrEP tablets can be taken any time of day, with or without food<br>• PrEP use is a personal, responsible choice to protect yourself and your sexual partners from HIV. Discussing PrEP use with trusted friends or other PrEP users may be helpful<br>• PrEP can be used safely with family planning pills or injections
Starting PrEP	• You will need a HIV test before starting or re-starting (if you had stopped) PrEP. This is to ensure that you do not already have HIV infection before starting PrEP because PrEP is not effective in treating existing HIV infection.<br>• It takes up to 7 days of daily used of PrEP tablets to achieve maximum protection. During this period, and as much as possible, you are encouraged to practice safer sex practices especially consistent, correct used of male or female condoms.
Stopping PrEP	Discuss stopping PrEP with your provider. You can stop using PrEP 28 days after your last possible HIV exposure. People can stop PrEP if they are no longer at substantial risk of acquiring HIV infection. Ways to lower risk include:<br>• Adopting safer sexual practices, such as abstinence, or using condoms during all sexual contacts;<br>• Following viral suppression in a sero-discordant couple;<br>• Leaving sex work;<br>• Ceasing injection drug use or the sharing injection drug use equipment
Protection from other STIs	PrEP does not offer protection from other STIs such as gonorrhoea, syphilis, herpes etc. Discuss with your provider if you suspect that you have an STI (genital sores or discharge). Using a condom each time you have sex will provide additional protection from HIV and other STIs
PrEP safety	● TDF-based PrEP is generally safe and well tolerated.<br>● Gastrointestinal symptoms are the most common. They include nausea, diarrhoea, vomiting decreased appetite, abdominal cramping or flatulence; dizziness or headaches. Typically, these symptoms start in the first few days or weeks of PrEP use and last a few days and almost always less than 1 month. Discuss with your provider if these side effects are severe or they persist for longer than one month.<br>• A few people may not be able to use PrEP due to kidney-related side effects
Prevention of pregnancy	PrEP does not prevent pregnancy. Use effective contraception unless you want pregnancy. If you want to become pregnant, discuss with your provider about safer ways to conceive.
PrEP during pregnancy and breastfeeding	PrEP can be used safely during pregnancy and breastfeeding. The risk of HIV infection is higher during pregnancy and breastfeeding. It is also easier to pass HIV to the unborn or breastfeeding baby if HIV infection occurs during pregnancy or breastfeeding. PrEP does not interfere with male or female fertility.
Client concerns	Clarify misconceptions, address any client concerns

Table 3.4 Pre-Initiation Education Check-list

<table>
<tr><th colspan="2">Ensure that at least the following aspects during client counselling and education</th></tr>
<tr><td>How PrEP works as part of combination prevention</td><td>Explain the need for baseline and follow-up tests including HIV testing.</td></tr>
<tr><td>Limitations of PrEP
• Link efficacy to adherence
• PrEP reduces but does not eliminate the risk of acquiring HIV
• PrEP does not prevent pregnancy or other STIs
• May not be suitable in clients with renal impairment or intolerance to the PrEP medicines</td><td>Discuss when and how PrEP may be discontinued.</td></tr>
<tr><td>PrEP use
• The medications used (show the client the pills)
• How the medications are used (daily)
• Number of daily doses required to achieve efficacy (7)
• What to do when doses are missed?
• Discontinuation of PrEP (need to continue for 28 days from last potential exposure to HIV)

Safety and side effects and what to do in case these are experienced.</td><td>What to do in case of client experiences symptoms of sero-conversion (acute HV infection)</td></tr>
<tr><td colspan="2">Risk reduction counselling and support
• Education (risk and safer sex practices)
• Managing mental health needs
• Couple counselling
• Access to, and consistent use of condoms and lubricants
• Access to and need for frequent HIV testing
• Early access to ART for those who test HIV positive
• VMMC (if indicated)
• STI screening and treatment
• Harm reduction for PWID</td></tr>
</table>

Table 3.5 Pre-Initiation Assessment Check-list

Confirm the following have been done prior to prescribing PrEP
HIV testing and counselling, HIV-negative
Symptoms of acute HIV infection
Behaviour risk assessment
Substance use and mental health screening
Partner information (where available/known)
Pre-initiation education and understanding of PrEP
Readiness and willingness to adhere to prescribed PrEP and follow-up schedule
STI screening and treatment
**For Women**<br>Pregnancy test<br>**Pregnancy and pregnancy intention**<br>Is the client currently using any contraception?<br>If not, is she interested in using long-term hormonal contraception in addition to condoms?<br>Is the client trying to conceive?<br>Is the client pregnant or breastfeeding?
Serum creatinine and creatinine clearance >50 mL/min
HBsAg
HCV serology (for PWID)
Medication history

Prescribing Pre-exposure Prophylaxis

Table 3.6 provides the recommended regimen for PrEP. The first prescription should be for 30 days to allow for scheduling for the first follow-up visit to assess adherence, tolerability and adverse effects. Subsequently, a 3-month prescription can be given. However, drug refills are done monthly.

Table 3.6 Recommended Regimen for Pre-Exposure Prophylaxis

**Preferred**	TDF 300 mg/FTC 200 mg once daily as FDC
**Alternative 1**	TDF 300 mg once daily
**Alternative 2**	TDF 300/FTC 300 mg once daily as FDC

This will be documented in the Client Encounter record (Appendix 2)

Section 4 Follow-up and Monitoring of Pre-Exposure Prophylaxis

PrEP should only be prescribed to clients who demonstrate good understanding of/and commitment to regular follow-up visits, initially after one month and at least every 3 months thereafter. The objectives of the follow-up visits are to:

- Assess adherence and provide ongoing adherence counselling and support
- Monitor for and manage side effects
- Exclude HIV infection
- Provide other prevention services including risk reduction counselling, condoms, STI screening and treatment, substance abuse treatment etc
- Review indications for PrEP

Figure 4.1 Follow-up after initiating PrEP

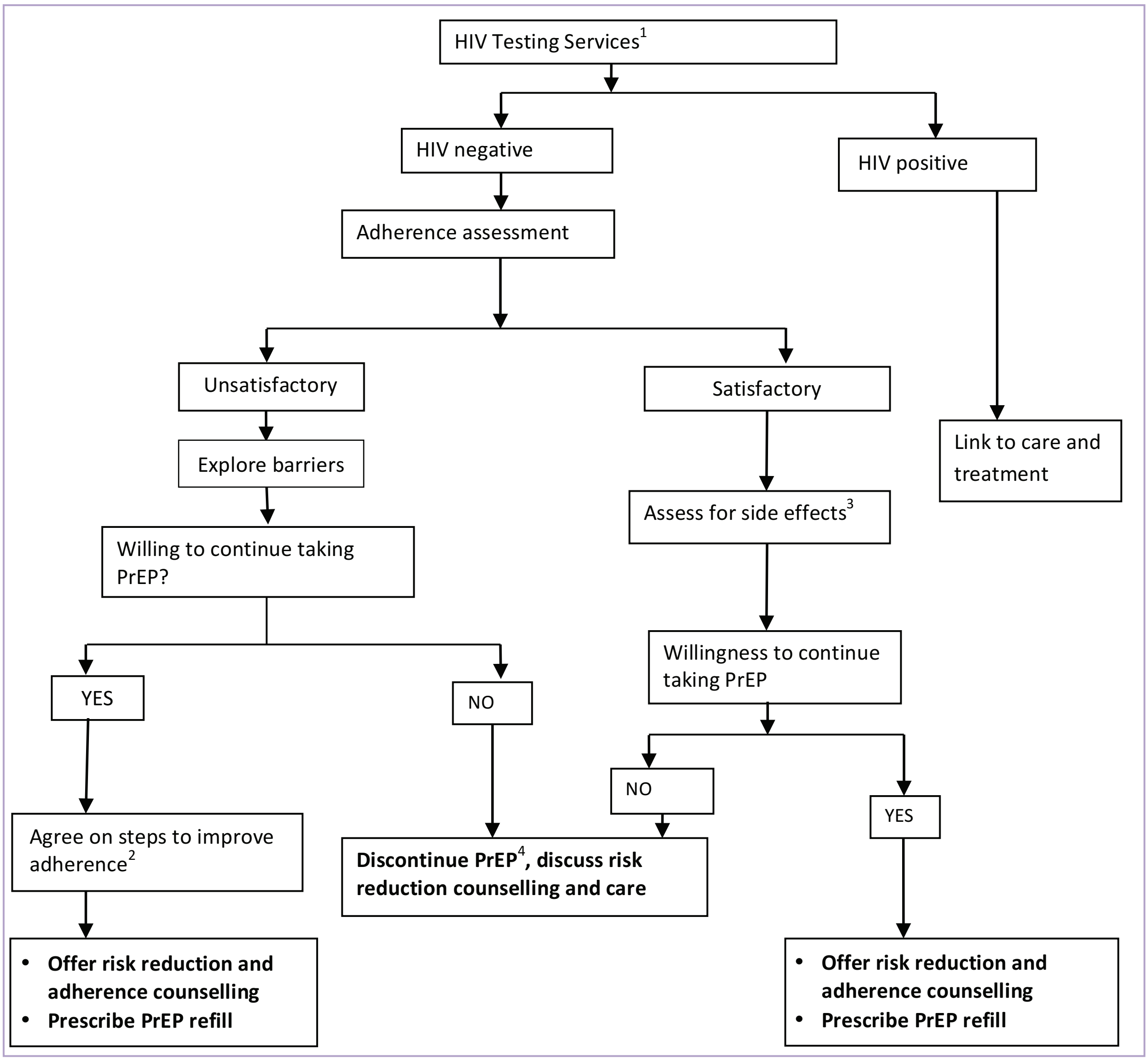

This will be documented in the Client Encounter record (Appendix 2) and summarised in the PrEP summary reporting tool (Appendix 3)

Counselling Messages for PrEP

- **PrEP** is not to be taken for life unless one has on-going risk
- PrEP works when taken correctly as prescribed by the health care providers
- For discordant couples, PrEP is a bridge before the positive partner achieves complete viral suppression
- PrEP is safe in pregnancy and during breastfeeding

HIV Testing and Managing Suspected HIV Infection during PrEP

a. **Routine HIV Testing during PrEP**

Routine HIV testing is part of the package of PrEP services. To prevent development of resistance, frequent testing is required for timely identification of PrEP users who become HIV positive. HIV sero-status should be established and documented at the initiation of PrEP, at 1 month and every 3 months after initiation of PrEP. A HIV test should also be done whenever there are symptoms of acute HIV infection. HIV self-testing, HIVST, (as recommended in the national HTS guidelines) may be used to further increase access to HIV testing.

b. **Managing suspected acute seroconversion illness**

Continue PrEP, test for HIV at first contact and after 28 days, and if negative, continue with PrEP and usual follow-up.

c. **Managing Confirmed HIV Infection during PrEP**

- Counsel the patient and urgently link to care and treatment for initiation of full antiretroviral therapy
- Explore with the patient the consistency of PrEP use (assess interruptions and barriers to adherence during PrEP).
- Contact the regional or national TWG. It may be necessary to obtain a baseline VL and DRT to help decide on the optimal first-line ART for the patient.

Improving adherence to PrEP

Approaches to improve adherence include:

d. Encouraging the client to make it a daily habit at any most consistent most convenient time of day linked to an activity done daily such as brushing teeth, taking a meal etc
e. Disclosure of PrEP use to a partner or trusted person
f. Use of reminder devices like a cell phone alarm
g. SMS reminders where available and feasible
h. Explore and mitigate other barriers to adherence
i. Encourage peer support

2. Exercise caution when discontinuing PrEP in a client with HBV infection. Such clients may experience severe flare-up of hepatitis. Refer to hepatitis guidelines and consult a provider experienced in the management of hepatitis.

Table 4.1 Adherence support during follow-up visits

Theme	Adherence message/action
**Climate Setting**	Introduce yourself to the client, giving your name and role, ensure adequate privacy and reassure on confidentiality
**Assess**	• Understanding and experience with adherence: dosage and timing<br>• Experience with possible side effects<br>• Risk reduction efforts since last visit<br>• Challenges to adherence and risk reduction<br>• Possible acute seroconversion illness[1]
**Advice**	• In case of problems with adherence, explore approaches to improving adherence<br>• Emphasize need for adherence and ongoing risk reduction including consistent use of condoms to prevent STIs and pregnancy.<br>• For People Who Inject Drugs refer to a Needle and Syringe Exchange Program and Methadone Assisted Therapy<br>• Remind clients circumstances under which PrEP can be discontinued
**Agree**	• Adherence and risk reduction goals based on degree of the client's desire to meet these goals
**Assist**	• Provide client with any reading material, and if available access to telephone consultation
**Arrange**	• Schedule next counselling/refill appointment date

Assessing for medication side effects

a. *Minor side effects* - few people may experience minor side effects like diarrhoea, nausea, decreased appetite, abdominal cramping or flatulence; dizziness or headaches. Such side-effects are usually mild and resolve without stopping PrEP. If necessary, symptomatic treatment such as anti-diarrhoeal, antiemetic or anti-flatulence medication can be prescribed for a brief period.

b. *Elevated creatinine* - where available, serum creatinine should be estimated at baseline and annually (earlier if the patient is at risk of renal disease). Self-limiting mild creatinine elevation occurs in a few individuals. Risk factors for significant creatinine elevation include:
   - Conditions such as diabetes mellitus and hypertension
   - Age> 60 years of age (in the African population, the risk increases from age >45 years)
   - Reduced CrCl (< 90 ml/min) at baseline
   - Concurrent use of nephrotoxic agents such as NSAIDs

   o If the creatinine clearance (CrCl) is < 50 ml/min, discontinue PrEP immediately and counsel on other HIV preventive measures; refer for further assessment. If the CrCl > 50 ml/min, PrEP may be restarted and creatinine re-assessed after 1 month. Exclude treatable/preventable causes of elevated creatinine such as dehydration, herbal remedies and supplements, NSAID use/abuse, other medications, uncontrolled blood pressure etc.

   The formula for calculating estimated creatinine clearance is provided in the information box below.

Cockcroft–Gault equation:

Estimated Cr Clearance = Sex * ((140 - Age) / (Serum Creat)) * (Weight / 72)

Notes:

- For "sex", use 1 for a male, 0.85 for a female
- Give "age" in years
- Provide "serum creatinine" in mg/dL
- Give "weight" in kilograms (should be lean body weight)

Discontinuing PrEP

Indications for discontinuing PrEP include;

- The client becoming HIV positive, counsel and link to care and treatment. The patient on the recommended first-line ART regimen (refer to the national ART guidelines)
  If there is high likelihood of transmitted HIV drug resistance, refer to the national or regional TWG. Contact NASCOP at ulizanascop@gmail.com for possible VL and DRT
- Change in risk status (low risk)
- Renal dysfunction with creatinine clearance below 50mL/min
- Client request to stop
- Sustained non-adherence
- Sustained viral suppression of the HIV positive partner in a discordant relationship. However, advise the couple to continue using condoms consistently.

PrEP use can be discontinued at least 28 days from the last high risk exposure to HIV.

Restarting PrEP

- A client who stops PrEP for more than 7 days and wishes to restart should be assessed for resumption of PrEP as a new client. Importantly, obtain a HIV test. If a high risk exposure occurred in the previous 7 days (i.e acute HIV infection is suspected), defer PrEP and obtain repeat HIV test after 30 days; if negative, PrEP can be prescribed if the other criteria are fulfilled.

For a sero-discordant couple, the indications for re-starting PrEP by the negative partner include

- HIV positive partner stops taking ART including defaulting from treatment
- Rebound in viral load in the HIV positive partner. Asses for and support adherence, evaluate for treatment failure. Provide the full package of care and support for discordant couples (including PrEP until the partner on ART achieves viral suppression)
- Having a new sexual partner of unknown HIV status.
- Negative partner is assessed to have additional risk of HIV infection such as a new STI
- During pregnancy (for the HIV negative female partner)

PrEP in Special Circumstances

a. Chronic HBV infection

TDF and FTC (as used for PrEP) are also effective in the treatment of HBV infection. HBV infection is not a contraindication to PrEP use. However, due to the risk of hepatitis flare-up after discontinuation of PrEP, exercise caution

when discontinuing TDF/FTC especially in the first 1-3 months after stopping PrEP. Monitor clinical symptoms (nausea, anorexia, jaundice, abdominal pain and dark urine); obtain ALT where available and refer to a physician for specialised assessment and treatment.

b. Pregnancy/Breastfeeding

- For women at substantial ongoing risk of HIV infection who become pregnant or desire to conceive, PrEP decreases the risk of acute HIV infection during pregnancy. Acute HIV infection significantly increases the risk of mother-to-child transmission.
- There's no evidence that TDF/FTC or 3TC increase the risk of birth defects if used during any gestation of pregnancy. However, there's enough evidence to completely exclude harm.
- Assess for pregnancy intention in all women of reproductive age who are considering PrEP and provide counselling on safer conception options including the use of PrEP. Offer effective contraception unless pregnancy is desired.
- Pregnancy and breastfeeding are not contraindications to PrEP. The benefits and potential harm of PrEP should be discussed with the client and the decision to continue PrEP individualized based on ongoing risk for HIV infection during pregnancy.
- Risk reduction counselling should be intensified for an uninfected individual who becomes pregnant while taking PrEP.
- Once the decision to continue PrEP is made, the client should start antenatal care immediately and followed up monthly until cessation of breastfeeding; with care coordinated between the antenatal and PrEP providers.

c. PrEP use in discordance (the illustrations below were provided by….)

- The circumstances for use of PrEP in a discordant relationship include the following
  - PrEP can be offered routinely, to the HIV negative partner, at initiation of ART for the HIV positive partner and continued until viral suppression is achieved.

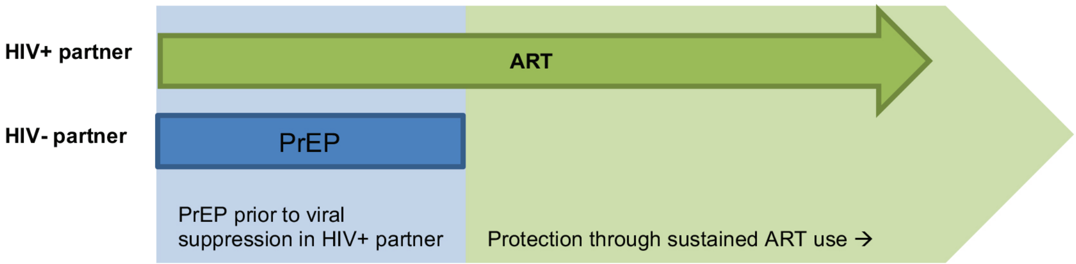

- PrEP can be offered to the HIV negative partner if ART for the HIV positive partner is delayed or declined. In such cases, PrEP is continued until effective ART is provided to the HIV positive partner and viral suppression achieved.

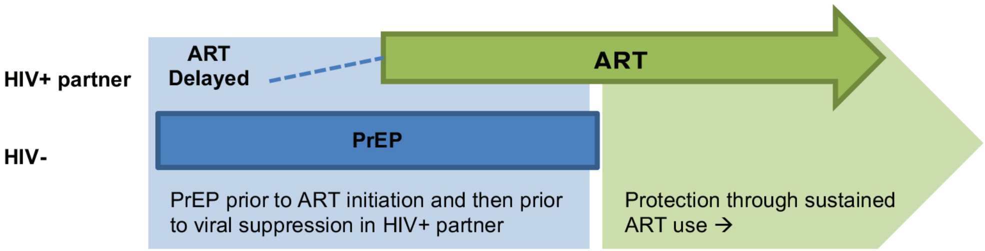

Section 5 Appendices

Appendix 1: Rapid Screening tool

MINISTRY OF HEALTH
NATIONAL AIDS & STIs CONTROL PROGRAM

PrEP Rapid Assessment Screening Tool (RAST)

Age: ________________ **Sex:** ________________ **Date:** __________________

1. What is your HIV status? ***(if response is positive discontinue assessment else administer all questions)***

   ☐Negative ☐Positive ☐Unknown ☐Unwilling to disclose
2. What is the HIV status of your sexual partner(s)?

   ☐Negative ☐Positive ☐Unknown

In the past 6 months

3. Have you had sex without a condom with a partner(s) of unknown or positive HIV status?

   ☐No ☐Yes
4. Have you engaged in sex in exchange of money or other favors?

   ☐No ☐Yes
5. Have you been diagnosed with or treated for an STI?

   ☐ No ☐ Yes
6. Have you shared needles while engaging in intravenous drug use?

   ☐No ☐Yes
7. Have you been forced to have sex against your will or physically assaulted including assault by your sexual partner(s)?

   ☐No ☐Yes
8. Have you used post exposure prophylaxis (PEP) two times or more?

   ☐No ☐Yes

Refer the client for further PrEP assessment at the health facility If:

HIV status of the sexual partner(s) is Positive or Unknown

Any ***Yes*** to the screening questions

Remarks

____

____

____

Appendix 2: Clinic Encounter Record: Initiation and Follow up

MINISTRY OF HEALTH

File no:

Clinical Encounter Record: Oral Pre-Exposure Prophylaxis (PrEP)

Name of facility: ______________ Delivery Point: ______________ Tier: ________ MFL code: __ __ __ __ __
County: ______________ Sub county: ______________ Ward: ______________

Client Profile

Unique client record number: __ __ __ __ __ - __ __ - __ __ __ __ __ Initial visit date: dd / mm / yyyy

Name: First __________ Middle __________ Last __________ Telephone no: __________

Alien/National ID/passport/Birth Cert No: __________ NHIF No: __________ County of Birth __________ Mother Maiden Name __________

Sex: ☐ Male ☐ Female Date of birth: dd / mm / yyyy Age (years): __ __ If age <19, attends school: ☐ Yes ☐ No

Marital status (*select one*): ☐ Never married ☐ Cohabiting ☐ Married monogamous ☐ Married polygamous ☐ Separated/divorced ☐ Widowed

Population Type: ☐ Discordant couple ☐ Fisher Folk ☐ Gen Population ☐ Key Population (Specify) → ☐ MSM ☐ MSW ☐ FSW ☐ PWID

Entry Point & Transfer Status

Referred from (*select one*):
☐ HBTC ☐ VCT site ☐ OPD ☐ MCH ☐ TB clinic ☐ IPD ☐ CCC
☐ Peer ☐ Outreach ☐ Self-referral ☐ Community ☐ Other: __________

If transferred in:
PrEP start date: dd / mm / yyyy Regimen: ☐ TDF-FTC ☐ TDF ☐ TDF-3TC
Facility transferred from: ________ MFL code: __ __ __ __ __ County: ________

Baseline Assessment

Behaviour risk assessment

Mark all that apply:
- ☐ Sex partner(s) is HIV+ and (*mark all that apply*):
  - ☐ Not on ART
  - ☐ On ART <6 months
  - ☐ Suspected poor adherence to ART
  - ☐ Detectable HIV viral load
  - ☐ Couple is trying to conceive
- ☐ Sex partner(s) high risk & HIV status is unknown
- ☐ Has sex with >1 partner
- ☐ Ongoing IPV/GBV
- ☐ Transactional sex
- ☐ Recent STI
- ☐ Recurrent use of post-exposure prophylaxis (PEP)
- ☐ Recurrent sex under influence of alcohol/recreational drugs
- ☐ Inconsistent or no condom use
- ☐ Injection drug use with shared needles and/or syringes

(If yes to any) →

Complete section if sex partner is HIV+

HIV+ partner CCC number: __ __ __ __ ____/__ __ __ __ __
or ☐ NA (not enrolled at a CCC)
or ☐ CCC number/enrollment status unknown

HIV+ partner ART start date: dd / mm / yyyy
or ☐ not on ART at initial visit

Time known to be HIV-serodiscordant: __ __ years + __ __ months

Sex without a condom with HIV+ partner in past 30 days: ☐ Yes ☐ No

Number of living children with HIV+ partner: __ __

Medical assessment & fertility intentions

HIV test results: ☐ Positive ☐ Negative
Blood pressure (mm Hg): __ __ __ / __ __ __
Weight (kg): __ __ __ Height (cm): __ __ __
Signs/symptoms of STI : ☐ Yes; Use codes provided: __________ ☐ No

Chronic illnesses & comorbidities			Treatment
Liver disease:	☐ Yes	☐ No	
Kidney disease:	☐ Yes	☐ No	
1. Other description			
2. Other description			

Male only:
Circumcised: ☐ Yes ☐ No ☐ Unknown

Female only:
LMP: dd / mm / yyyy
Pregnant: ☐ Yes ☐ No
If pregnant: ☐ Planned ☐ Unplanned
Breastfeeding: ☐ Yes ☐ No
On family planning: ☐ Yes ☐ No FP methods: ________
Plan to have children (*select one*):
☐ Trying to conceive ☐ Future ☐ No ☐ Don't know

Clinical notes:

PrEP initiation

Lab results (*Investigations should not delay PrEP initiation. To be recorded when available.*)

Test	Result	Additional steps
Hepatitis B (HBsAg)	☐ Positive ☐ Negative ☐ Not done	If negative, vaccine series initiated: ☐ Yes ☐ No
Hepatitis C	☐ Positive ☐ Negative ☐ Not done	
Serum creatinine	__ __ __ (μmol/L) or ☐ Not done	If done, CrCl (mL/min): __ __ __ *If creatinine is out of range, or CrCl<50 mL/min, refer for further assessment.*

Previous PrEP use: ☐ Yes ☐ No Condom Issued: ☐ Yes ☐ No
Willing to start PrEP: ☐ Yes ☐ No Adherence Counseling Done: ☐ Yes ☐ No
If not willing, reason (*mark all that apply*): ☐ None ☐ Side effects (ADR) ☐ Stigma ☐ Pill burden ☐ Taking pills for a long time ☐ Too many HIV tests

Signs/symptoms of acute HIV: ☐ Yes **☐ No**
Medically ineligible to start PrEP: ☐ Yes **☐ No**
Contraindications for TDF-FTC /TDF-3TC/TDF: ☐ Yes **☐ No**

} **Eligible for PrEP** →

Prescribed PrEP at initial visit: ☐ **Yes** ☐ **No**
Regimen: ☐ TDF-FTC ☐ TDF ☐ TDF-3TC
# of months: __ __
Date of initiation: dd / mm / yyyy

Next appointment date: dd / mm / yyyy **Clinician initials:**

Follow Up Visit

Unique client record number: __ __ __ __ __ __-__ __-__ __ __ __ __ **Name of client**:__________________

Visit date: dd / mm / yyyy
Visit type: ☐ scheduled ☐ unscheduled

Medical assessment & fertility intentions

Clinical notes

Summary of findings

Blood pressure	__ __ __ / __ __ __ mm Hg
Weight	__ __ __ kg
Signs/symptoms of STI(s)	☐ yes ☐ no If yes Use codes provided______
Signs/symptoms of acute HIV	☐ yes ☐ no
If male, circumcised since last visit	☐ yes ☐ no ☐ na (already circumcised)

Possible adverse drug reaction

1	Description
	**Severity** ☐ mild ☐ moderate ☐ severe
	Action (*mark all that apply*) ☐ stop ☐ switched regimen ☐ Other __________
2	Description
	**Severity** ☐ mild ☐ moderate ☐ severe
	Action (*mark all that apply*) ☐ stop ☐ switched regimen ☐ Other __________

Chronic illnesses & comorbidities	Treatment
Liver disease ☐ Yes ☐ No	
Kidney disease ☐ Yes ☐ No	
1 Other description	
2 Other description	

Plan to have children: ☐ trying to conceive ☐ future ☐ no ☐ don't know ☐ client/partner is pregnant

If female:
- LMP: ______________ Pregnant ☐ yes ☐ no
- Breastfeeding ☐ yes ☐ no
- On family planning ☐ none or methods (Indicate the code):________________
- If ended pregnancy since last visit
  - *Outcome date* dd / mm / yyyy
  - *Outcome* ☐ term live ☐ preterm live ☐ induced abortion ☐ *loss*
  - Birth defect(s) ☐ yes ☐ no ☐ don't know

Behaviour risk assessment

Mark all that apply

☐ Sex partner(s) is HIV+ and:
- ☐ not on ART
- ☐ <6 months ART use
- ☐ poor adherence to ART
- ☐ detectable HIV viral load
- ☐ couple is trying to conceive

☐ Sex partner(s) at high risk for HIV & HIV status unknown
☐ Has sex with >1 partner
☐ Ongoing IPV/GBV
☐ Transactional sex
☐ Recent STI

☐ Recurrent use of PEP
☐ Recurrent sex under influence of alcohol/recreational drugs
☐ Inconsistent or no condom use
☐ IDU with shared needles/syringes

Follow up laboratory investigations

HIV test	☐ positive ☐ negative ☐ not done	*If positive, collect sample for drug resistance, and link to CCC (add CCC number)*
Serum creatinine (as per guidelines)	__ __ __ __ µmol/L or ☐ not done	*If creatinine is out of range, or CrCl <50 mL/min, refer for further assessment*
If creatinine done, CrCl ≥50 mL/min	☐ yes ☐ no	
Other (*write in test, results & units [if applicable]*)	1	
	2	

PrEP

Self-assessment of adherence since last visit	☐ Good ☐ Fair ☐ Bad ☐ N/A (did not pick up PrEP at last visit)
If unsatisfactory, reason(s) (*mark all that apply*)	☐ forgot ☐ lost/out of pills ☐ separated from HIV+ partner ☐ no perceived risk ☐ side effects ☐ sick<br>☐ stigma ☐ pill burden ☐ shared with others ☐ none ☐ other ______________
Adherence Counseling done	☐ yes ☐ no **Condoms issued**: ☐ yes ☐ no
PrEP status	☐ continue ☐ restart ☐ discontinue
Prescribed PrEP today	☐ yes ☐ no
If yes, regimen and duration	regimen ☐ TDF-FTC ☐ TDF ☐ TDF-3TC number of months __ __
If discontinued, reason(s) (*mark all that apply*)	☐ HIV test is positive ☐ low risk of HIV ☐ renal dysfunction ☐ client request ☐ not adherent to PrEP<br>☐ viral suppression of HIV+ partner ☐ too many HIV tests ☐ other ______________________

Next appointment date: dd / mm / yyyy
Clinician initials:

Adherence

Adherence:
Good: Missed 0-3 doses per week in the past 1 month
Fair: Missed 4-5 doses per week in the past 1 month
Bad: Missed 6-7 doses per week in the past 1 month
Serum creatinine clearance:
= Sex * ((140 - Age) / (Serum Creat)) * (Weight / 72)
Notes:
- For "sex", use 1 for a male, 0.85 for a female
- Give "age" in years
- Provide "serum creatinine" in mg/dL
- Give "weight" in kilograms (should be lean body weight)

FP Methods:
C = Condoms
TL = Tubal ligation/female sterilization
FA = Fertility awareness method/periodic abstinence
D = Diaphragm/cervical cap
LAM = Lactational Amenorrhea Method
IUD = Intra uterine device
IMP = Implant
INJ = Injectable
OC = oral contraceptive pills
ECP = Emergency contraceptive pills dispensed
V = Vasectomy (partner's)

STI Diagnosis:
Genital Ulcer Disease (GUD), Vaginitis and/or Vaginal Discharge (VG),
Cervicitis and/or Cervical Discharge (CD),
Pelvic Inflammatory Disease (PID),
Urethral Discharge (UD),
Anal Discharge (AD),
Others (O)

Appendix 3: PrEP Register

Facility Name ______										Delivery point:......						Month						Year......			
										Medical Assessment						HIV Risk assessment and PrEP adherence						Yearly Creatinine & HBsAg tests		For those who turn HIV Positive while on PrEP	
[illegible]	[illegible]	[illegible]	[illegible]	[illegible]	[illegible]	[illegible]	[illegible]	[illegible]	[illegible]	[illegible]	[illegible]	[illegible]	STI signs and symptoms (Yes/No)	Creatinine clearance done (Yes/No)	HBsAg [illegible]	Risk of HIV Infection		Adherence	Adherence Counselling	PrEP Status		Creatinine Done(Yes/No)	HBsAg [illegible]	Linkage to CCC (Yes/No)	[illegible]
													[illegible]	[illegible]		[illegible]	[illegible]	[illegible]	[illegible]	[illegible]	[illegible]	[illegible]		[illegible]	

Indicator	Age								
	Male				Female				
	15 - 19	20-24	25-29	30 & above	15 - 19	20-24	25-29	30 & above	Total
Number screened for HIV risk									
Number eligible for PrEP									
Number initiated (New) on PrEP									
Number continuing (Refill) on PrEP									
Number restarting (Restart) PrEP									
Number Current on PrEP (New+ Refill+ Restart)									
Number tested HIV positive while on PrEP									
Number diagnosed with STI									
Number discontinued PrEP									

Appendix 4: PrEP Summary Reporting Tool

NATIONAL AIDS & STI PROGRAMME

PrEP Summary Reporting Tool

Site Name/Facility:____________________ MFL-Code: ____________________

Sub-County: ____________________ County: ____________________

Reporting Month: ____________________ Year____________________

**1. Number Assessed For HIV risk**	
1.1 Males 15 - 19 Years	
1.2 Females 15 - 19 Years	
1.3 Males 20 - 24 Years	
1.4 Females 20 - 24 Years	
1.5 Males 25 - 29 Years	
1.6 Females 25 – 29 Years	
1.7 Males 30 Years and older	
1.8 Females 30 Years and older	
**Total**	

**2. Number Eligible for PrEP**	
2.1 Males 15 - 19 Years	
2.2 Females 15 - 19 Years	
2.3 Males 20 - 24 Years	
2.4 Females 20 - 24 Years	
2.5 Males 25 - 29 Years	
2.6 Females 25 - 29Years	
2.7 Males 30 Years and older	
2.8 Females 30 Years and older	
**Total**	

**3. Number initiated (New) on PrEP**	
3.1 Males 15 - 19 Years	
3.2 Females 15 - 19 Years	
3.3 Males 20 - 24 Years	
3.4 Females 20 - 24 Years	
3.5 Males 25 - 29 Years	
3.6 Females 25 - 29 Years	
3.7 Males 30 Years and older	
3.8 Females 30 Years and older	
**Total**	

**4. Number continuing (Refills) on PrEP**	
4.1 Males 15 - 19 Years	
4.2 Females 15 - 19 Years	
4.3 Males 20 - 24 Years	
4.4 Females 20 - 24 Years	
4.5 Males 25 - 29 Years	
4.6 Females 25 - 29 Years	
4.7 Males 30 Years and older	
4.8 Females 30 Years and older	
**Total**	

**5. Number Restarting PrEP**	
5.1 Males 15 - 19 Years	
5.2 Females 15 - 19 Years	
5.3 Males 20 - 24 Years	
5.4 Females 20 - 24 Years	
5.5 Males 25 - 29 Years	
5.6 Females 25 - 29 Years	
5.7 Males 30 Years and older	
5.8 Females 30 Years and older	
**Total**	

**6. Number currently on PrEP (New + Refill+ Restart)**	
6.1 Males 15 - 19 Years	
6.2 Females 15 - 19 Years	
6.3 Males 20 - 24 Years	
6.4 Females 20 - 24 Years	
6.5 Males 25 - 29 Years	
6.6 Females 25 - 29 Years	
6.7 Males 30 Years and older	
6.8 Females 30 Years and older	
**Total**	

**7. Number tested HIV positive while on PrEP**	
7.1 Males 15 - 19 Years	
7.2 Females 15 - 19 Years	
7.3 Males 20 - 24 Years	
7.4 Females 20 - 24 Years	
7.5 Males 25 - 29 Years	
7.6 Females 25 - 29 Years	
7.7 Males 30 Years and older	
7.8 Females 30 Years and older	
**Total**	

**8. Number diagnosed with STI**	
8.1 Males 15 - 19 Years	
8.2 Females 15 - 19 Years	
8.3 Males 20 - 24 Years	
8.4 Females 20 - 24 Years	
8.5 Males 25 - 29 Years	
8.6 Females 25 - 29 Years	
8.7 Males 30 Years and older	
8.8 Females 30 Years and older	
**Total**	

**9. Number discontinued PrEP**	
9.1 Males 15 - 19 Years	
9.2 Females 15 - 19 Years	
9.3 Males 20 - 24 Years	
9.4 Females 20 - 24 Years	
9.5 Males 25 - 29 Years	
9.6 Females 25 - 29 Years	
9.7 Males 30 Years and older	
9.8 Females 30 Years and older	
**Total**	

Appendix 5: Frequently Asked Questions about Pre-Exposure Prophylaxis

What is PrEP?

PrEP stands for Pre-Exposure Prophylaxis. PrEP is anti-HIV medication taken by HIV negative people who are at high risk of HIV infection to reduce their chances of becoming infected.

How is PrEP different from regular ARV drugs?

PrEP is oral ARV medication used for HIV negative people for HIV prevention. However, the same ARV medication can be used by HIV positive people in combination with additional ARV drugs.

How is PrEP (Pre Exposure Prophylaxis) different from Post-Exposure Prophylaxis (PEP)?

Even though PrEP and PEP are both taken by HIV negative people to prevent HIV infection, they are different. PrEP is used by HIV negative people who are at ongoing risk of HIV **before** exposure to reduce their chances of getting HIV. PEP is used by HIV negative people **after** a possible exposure to HIV but must be taken within 72 hours.

How does PrEP work?

When a person is exposed to HIV through blood, sexual intercourse or coming into contact with infected body fluid, PrEP significantly reduces the chances of being infected with the HIV by killing the virus before it establishes infection.

What are the benefits of PrEP?

PrEP can help people who are HIV-negative with ongoing risk of HIV infection to remain HIV negative. It is more effective when combined with other prevention methods such as condoms.

PrEP offers

- Decreased anxiety
- Increased communication, disclosure, trust
- Increased self-efficacy

Among HIV discordant couples, PrEP is a means to

- Reduce risk of HIV transmission
- Meet their fertility desires
- Cope with HIV sero-discordance.

When can I use PrEP?

Any person who is at high risk for acquiring HIV, and meets ANY of the following indications;

- Has a sexual partner who is known HIV positive and either: not on ART, has not been on ART for 6 months, Suspected of poor adherence to ART, or who has not achieved viral suppression.
- Sexual partner(s) are of unknown HIV status and are at high-risk for HIV infection i.e. has multiple sexual partners, has had STIs, engages in transactional sex, injects drugs
- Engaging in transactional sex (sex in exchange of gifts etc.)
- History of recent sexually transmitted infection
- Recurrent use of post-exposure prophylaxis (PEP)
- History of sex while under the influence of alcohol or recreational drugs as a habit
- Inconsistent or no condom use or unable to negotiate condom use during intercourse with persons of unknown HIV status
- Injecting drug use where needles and syringes are shared
- A discordant couples (where one partner is infected with HIV and the other is not) who are trying to conceive

Can I use PrEp with other medicines?

- It is important to seek doctors' advice on which medicines one can use together with PrEP

When should I not use PrEP?

- You should not use PrEP if:
  - If you are HIV positive
  - If you do not know their HIV status
  - If you cannot use your PrEP pill daily
  - If you have been advised by a health care provider not to use PrEP

Should I use PEP if I suspect that am exposed to HIV when taking PrEP?
Ideally, if you are taking PrEP every daily as prescribed, you do not need to use PEP because PrEP already provides a high degree of protection from any potential HIV exposure. Continue taking your PrEP pill and discuss with your healthcare provider if you are concerned about possible HIV infection

What are the side effects of PrEP?
Some people who take PrEP experience side effects that last for a short period. These may include headache, weight loss, nausea, vomiting, and abdominal discomfort and often reduce or stop after a few weeks of taking the PrEP. Inform your provider about any discomfort that persists or if you are concerned about how you feel after starting PrEP.

How should I take PrEP Pills?
The PrEP Pill should be taken once a day for as long as a person remains at risk of HIV infection (or as advised by the Health care provider). You should not take 2 pills at the same time or on the same day to make up for a missed dose.

Can I still use condoms when taking PrEP?

PrEP does not protect users from STI or pregnancy. PrEP is provided as part of combination prevention including condom use, VMMC, risk reduction counselling and support etc.

Does PrEP contribute to increase in risky sexual behavior?

PrEP is provided part of a package of combination prevention including risk reduction counselling and support. Provided this way, PrEP does not contribute to behavioural disinhibition and risk taking.

Am I protected from HIV if I miss a PrEP pill or pills?
When you miss one or more pills, you greatly reduce the ability of the PrEP to provide you with full protection against HIV infection. Evidence has showed that PrEP provides the best protection from HIV if it is taken consistently every day.

Can I share PrEP with others?
PrEP should only be taken by the person prescribed and should not be shared with others. Everyone who wants to use PrEP should discuss the intention with a health provider.

How long can I take PrEP?
Someone can take PrEP for as long as they remain at risk of HIV infection. However it is important to continue consulting a health provider for advice.

Can I use PrEP along with other medicines?
It is important to seek doctors' advice on which medicines one can use together with PrEP

When should I stop/discontinue taking PrEP?
You should stop/ discontinue PrEP if you meet ANY of the following criteria are met:

- HIV positive
- If you reduce your risk for getting infected with HIV

- If the health care provider informs you that your kidney (Renal) function is low after doing some test
- If you request to stop
- If you are not adhering to the drugs well
- If you are in a discordant relationship and your HIV positive partner has achieved sustained viral suppression. But you should continue to consistently use condom

Can a pregnant woman take PrEP? What happens if a woman who is taking PrEP becomes pregnant?
Yes, if you are pregnant or intending to get pregnant and your partner is HIV positive, you can take PrEP.

Can One Develop Resistance to PrEP

- Resistance occurs sometimes when antiretroviral agents are used for treatment.
- Extremely rare with PrEP, and limited to those with unrecognized acute HIV infection when starting PrEP.
  - Resistance can only occur if there is continued PrEP use in the background of unrecognized HIV infection.
- The benefits of PrEP far outweigh the risk and concerns about drug resistance.

Appendix 6: List of Contributors and Reviewers

Names of Contributors	Organization
Abraham Rono	HEALTH RIGHT INTERNATIONAL
Alice Karanja	JHPIEGO
Alita Monroe wise	UNIVERSITY OF WASHINGTON
Anabel Gomez	AVAC
Anangwe M. Samson	DOD
Ann Njoki	NASCOP/MOH
Anthony Gichangi	JHPIEGO
Beth Mbugua	NASCOP/MOH
Brenda Bakobye	NEPHAK
Brian Wakhutu	JHPIEGO
Christine Akinyi	LVCT HEALTH
Daniel Were	JHPIEGO
Dicson Mwakangah	CDC
Dorcus Abuya	NHRL
Dorothy Mwagae	NASCOP/MOH
Dr Lilly Nyaga	NASCOP/MOH
Dr Martin Sirengo	NASCOP/MOH
Dr Micah Anyona	JHPIEGO
Dr Nelly Mugo	KEMRI
Dr Sarah Masyuko	NASCOP/MOH
Dr. Bill Sinkele	SAPTA
Dr. Bob Agwata	NASCOP/MOH
Dr. Elizabeth Irungu	PARTNERS IN HEALTH AND DEVELOPMENT(PHRD)
Dr. Godwin Mugo	CHAK
Dr. Henry Tabifor, ,	UNAIDS
Dr. Jared Mecha	UON/KNH
Dr. Josphat Kosgei	KEMRI /WRP
Dr. Michelle Ogolla	GSK
Dr. Misiko Linda	NASCOP/MOH
Dr. Mugwanya Kenneth	PARTNERS IN HEALTH AND DEVELOPMENT(PHRD)
Dr. Omwoyo Willis	MOH
Dr. Peter Cherutich	MOH
Dr. Stellah Bosire	KMA
Dr. Ulrike Gilbert	UNICEF
Dr. Urbanus Kioko	AVENIR HEALTH
Dr. Ahmed Sheikh Hussein	MOH
Dr. Barbara Mambo	NASCOP/MOH
Dr. Brian Chirombo	WHO
Dr. Evans Imbuki	NASCOP/MOH
Dr. Irene Inwani	KNH
Dr. Jebet.K. Boit	MATHARI NTRH
Dr. John Kinuthia	KNH
Dr. Joyce Wamicwe	NASCOP/MOH
Dr. Maureen Kimani	NASCOP/MOH

Dr. Muthoni Karanja	NASCOP/MOH
Dr. Rose Wafula	NASCOP/MOH
Dr. Teresa Alwar,	UNICEF
Edward Omondi	NASCOP/MOH
Elizabeth Kubo	UON
Emma Mwamburi	USAID
Eunice Mutisya	PS KENYA
Eunice Njeri	NASCOP/MOH
Fatuma Mohamed	NASCOP/MOH
Fredrick Maweu	LVCT HEALTH
Fredrick Otieno	NRHS
Geoffrey Njenga	JHPIEGO
George Victor Owino	KAVI
Gloria Gakii	SWOP/UON
Godfrey Odhiambo	JHPIEGO
Harriet Kongon	UNAIDS
Jacque Wambui	NEPHAK
Jane Mutegi	JHPIEGO
James Kabiro	ORDER PHARMACY
Japheth Gituku	NASCOP/MOH
Japheth Nyambane	NASCOP/MOH
Jason Reed	JHPIEGO
Jeremia Hongo	PS KENYA
Jill Peterson	FHI360/GEMS
Joab Khasewa	NACC
Joel Mutinda	NASCOP/MOH
Johnson Birgen	SAUTI SKIKA
Jordan Kyongo	LVCT HEALTH
Joshua Kimani	UOM SWOP
Judy Mwangi	JSI
Justus Oganda	CHAI
Kennedy Murithi	CHAI
Lenet M. Bundi	NASCOP/MOH
Lucy Maikweki	PS KENYA
Mary Mugambi	NASCOP/MOH
Mercy Kamau	JHPIEGO
Michael Macharia	NASCOP/MOH
Mohamud Mohamed	NASCOP/MOH
Nancy Bowen	NHRL
Naomi Siele	NASCOP/MOH
Nicholas Ngugi	UNAIDS
Norton Sang	SEARCH
Obwiri Kenyatta	EGPAF
Olivia Njathi	CHAI
Pacific Akinyi	NASCOP/MOH
Parinita Bhattacharjee	NASCOP/MOH
Patricia K. Macharia	NASCOP/MOH
Patricia Oluoch	CDC

Patriciah Jeckonia	LVCT HEALTH
Patrick Mutua	NASCOP/MOH
Paul Ereng	MSF FRANCE
Pauline Mwololo	NASCOP/MOH
Peter Michira	PARTNERS IN HEALTH AND DEVELOPMENT(PHRD)
Peter Njane	ISHTAR
Philip Muchiri	CHAI
Precious Otieno	NASCOP/MOH
Prince N Bahati	IAVI
Prince N bahati	IAVI
Prof Bukusi Elizabeth	KEMRI
Roseline Warutere	NASCOP/MOH
Sarah Goretti	WRP
Serah Maloba	NASCOP/MOH
Soud Tengah	JHPIEGO
Stephen Wanjala	MSF FRANCE
Timothy Nzomo	NHRL
Tom Marwa	JHPIEGO
Urvi Parikh	UNIVERSITY OF PITTSBURGH/GEMS
Victor Waweru	ISHTAR
Violet Otindo	NASCOP/MOH
Wcyliff Lihanda	IRC
Winnie Owiti	NASCOP/MOH

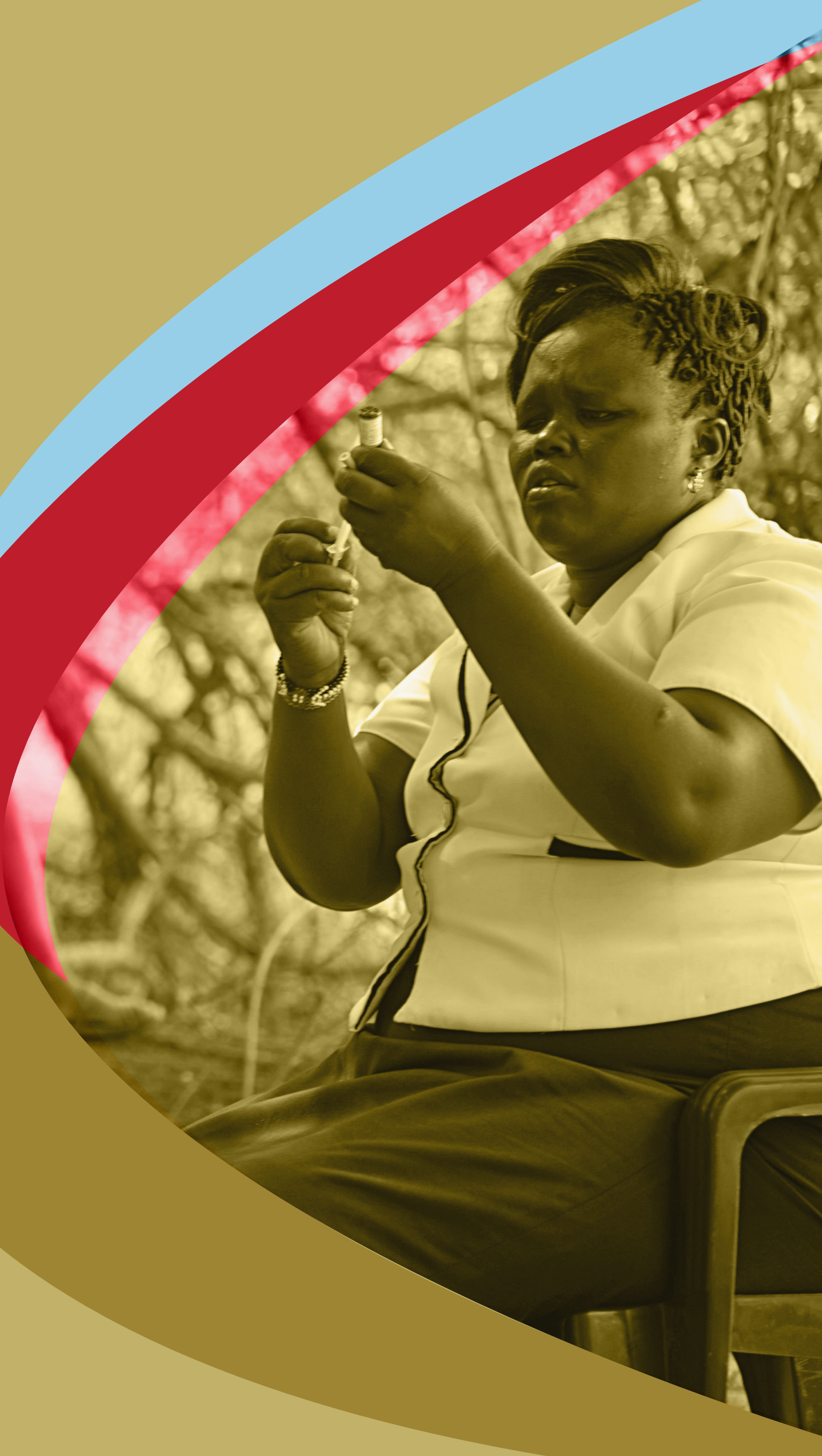

Supplement: S3 Text — (PDF) [file pgph.0000092.s003.pdf]
