# Supplementary material for: Using an on-site modular training approach to amplify prep service delivery in public health facilities in Kenya
Source: PLOS Glob Public Health. 2022 Mar 10;2(3):e0000092. doi: 10.1371/journal.pgph.0000092 (PMC10021257; doi:10.1371/journal.pgph.0000092)
Supplement: S4 Text — (PDF) [file pgph.0000092.s004.pdf]

**Pre Exposure Prophylaxis Pre and Post Test**

(Circle only ONE response)

1. Which of the following is not true about PrEP?
    a. It is taken daily
    b. A person needs to take 7 doses before it is effective
    c. A person needs to be confirmed HIV negative
    d. Must be taken just before a risky exposure
2. *True or False:* PrEP is safe for women to use in pregnancy and breastfeeding.
    a. True
    b. False
3. Which of the following is NOT eligible for PrEP?
    a. Serodiscordant couple, not on ART
    b. Serodiscordant couple, on ART trying to conceive
    c. An HIV-uninfected person with a habit of having sex while taking alcohol
    d. An HIV-uninfected person with signs of acute HIV infection
4. *True or False:* PrEP should be taken by HIV-uninfected in HIV discordant relationships for the rest of their life, regardless of if the HIV-infected person is on ART.
    a. True
    b. False
5. The following is the preferred drug for oral PrEP:
    a. TDF
    b. TDF/3TC
    c. TDF/FTC/EFV
    d. TDF/FTC
6. How often should facilities report and request for oral PrEP products from KEMSA/NASCOP?
    a. Requests through S12 any time facility runs out of commodities
    b. Monthly through CDRR reporting
    c. Quarterly, through the Sub County Pharmacist
    d. Based on availability at KEMSA
    e. Based on client recruitment
7. In pharmacovigilance, the following form is used to report suspected poor-quality medicines
    a. Yellow form

b. Pink form

c. Alert Card

8. Which of the following people is NOT likely to transmit HIV to their HIV-uninfected partner?
    a. A man who has a high CD4 and does not want to take ART
    b. A woman who has been on ART for 1 month
    c. A woman who has been on ART for 5 years and has an undetectable viral load
    d. They all have the same likelihood of transmitting HIV
9. An HIV-uninfected person on PrEP is NOT likely to:
    a. Contract HIV
    b. Get resistance
    c. Get pregnant
    d. Only a & b
    e. All of the above
10. PrEP can help people with the following:
    a. Prevent HIV
    b. Feel more intimate in their relationship
    c. Feel empowered
    d. All of the above
    e. None of the above
11. Which of the following is NOT an advantage of providing couples-based care?
    a. Couples hear messages together, allowing for shared understanding
    b. Care focuses on the HIV-infected partner only
    c. Couples work together to make decisions about treatment and care
    d. Both members of couple can engage in risk reduction and prevention decisions
12. Which of the following is a PrEP reported indicator?
    a) Number diagnosed with STI
    b) Number discontinued PrEP
    c) Number Eligible for PrEP
    d) All the above
13. Which of the following statements is not true about PrEP register?
    a) Used together with PrEP DAR as primary sources for PrEP monthly reporting.
    b) It is a longitudinal register used to record all individuals issued with PrEP

c) Can act as a transfer document for PrEP users

d) Collects personal information/details of PrEP clients

14. Once one is initiated on PrEP it is to be taken forever.

    a) TRUE

    b) FALSE

15. All individuals found to be HIV negative are eligible for PrEP

    a) TRUE

    b) FALSE

16. Below is PrEP eligibility criteria, except?

    a) Client with a HIV Positive partner

    b) Client who is HIV positive

    c) Client having recent STI

    d) Client injecting drug with shared needles

17. Which data element is not captured in the PrEP register?

    a) Adherence counselling

    b) Population type

    c) Client biodata

    d) Next appointment date

18. PrEP is offered to one of the individuals, which one?

    a) Anyone who seeks for PrEP in a health facility

    b) All pregnant women who are HIV positive

    c) Individuals assessed and found to have ongoing substantial risk of contracting HIV

    d) All the above

19. During HIV testing of Allan, a PrEP client at Kanombe County Hospital, the HIV rapid test result was inconclusive. What was the next course of action for the Health care worker?

    a) Discontinue PREP

    b) Refer for PCR

    c) Prepare DBS for drug resistance testing

    d) All of the above
