# Supplementary material for: Using an on-site modular training approach to amplify prep service delivery in public health facilities in Kenya
Source: PLOS Glob Public Health. 2022 Mar 10;2(3):e0000092. doi: 10.1371/journal.pgph.0000092 (PMC10021257; doi:10.1371/journal.pgph.0000092)
Supplement: S5 Text — (PDF) [file pgph.0000092.s005.pdf]

PSUP Modular Training for Mentored Clinics In-Depth Interview Question Guide

Instructions:

The following is a guide. Try to ask all the questions below in the order given, but it is more important to maintain the flow of discussion. Suggested probes have been included. Start with the following introductory script:

****

Hi, my name is ____________. Thank you for agreeing to participate in an interview today. I am interested in understanding your thoughts, experiences and opinions about the PrEP training you received within your facility – we refer to it as Modular Training. I will ask you questions that you are free to answer in any way you wish. Feel free to elaborate on any of your points. If a question is unclear to you, please feel free to ask me to explain it.

I would like to record the discussion so I don't miss anything that you say. I will not include your names on any documents or in the recording. Your answers will be kept confidential, which means we will keep what you say private from others. Is it okay if I record our discussion? [Wait for the participant to give verbal consent to recording]

Before we start, I would like to remind you that there are no wrong answers in this discussion. We are interested in knowing what you think, so please feel free to be open and share your point of view. We hope you can help us understand what did and did not work in the PrEP training so that we can make changes in the future. Your comments about what did not work are just as helpful as your comments about what did work. It is very important that we hear your opinion. You do not have to answer all the questions. If you want to stop the discussion at any time, just let me know.

****

1. Please tell me how long have you been providing PrEP at this facility? How has the experience been?

   Theme 1: PrEP Modular Training

   I would like to hear about the PrEP training you received which was conducted in the health facility

2. How did you learn to deliver PrEP services?
   a. Could you describe how the training happened?
   b. How did the training affect your PrEP delivery? What makes you say so?
   c. What would you say are some of the advantages of getting PrEP training in the health facility? (*probes: number of people trained, time flexibility*)
      i. How would you compare this training to other training sessions that you have attended?
         - Given an opportunity to choose, which mode of training would you prefer? Reasons?
         - How do others who attended the modular trainings feel about it?
      ii. How do you feel about your clinic's ability to host modular training sessions?
   d. What suggestions would you give to improve PrEP training as it was done in your facility?

Theme 2: PrEP Delivery

I would like to hear about how PrEP delivery is happening in the facility

3. Facilitators and challenges of integrating PrEP delivery in this clinic?
    a. Are there things about your clinic that make offering PrEP easy? (*Probes: clinic flow, location of pharmacy, records documentation*)
    b. Are there things about your clinic that make offering PrEP challenging? (*Probes: clinic flow, location of pharmacy, records documentation*)
    c. Are there things in the way PrEP is designed to be delivered that make offering PrEP easy? (*Probes: risk assessment required, HIV test required, frequency of visits*)
    d. Are there things in the way PrEP is designed to be delivered that make offering PrEP challenging? (*Probes: risk assessment required, HIV test required, frequency of visits*)
    e. What are some strategies that might minimize these challenges?
    f. Overall, how accepting were health providers at your clinic about offering PrEP?

4. What has changed with regard to PrEP delivery as compared to before the outbreak?
    a. How has the way this clinic is staffed changed?
    b. Has the facility had any changes (e.g., changes in operating hours, temperature checks for clients, etc?)
    c. How has the way the clinic conducts demand creation for PrEP changed?
    d. Has the way in which clients are initiated on PrEP changed?
    e. How has the way in which PrEP is delivered at follow up and refill visits changed? Have TCAs changed?
    f. How have retention efforts changed?

5. Tell me about who is leading PrEP implementation at your clinic.
    i. What do they do that made the process easier?
    ii. What were challenges?
    iii. Who were the people who helped motivate others?
    iv. How did the leaders in the clinic feel about PrEP happening at their clinic? (*Facility in charges, medical supervisors*)
