# Supplementary material for: Using an on-site modular training approach to amplify prep service delivery in public health facilities in Kenya
Source: PLOS Glob Public Health. 2022 Mar 10;2(3):e0000092. doi: 10.1371/journal.pgph.0000092 (PMC10021257; doi:10.1371/journal.pgph.0000092)
Supplement: S6 Text — (PDF) [file pgph.0000092.s006.pdf]

MODULAR TRAINING SITES AND TRAINING DATES

FACILITY	TRAINING START DATE
AHERO MEDICAL CENTRE	21-Oct-20
AKALA SUB COUNTY HOSPITAL	18-Feb-20
AMBIRA HOSPITAL	15-Oct-19
AWASI CATHOLIC MISSION HOSPITAL	20-Sep-20
AWENDO SUB COUNTY HOSPITAL	7-Oct-19
BAMBURI HEALTH CENTER	15-Sep-20
BARICHO HEALTH CENTRE	12-Aug-20
BORO DISPENSARY	8-Sep-20
BWARE DISPENSARY	4-Feb-20
DEDE HEALTH CENTRE	12-Nov-19
EDARP DOONHOLM	26-Mar-19
GACHORORO HEALTH CENTRE	12-Nov-19
GAKOE GATUNDU NORTH	5-Feb-20
GITHUMU AIC MISSION HOSPITAL	24-Sep-19
GITHUNGURI - BETA CARE HOSPITAL	30-Jan-20
GITHURAI LANGATA HEALTH CENTER	1-Aug-19
GOD KWER HEALTH CENTRE	2-Dec-19
GOT AGULU SUB COUNTY HOSPITAL	29-May-19
HOPE MEDICAL CENTRE	22-Jan-19
IAP MANGU	26-Feb-20
IGEGANIA SUB COUNTY HOSPITAL	19-Feb-20
ISLAND FARM HEALTH CENTER	9-Nov-20
ITHANGA HEALTH CENTRE	23-Oct-19
JKUAT HOSPITAL	1-Oct-20
K-MET HEALTH FACILITY	19-Nov-19
KABONDO SUB COUNTY HOSPITAL	14-Feb-19
KAGUMO HEALTH CENTRE	12-Mar-19
KALIMONI MISSION HOSPITAL	14-Nov-19
KANDARA SUB-COUNTY HOSPITAL	3-Sep-19
KANGAITA HEALTH CENTRE	29-Aug-19
KANGARU HEALTH CENTRE	17-Aug-20
KANGEMA SUB-COUNTY HOSPITAL	11-Sep-19
KARIOBANGI NORTH HEALTH CENTRE	17-Dec-19
KENOL HOSPITAL	21-Nov-19
KIANYAGA SUB COUNTY HOSPITAL	16-Sep-20
KIBERA SOUTH HEALTH CENTER	25-Jul-19
KIGUMO SUB-COUNTY HOSPITAL	10-Dec-19

KIHARA SUB-COUNTY HOSPITAL	16-Jan-20
KIMBIMBI SUB-COUNTY HOSPITAL	3-Sep-20
KIRWARA SUB COUNTY HOSPITAL	2-Oct-19
KOGELO DISPENSARY	6-Oct-20
KOMBEWA COUNTY HOSPITAL	9-Sep-20
KONGOWEA HEALTH CENTER	2-Sep-20
KUTUS HEALTH CENTRE	19-Sep-20
KIGANJO HEALTH CENTRE	22-Oct-20
KUOYO HEALTH CENTRE	11-Aug-20
ST. MARYS MISSION HOSPITAL	22-May-19
LARI LEVEL IV HOSPITAL	25-Feb-20
LIMURU HEALTH CENTRE	19-Nov-19
LUNGA LUNGA HEALTH CENTER	14-Aug-19
MADIANY SUB COUNTY HOSPITAL	5-Nov-19
MAKUYU HEALTH CENTRE	19-Jun-19
MAMA LUCY KIBAKI HOSPITAL	19-Aug-19
MANYWANDA SUB COUNTY HOSPITAL	26-Nov-19
MARAGUA RIDGE HOSPITAL	28-Nov-19
MARIWA DISPENSARY	4-Feb-20
MATATA NURSING HOSPITAL	22-Oct-19
MBITA SUB COUNTY HOSPITAL	8-Dec-20
MIGORI COUNTY HOSPITAL	10-Mar-20
MINYENYA DISPENSARY	25-Apr-19
MUHORONI COUNTY HOSPITAL	3-Apr-19
MUKURWE-INI SUB-COUNTY HOSPITAL	6-Aug-19
MURIRANJAS HOSPITAL	6-May-19
MUTITHI HEALTH CENTRE	24-Sep-20
NARUMORU HEALTH CENTER	18-Nov-20
NGERE DISPENSARY	22-May-19
NJOKIINI HEALTH CENTRE	22-Aug-19
NYAKURU HEALTH CENTRE	17-Mar-20
NYAMARAGA SUB COUNTY HOSPITAL	7-Oct-19
NYANGANDE HEALTH CENTRE	23-Jul-19
NYANGIELA HEALTH CENTRE	3-Sep-19
NYATHUNA SUB-COUNTY HOSPITAL	16-Sep-19
NYERI TOWN HEALTH CENTRE	15-Oct-20
OGONGO SUB COUNTY HOSPITAL	8-Dec-20
OGWEDHI SUB COUNTY HOSPITAL	16-Sep-19
OTHAYA SUB-COUNTY HOSPITAL	25-Jun-19
OTHORO DISPENSARY- MIGORI	15-Oct-19

OUR LADY OF PERPETUAL SUPPORT	21-Oct-20
OYANI SUB COUNTY HOSPITAL	24-Sep-19
RANEN HEALTH CENTER	25-Aug-20
RANGALA MISSION HOSPITAL	3-Mar-20
RIRUTA HEALTH CENTRE	30-Oct-19
RUIRU - GITHUNGURI HEALTH CENTRE	17-Oct-19
RWAMBWA SUB COUNTY HOSPITAL	15-Jul-19
SAGANA SUB COUNTY HOSPITAL	11-Mar-20
SARO DISPENSARY	2-Dec-19
SHIRIKISHO MISSION HOSPITAL	15-Apr-19
SHOFCO KIBERA	21-Aug-19
SONY MEDICAL CENTRE	11-Aug-20
ST JOSEPH’S MISSION HOSPITAL	6-Aug-19
ST BARNABAS HOSPITAL	3-Nov-20
THIBA HEALTH CENTRE	11-Mar-20
TIGONI HOSPITAL	15-Oct-19
TINGANGA HEALTH CENTRE	26-Sep-19
TINGWANGI HEALTH CENTRE	28-Jan-20
UKWALA SUB COUNTY HOSPITAL	21-Aug-19
URIRI SUB COUNTY HOSPITAL	11-Jun-19
USIGU SUB COUNTY HOSPITAL	16-Sep-19
UTHIRU HEALTH CENTRE	8-Oct-19
WANGIGE SUB-COUNTY HOSPITAL	25-Jul-19
WARAZO JET HEALTH CENTRE	9-Nov-20
WIRE HEALTH CENTRE	9-Jul-19
YALA HOSPITAL	26-Jun-19
ZIWA LA NGOMBE HEALTH CENTRE	13-Oct-20
