# Supplementary material for: Using an on-site modular training approach to amplify prep service delivery in public health facilities in Kenya
Source: PLOS Glob Public Health. 2022 Mar 10;2(3):e0000092. doi: 10.1371/journal.pgph.0000092 (PMC10021257; doi:10.1371/journal.pgph.0000092)
Supplement: Attachment — Submitted filename: Response to Reviewers.doc [file pgph.0000092.s007.doc]

**KENYA MEDICAL RESEARCH INSTITUTE**

**Centre for Clinical Research, P.O. Box 20778 - 00202, NAIROBI, Kenya,**

**Tel: (254) (020) 2722541, 2713349, 0722-205901, 0733-400003; Fax (254) 2720030**

**E-mail: ccr@nairobi.mimcom.net; Website:** [**www.kemri.org**](http://www.kemri.org/)

October 25, 2021

Dr. Catherine Kyobutungi and Prof. Madhukar Pai,

Editors-in Chief,

*PLOS Global Public Health.*

Dear Dr. Kyobutungi and Prof. Pai,

Thank you very much for giving us the opportunity to revise our paper “Using an on-site modular training approach to amplify PrEP service delivery in public health facilities in Kenya” (PGPH-D-21-00431) for publication in *PLOS Global Public Health.*

Thank you for the constructive and valuable comments from the reviewers. We have carefully considered revised the manuscript according to the comments, presented point-by-point below. We believe that the manuscript has been much improved owing to the comments. We hope our responses and revisions address the issues satisfactorily. The alterations can be seen with “tracked changes” in the documents.

Thank you for your consideration.

Sincerely,

Elizabeth M. Irungu, MBChB, MPH, PhD

Centre for Clinical Research,

Kenya Medical Research Institute

**Journal Requirements**

1. Please provide the dates of on-site training and a list of facilities where training took place.

We have provided the list of facilities where training took place and the training dates in supplementary material (S6 File)

2. In your Methods section, please provide additional information about the training participants. Please ensure you have provided sufficient details to replicate the analyses such as:
a. the recruitment date range (month and year),

Identification and training of health facilities occurred between January 2019 and December 2020. This is stated in Lines 16 and 168.

b. a description of any inclusion/exclusion criteria that were applied to training participant recruitment,

Providers selected for training were those likely to encounter individuals at risk of HIV and those working in departments that offer HIV treatment and prevention services. We have included a statement that reads “*Health providers working in the facility who were likely to come into contact with persons at risk of acquiring HIV or likely to be involved in provision of PrEP services were eligible for training”* (Lines 89-91)

c. a table of relevant demographic details,

We used the Ministry of Health tools for this project. Participant cadre was the only demographic data available from these tools. These data have been summarized in Lines 171-172

d. a statement as to whether your sample can be considered representative of a larger population.

We have addressed generalizability of our study in the limitations section of Discussion. We have a statement that reads “*The participating facilities were from central and western regions of Kenya, nevertheless they reflect the spectrum of public health facilities in Kenya, making the lessons learned generalizable to the rest of the country*.” (Lines 257-259)

3. Please provide additional information on the source of supplementary file titled 'PrEP Toolkit for Providers' and 'Participants Manual'.

The ‘PrEP Toolkit for Providers' and the 'Participants Manual' were developed by the Kenya National AIDS and STI Control Program (NASCOP) in collaboration with implementing partners, who are members of the national PrEP Technical Working Group (TWG). We have provided this clarification in lines 95-98.

4. We have noticed that you have uploaded supporting information but you have not included a list of legends.  Please add a full list of legends for all supporting information files (including figures, table and data files) after the references list.

We have added a list of legends for supporting information after the references list.

5. Since your data is not available for proprietary reasons, please explain via email why the data is not available. Please also include the contact information for the third party organization that should be contacted should other researchers want to request access to this data and please include the full citation of where the data can be found. We also request that you verify with us via email that any researcher will be able to obtain the data set in the same manner that the you have obtained it. If you feel you are unwilling or unable to adhere to this policy, please explain your reasons by return email and your exemption request will be escalated to the editor for approval. Your exemption request will be handled independently and will not hold up the peer review process, but will need to be resolved should your manuscript be accepted for publication. One of the Editorial team will be in touch if they require more information.

Other researchers may request and obtain these data. We have included a data availability statement that reads “*The data from the Partners Scale-Up Project are available by contacting the International Clinical Research Center at the University of Washington (*[*icrc@uw.edu*](mailto:icrc@uw.edu)*).*” (Lines 267-269)

6. Please review your reference list to ensure that it is complete and correct. If you have cited papers that have been retracted, please include the rationale for doing so in the manuscript text, or remove these references and replace them with relevant current references. Any changes to the reference list should be mentioned in the rebuttal letter that accompanies your revised manuscript. If you need to cite a retracted article, indicate the article’s retracted status in the References list and also include a citation and full reference for the retraction notice.

We have reviewed the reference list. It is complete and correct.

**Reviewer #1:**

This is a well written manuscript describing the impact and cost of an on-site multi-component provider training module for oral PrEP provision in Kenya using a before-after design. It underscores the importance of providers as gate-keepers to access to interventions and the importance of these gatekeepers to be well trained and confident to facilitate access to the new technology being introduced. It does require a potential user to arrive at a health facility in the first instance and strategies to enhance uptake need to be reviewed regularly as uptake and coverage increases and the intervention becomes normative.

Two points for consideration:

i. Beyond the basics the needs of potential users vary quite a bit and it would have been great e.g., to have the authors include some inputs on for example strategies on reaching adolescent girls and young women or men through this type of training.

Thank you for this suggestion. The scope of this manuscript was to detail the acceptability, effectiveness and cost of facility-based modular training. The training curriculum focusses on improving knowledge and skills of providers to offer PrEP services in public health facilities.

However, we have observed that well-trained, knowledgeable providers are able to conduct effective health talks at facility waiting bays and have one-on-one consultations with people at risk of acquiring HIV, including adolescent girls and young women or men. Through these activities they inform at risk populations about the benefits of PrEP and encourage them to use this HIV prevention intervention. This is stated in the Discussion, Lines 231-232.

ii. In future 'before-after' impact assessments it may be useful to consider including a group that was not exposed to the 'pre-'/before assessment as this may help mitigate a bias inherent in before-after impact assessments.

Thank you for this comment. We acknowledge this limitation of our study design.

**Reviewer #2:**

1. Introduction –

General comment: The authors chose to study providers modular training as a way to improve PrEP uptake/utilization. This is premised on the fact that inadequate training is one of the barriers to PrEP utilization/uptake, right?

The authors should consider setting the stage by discussing barriers to PrEP uptake/use to give context to the role played by provider education- in other words, strengthen on the why the provider education and the choice of the training re; modular

Thank you for this comment. We have revised the Introduction section. We have described some of the barriers to PrEP uptake including low community awareness, service delivery points that are resource constrained and inaccessible to high risk populations and stigma and discrimination (Lines 50-53). We also highlight the the training gap among health providers (Lines 60-62)

2. Introduction, line 59: the authors describe their approach as innovative. What make this innovative (over other approaches)? Never tried before?

PrEP is a new HIV prevention intervention, that was launched in Kenya in 2017. To improve the capacity of providers to offer services, health workers have been trained on PrEP, but this training has been hotel-based. To our knowledge, facility based PrEP training using modules that are delivered at convenient times for the providers is a novel training approach that addresses challenges associated with usual off-site (often expensive hotel-based), multi-day training and has not been documented previously.

3. Conclusion line 27 and 253: The authors conclude their modular training is effective. In the absence of data (e.g., how many people were being initiated on PrEP pre-training - which is acknowledged in the limitation), and how "effectiveness" was defined in the study, it is difficult to verify that the modular training was effective. The authors should consider reviewing this by providing more context or data to support the effectiveness of the training or tone down on the language

Thank you for this comment. We assert that modular training was effective in imparting knowledge and PrEP delivery skills to providers. This is observed through the increase in scores comparing pre-test and post test scores.

After training, facilities on average initiated an average of ~3 people on PrEP per month and this was sustained over 6 months. We acknowledge the limitation that we have no pre-training data with which to compare PrEP uptake after the training.

We have revised the statement to read, “*On site modular training is an effective way to provide PrEP education for health workers in public health facilities. It is also an acceptable and low-cost approach*”. (Lines 27-28 & 262-264)

4. Line 82: Health facility management determined the providers to be trained? According to a specific guidance? If there was no specific criteria guiding facility managers on how to pick the providers, there is a likely variation in cadres of staff picked by each facility and/or introduce selection bias? This has been pointed as a frequent occurrence in previous trainings as pointed by one of the participants (line 197) - the participant does allude this did not occur in their facility but I think the authors need to be clear on how they manage it

Providers selected for training were those likely to encounter individuals at risk of HIV and those working in departments that offer HIV treatment and prevention services. We have included a statement that reads “*Health providers working in the facility who were likely to come into contact with persons at risk of acquiring HIV or likely to be involved in provision of PrEP services were eligible for training”* (lines 89-91).

Interviews with health providers demonstrated that this training approach enabled many staff members of various cadres attend the training sessions – more individuals and of more cadres than is often done for trainings in this setting. Thus, this training approach may have addressed the bias that may exist when specific cadres or specific individuals are picked by managers for limited training slots at usual hotel-based trainings*.*
